# Common Mechanism of Activated Catalysis in P-loop Fold Nucleoside Triphosphatases—United in Diversity

**DOI:** 10.3390/biom12101346

**Published:** 2022-09-22

**Authors:** Maria I. Kozlova, Daria N. Shalaeva, Daria V. Dibrova, Armen Y. Mulkidjanian

**Affiliations:** 1 School of Physics, Osnabrueck University, D-49069 Osnabrueck, Germany; 2 Center of Cellular Nanoanalytics, Osnabrueck University, D-49069 Osnabrueck, Germany

**Keywords:** Walker ATPase, Walker A motif, Walker B motif, ATPase, Ras GTPase, ATP synthase, helicase, myosin, kinesin, ABC-transporter, G-protein, proton transfer, pK shift, low-barrier hydrogen bond, short hydrogen bond, enzymatic catalysis, aluminium fluoride, Grotthuss proton relay mechanism

## Abstract

To clarify the obscure hydrolysis mechanism of ubiquitous P-loop-fold nucleoside triphosphatases (Walker NTPases), we analysed the structures of 3136 catalytic sites with bound Mg-NTP complexes or their analogues. Our results are presented in two articles; here, in the second of them, we elucidated whether the Walker A and Walker B sequence motifs—common to all P-loop NTPases—could be directly involved in catalysis. We found that the hydrogen bonds (H-bonds) between the strictly conserved, Mg-coordinating Ser/Thr of the Walker A motif ([Ser/Thr]^WA^) and aspartate of the Walker B motif (Asp^WB^) are particularly short (even as short as 2.4 ångströms) in the structures with bound transition state (TS) analogues. Given that a short H-bond implies parity in the pKa values of the H-bond partners, we suggest that, in response to the interactions of a P-loop NTPase with its cognate activating partner, a proton relocates from [Ser/Thr]^WA^ to Asp^WB^. The resulting anionic [Ser/Thr]^WA^ alkoxide withdraws a proton from the catalytic water molecule, and the nascent hydroxyl attacks the gamma phosphate of NTP. When the gamma-phosphate breaks away, the trapped proton at Asp^WB^ passes by the Grotthuss relay via [Ser/Thr]^WA^ to beta-phosphate and compensates for its developing negative charge that is thought to be responsible for the activation barrier of hydrolysis.

## 1. Introduction

**Figure 1:**
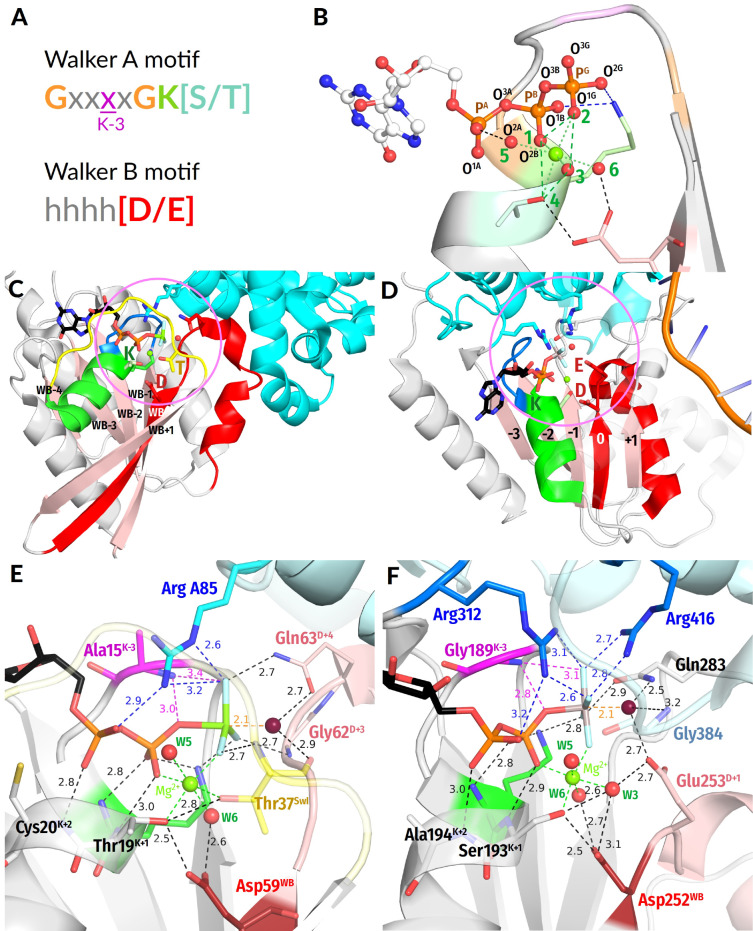
**P-loop-fold NTPases**. (**A**) Conserved Walker motifs in P-loop NTPases. (**B**) Naming of atoms according to IUPAC recommendations for nucleoside triphosphates [48] and typical coordination of Mg^2+^ by ligands, which are numbered in green. (**C**,**D**) Typical folds of P-loop NTPases. (**C**), GTPase Rho with its activator RhoGAP, (PDB ID 1OW3 [44]). (**D**) Chikungunya virus nsP2 SF1-helicase (PDB ID 6JIM [56]). Colour code: Polypeptide chains of P-loop domains are shown as grey cartoons; polypeptide chains of the activating partner are shown as cyan cartoons; the β-strands are coloured pink; α_1_-helix, green; P-loop, blue; Walker B motif and the following residues, red; Switch I loop, yellow. (**E**,**F**) Close-ups of structures shown in (**C**,**D**) as examples of typical catalytic sites of P-loop NTPases. (**E**) GTPase Rho with its activator RhoGAP and the transition state analogue GDP-MgF_3_^−^, (PDB ID 1OW3 [44]). (**F**) Chikungunya virus nsP2 SF1 helicase complexed with transition state analogue ADP-AlF_4_^-^ (PDB ID 6JIM [56]). Colour code: Nucleotides, their analogues, and important amino acid residues are shown as sticks, water molecules as red spheres, the catalytic water molecule W_cat_ is coloured bordeaux, Mg^2+^ ions are shown as lime spheres, the conserved Lys residue of Walker A motif is shown in green with the amino acid residue three residues before it (K-3) highlighted in magenta; the conserved Asp/Glu residue of Walker B motif is shown in dark red with the succeeding residues shown in pale red; Arg fingers are shown in blue/cyan. In the amino acid residues shown as sticks, the oxygen atoms are coloured red, and the nitrogen atoms are coloured blue. In the MgF_3_^−^ moiety, the fluoride atoms are coloured light blue. In the AlF_4_^−^ moiety, the Al atom is coloured gray. The bonds between the stimulator(s) and triphosphate chain are coloured dark blue, the bonds between the backbone amide group of the K-3 residue are coloured magenta, the bond between W_cat_ and the analogue of the P^G^ atom is coloured orange, and the coordinating bonds of Mg^2+^ are coloured.

The hydrolysis of nucleoside triphosphates (NTPs), such as ATP or GTP, by ubiquitous P-loop-fold nucleoside triphosphatases (also known as Walker NTPases) is one of the key reactions in biochemistry. P-loop NTPase domains (see Figure 1 for their overview), which are coded by up to 20% of the gene products in a typical cell, drive the activity of rotary ATP synthases, DNA and RNA helicases, kinesins and myosins, ABC-transporters, ubiquitous translation factors, α-subunits of signalling heterotrimeric G-proteins, and even oncogenic Ras-GTPases [1,2,3,4,5,6,7,8,9,10,11,12,13,14,15,16,17,18,19]. 

**Figure 2:**
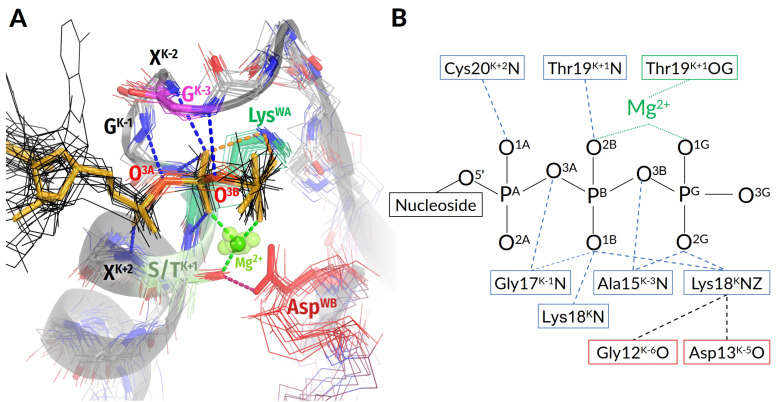
Conserved interactions between Walker A and Walker B motifs across classes of P-loop NTPases. (**A**) Structures of proteins, representing different classes of P-loop NTPases and described in Table 1 are shown superimposed on the P-loop region of the MnmE GTPase with a TS-analogue GDP:AlF_4_^−^ bound (PDB ID: 2GJ8 [35]). For the MnmE GTPase, the P-loop region is shown as a cartoon. The bonding pattern is shown only for the MnmE GTPase. For all the proteins, the last seven residues from the motif GxxxxGK[S/T]x, as well as the Asp residue of the Walker B motif and NTP analogues are shown by lines and coloured as follows: Lys^WA^ in green, Ser/Thr^K+1^ in pale green, Gly/Ala/Asn^K−3^ in magenta, and Asp^WB^ in red. NTP molecules or their analogues are shown in black with bridging oxygen atoms in red. GDP:ALF_4_^−^ from the MnmE structure is shown in orange. Other protein residues are shown in gray with backbone nitrogen and oxygen atoms shown in blue and red, respectively. Mg^2+^ ions are shown as green spheres. The phosphate chain is involved in numerous bonds with backbone amide groups of Walker A motif (the bonds are highlighted in blue), conserved Lys^WA^ contacts O^2G^ and O^1B^ atoms (orange bonds), and O^2B^ and O^1G^ atoms are part of the Mg^2+^ coordination shell (green bonds). (**B**) Coordination bonds and H-bonds between the Walker A motif and Mg-NTP moiety in the MnmE GTPase (PDB ID: 2GJ8 [35]).

The P-loop-fold domain is a three-layer αβα sandwich; see Figure 1 and [10,18,20,21,22,23]. In small P-loop NTPases (Figure 1C), the titular *p*hosphate-binding loop (P-loop) usually connects the first β-strand (β_1_-strand) with the first α-helix (α_1_-helix); the P-loop together with the two first residues of the α_1_-helix have the [G/A]xxxxGK[S/T] sequence, known as the Walker A motif [1]. This motif is responsible for the binding of the triphosphate chain and cofactor Mg^2+^ ion; see [1,2,24] and Figure 1A–D. The Walker B motif *hhhh*D, where ’*h’* denotes a hydrophobic residue, is the other shared motif of P-loop NTPases; see Figure 1A–F and [1,2,24]. In small P-loop NTPases, the conserved aspartate residue of this motif is at the C-terminal tip (hereafter, the C-cap) of the β_3_-strand [9], opposite to the α_1_-helix. 

Although a few P-loop NTPases have a glutamate residue in the respective position, the conserved carboxylic residue of Walker B motif is denoted as Asp^WB^ (and not as [Asp/Glu]^WB^)throughout the manuscript for the sake of simplicity. Asp^WB^ makes a hydrogen bond (H-bond) with the conserved Ser/Thr of the Walker A motif that follows the conserved Lys residue; see Figure 1E and Figure 1F where the respective residues are denoted as Thr19^K+1^ and Ser193^K+1^, respectively.

A specific feature of most P-loop ATPases is their activation before each turnover, so that the NTP hydrolysis proceeds in two steps. First, an ATP or a GTP molecule binds to the Walker A motif and attains a configuration with eclipsed β- and γ-phosphates, as enforced by the Walker A motif and the Mg^2+^ ion; see Figure 1B and [23,25,26,27,28,29]. Hydrolysis, however, takes place only when the P-loop domain interacts with its cognate activating partner, which could be a domain of the same protein, a separate protein, and/or a DNA/RNA molecule. Upon this interaction, specific stimulatory moieties, usually Arg or Lys residues (“Arg/Lys fingers”, Figure 1E,F), are inserted into the catalytic sites and promote the cleavage of γ-phosphate; see the companion article [30] and [4,15,16,29,31,32,33,34,35,36,37,38]. 

The energy of NTP binding drives the “closing” of the catalytic pocket, whereas the energy of hydrolysis is utilized for its “opening”; both these large-scale conformational changes can be coupled to useful mechanical work, so that most cell motors are driven by P-loop NTPases; see, e.g., [4,39,40,41].

Important hints for clarifying the catalytic mechanism of P-loop NTPases are provided by their structures with bound transition state (TS) analogues, such as NDP:AlF_4_^−^ NDP:MgF_3_^−^ or NDP-VO_4_^3−^ complexes [4,31,32,35,37,42,43,44,45,46,47]. The crystal structures with NDP:MgF_3_^−^ or NDP:AlF_4_^−^ bound (see Figure 1E and Figure 1F, respectively) revealed a “catalytic” water molecule W_cat_ near the analogue of the P^G^ atom and almost in line with its bond with the O^3B^ oxygen atom (see Figure 1B for the atom names according to the IUPAC recommendations for nucleoside triphosphates [48]). In the NDP-VO_4_^3−^ complexes, one of the four oxygen atoms of vanadate occupies the position of the catalytic water molecule [4,45]. These structures, in support of earlier suggestions, indicate that the detachment of γ-phosphate is triggered by an apical nucleophilic attack of deprotonated W_cat_ (**OH^--^_cat_**) on the terminal phosphorus atom P^G^; see Figure 1E,F, Figure S1 in the Supplementary Materials and [15,37,46,49,50,51,52,53,54,55].

It remains unclear how the interaction with the stimulating moiety initiates the deprotonation of W_cat_ and what is the fate of the proton that is released upon this deprotonation. The first TS-like crystal structures of small GTPases, such as those shown in Figure 1C,E, did not reveal potential bases among the ligands of W_cat_ [4,31,32,42]. Thus, it was suggested that the proton is accepted directly by γ-phosphate [57,58,59]. In contrast, the crystal structures of most other P-loop NTPases showed that W_cat_ interacts with Glu or Asp residues; these “catalytic” residues were suggested to serve as proton acceptors in these NTPases; see, e.g., Glu253 in Figure 1F and [8,43,55,60]. Also, it remains obscure whether the proton is transferred from these bases to the triphosphate and, if so, how this transfer takes place. 

There has been no consensus on how stimulatory moieties accelerate NTP hydrolysis. It was suggested that they decrease the activation barrier by compensating for the developing negative charge on β-phosphate [54,61], by electrostatically stabilizing the TS [62], by expulsing water from the catalytic pocket [63], or by some unspecific allosteric impact [64]. 

We chose to address these problems through comparative structure analysis of available of P-loop NTPase structures with full-fledged catalytic sites. Our results are presented in two articles. In the first article (see the companion paper [30]), we inspected 3136 structures of Mg-NTP-containing catalytic sites of P-loop NTPases, identified the structures with inserted stimulator(s), and analysed the patterns of stimulatory interactions. 

In most cases, at least one stimulator, by linking the oxygen atoms of α- and γ-phosphates, appears to twist γ-phosphate counter-clockwise by 30–40°; the rotated γ-phosphate is stabilized by a H-bond with the backbone amide group three residues before the conserved lysine residue of Walker A motif (K-3). In the remaining cases, the stimulator(s) only engage(s) the γ-phosphate group and likely pull(s) or twist(s) it. The mechanistic interaction between the stimulator(s) and γ-phosphate appeared to be the only common property, which points to the stimulator-induced twist of γ-phosphate as the trigger for NTP hydrolysis.

Here, in the second article, we combined global computational analysis of H-bonding around Walker A and Walker B motifs in the same 3136 catalytic sites with the manual identification of catalysis-relevant structural elements in to major classes of P-loop NTPases. By focusing on TS-like structures, we attempted to capture the structural transitions involved in catalysis. 

We found that major classes of P-loop ATPases and GTPases share the following common structural features:

(1) In most analysed structures, a short H-bond (<2.7 Å) connects Asp^WB^ and [Ser/Thr]^K+1^; this bond is extremely short (2.4–2.5 Å) in those structures that contain NDP:AlF_4_^−^ as a TS analogue; see Figure 1E,F.
(2) In TS-like structures of P-loop NTPases of all classes, except those of the TRAFAC class, the W_cat_-coordinating “catalytic” Glu or Asp residue provides a proton pathway from W_cat_ to the nearest ligand of Mg^2+^.
(3) The distance between neighboring ligands in the coordination shell of Mg^2+^ is 2.9–3.0 Å, which implies the possibility of proton exchange between all of them.

These common structural traits allowed us to specify the basic mechanism of stimulated catalysis for the whole superfamily of P-loop NTPases as follows:

(i) The twisting γ-phosphate by stimulator(s) should affect the properties of Mg^2+^ ligands including [S/T]^K+1^; we suggest that the functional pKa of [S/T]^K+1^ becomes lower than that of Asp^WB^, and the proton relocates from [S/T]^K+1^ to Asp^WB^.
(ii) The remaining proton vacancy at the anionic [Ser/Thr]^K+1^ alkoxide is refilled by a proton that comes from W_cat_ (or a sugar moiety in some kinases), after which the nascent nucleophilic anion attacks γ-phosphate.
(iii) Upon the breakaway of γ-phosphate, the trapped on Asp^WB^ proton passes by the Grotthuss relay mechanism via [Ser/Thr]^WA^ to β-phosphate and compensates for its developing negative charge, which is thought to be responsible for the activation barrier of hydrolysis [54,61].

Only Walker A and Walker B motifs are conserved throughout all P-loop NTPases. Our scheme attributes the key catalytic functions to their only two strictly conserved residues that are capable of trapping a proton from water—namely, [S/T]^K+1^ and Asp^WB^.

## 2. Materials and Methods

***Global computational analysis of hydrogen bonding in the structures of P-loop NTPases:***

In this paper, as a part of a large-scale computational structure analysis of more than 3600 catalytic sites in P-loop NTPases, we focused on those amino acid residues that could be involved in the hydrolytic transition, specifically on the conserved Asp of the Walker B motif and equally strictly conserved Ser/Thr of the Walker A motif. We developed a protocol to find these residues in the crystal structures and to measure distances between these residues and their functionally important H-bond partners.

As for the analysis presented in in the companion paper [30], the structures were selected among those PDB entries that matched the following criteria: (1) assigned to InterPro record IPR027417; (2) contained an ATP/GTP molecule, non-hydrolyzable analogue of NTP, or a transition-state analogue; (3) contained at least one Mg^2+^, Mn^2+^, or Ca^2+^ ion; and (4) resolution of 5 Å or higher, if applicable. Proteins were assigned to major classes of P-loop NTPases according to their membership in Pfam families [9,10]; see the details in the companion paper [30]. This search yielded 1474 PDB structures with 3666 catalytic sites in them. 

We investigated the reliability of each catalytic site by assessing the integrity of the NTP analogue/presence of γ-phosphate mimic, presence of a P-loop motif Lys residue within 5 Å of the β-phosphate. This analysis yielded 3136 complexes in 1383 structures with various substrates: ATP and GTP, their nonhydrolyzable analogues, and ADP/GDP molecules associated with γ-phosphate-mimicking moieties; see Figure 2 in the companion paper [30].

For quality control, we measured the distances from [Ser/Thr]^K+1^ to Mg^2+^ to ensure the correct binding of the Mg^2+^ and general reliability of the structure resolution at the binding site (i.e., a long distance would indicate a disturbed catalytic site or resolution at the site that is insufficient for our purposes of comparative analysis), and from [Asp/Glu]^WB^ to Mg^2+^, to identify cases of direct coordination of Mg^2+^ by the acidic residue (short distances) or disassembled binding sites (long distances). In addition, in those sites that passed quality control, we examined the presence of interactions of phosphate chain with the **NH**^K−3^ group and positively charged moieties (the details of analysis are described in the companion paper [30] and depicted in its Figure 2).

A putative [Asp/Glu]^WB^ residue was identified as follows: distances from all Asp and Glu carboxyl groups to [Ser/Thr]^K+1^ terminal hydroxyl were measured, and the closest residue that was preceded by at least three non-ionizable residues (Glu, Asp, Ser, Thr, Tyr, Lys, Arg and His were considered as ionizable) was chosen as the partner of [Ser/Thr]^K+1^. This set of data was used for the quantitative analysis. 

If this procedure failed, the nearest Asp/Glu was selected without a hydrophobicity check and used to measure the required distance; the corresponding results are shown in Table S1. Residues located further than 5 Å were not considered. In those few cases where [Asp/Glu]^WB^ residue is indicated in the Table S1 as “absent at the threshold distance”, the catalytic site is likely to be fully “open”. We did not check all these cases manually.

Specifically, the distance from the closest carboxylic oxygen of the selected [Asp/Glu]^WB^ to the [Ser/Thr]^K+1^ hydroxyl oxygen was recorded for each site where a [Ser/Thr] residue could be identified at the K+1 position. 

***Calculation of the solvent-accessible surface area (SASA)*** and relative SASA were performed for Asp^WB^ residues by using the Shrake–Rupley algorithm [65] implementation in PyMol v2.5.0 (solvent radius 1.4Å).

***The pKa values*** of Asp^WB^ in the bovine ATP synthase structures were assessed using the PROPKA 2.0 software [66]. 

***Manual comparative structure analysis***: For each class of P-loop NTPases, representative structures were manually selected from the Protein Data Bank (PDB) at www.rcsb.org (last accessed on 11 June 2022) [67,68] based on the literature data and structure availability. 

***Visualization***: The structure superposition, manual distance measurements, manual inspection and structure visualization were performed with Mol* Viewer [69] and PyMol v2.5.0 [70]. 

***Statistics***: The calculated *p*-values are provided in the Supplementary Statistics File, which also contains a description of the routine used. 

***Scripts and raw data:*** Descriptions of each binding site are available in Table S1. The scripts used to generate and annotate the data and quickly visualize selected sites listed in Table S1 are available from github.com/servalli/pyploop (accessed on 22 June 2022).

## 3. Results

The results section is organized in the following way. In Section 3.1, we introduce the generic designation for the structural elements of P-loop NTPases. Without this generic nomenclature, our structural comparison of NTPases of diverse classes would hardly be possible.

Section 3.2 is devoted to identification of structural features that are common for different classes of NTPases. The mechanism of triphosphate chain stabilization, which is similar in NTPases of all classes, is considered in Section 3.2.1. Other structural elements of P-loop NTPases, however, vary among enzyme families; each class of P-loop NTPases is characterized by its specific constellation(s) of stimulators and W_cat_-coordinating residues. Since no detailed comparative survey of these structural attributes in all the multitude of P-loop NTPases is available, we provide such a survey for representatives of major classes of P-loop NTPases in Section 3.2.2 and summarize our findings in Table 1. 

**Figure 3:**
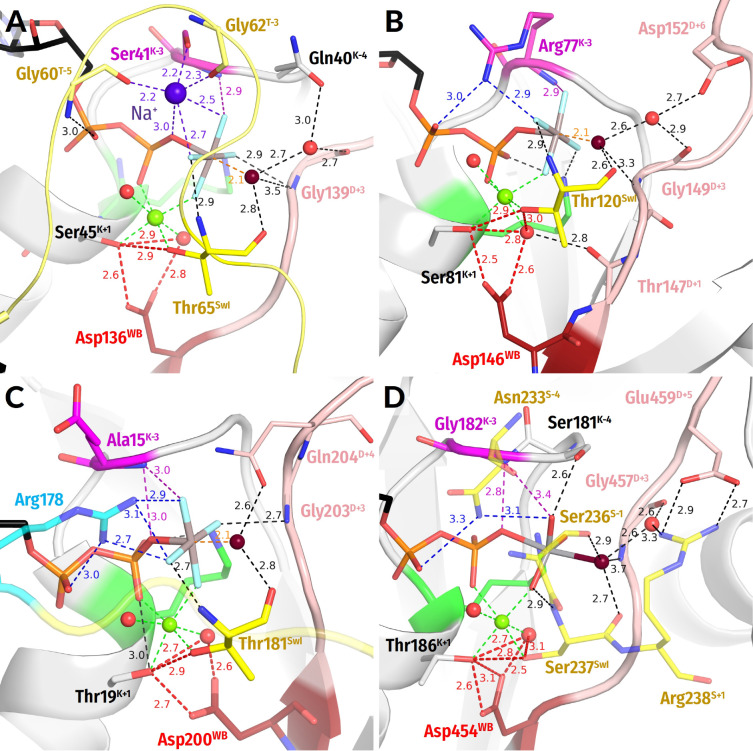
**Representative NTPases of the TRAFAC class.** The residues of Switch I/K-loop are shown in yellow, and the Na^+^ ion is shown in blue. The tentative proton routes are shown by thick red dashed lines. Other colours as in Figure 1E,F. All distances are given in ångströms. (**A**) Dynamin (PDB ID 2X2E, [72]). (**B**) Atlastin-1 (PDB ID 6B9F, [92]). (**C**) Gα_12_ subunit of a heterotrimeric G-protein (PDB ID 2ODE, [84]). (**D**) Myosin II of *Dictyostelium discoideum* (PDB ID 1VOM [93]).

**Figure 4:**
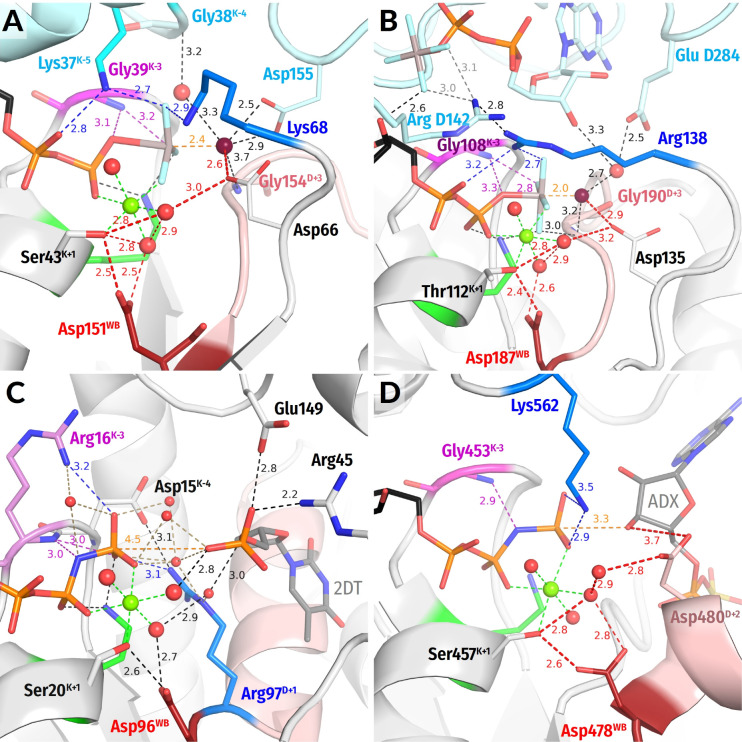
**Representatives of the SIMIBI and kinase classes.** The Arg and Lys residues belonging to the same protein chain as the Walker A motif, i.e., to the lid domains in kinases, are shown in blue. Red dashed lines mark protonic connections between W_cat_ and Asp^WB^. Other colours as in Figure 1E,F and Figure 3. All distances are indicated in ångströms. (**A**) ATP-binding component of the dark-operative protochlorophyllide reductase (PDB ID 2YNM, [97]). (**B**) The signal recognition particle (FtsY/Ffh) complex (PDB ID 2CNW, [98]). (**C**) Human thymidylate kinase (PDB ID 1NN5 [99]). (**D**) Adenosine 5′-phosphosulfate kinase (PDB ID 4BZX, [100]).

**Figure 5:**
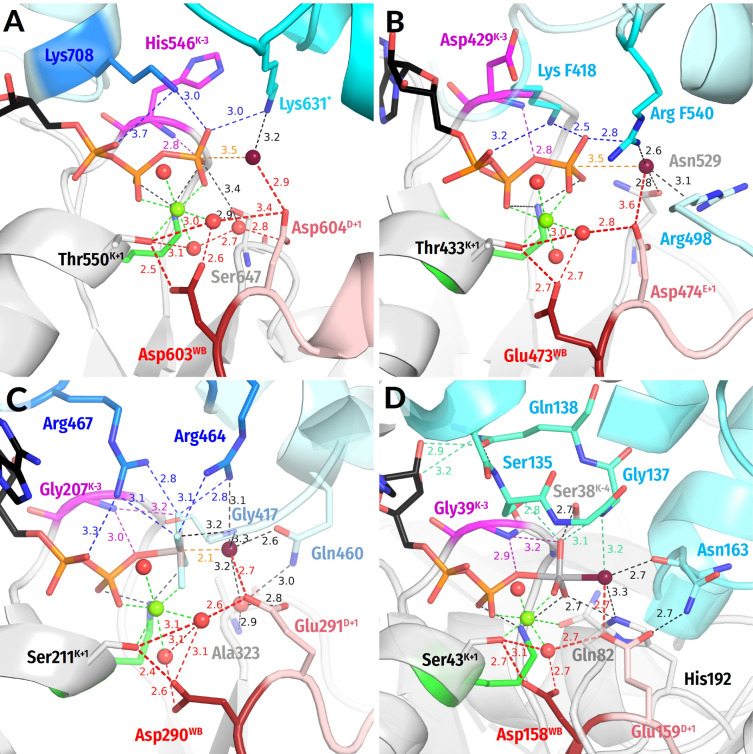
**Representatives of AAA+ ATPases, SF3 helicases, SF2 helicases, and ABC ATPases**. The Arg residues from the C-terminal helical domain of the same monomer are shown in deep blue (panel (**C**)). The LSGGQ motif is shown in green-cyan, and the VO_4_^3—^ moiety in grey and red (panel (**D**)). Red dashed lines mark protonic connections between W_cat_ and Asp^WB^. Other colours are as in Figure 1E,F and Figure 3. All distances are given in ångströms. (**A**) N-ethylmaleimide sensitive factor (PDB ID 1NSF [109]). (**B**) Replicative hexameric helicase of SV40 large tumor antigen (PDB ID 1SVM, [117]). (**C**) Hepatitis C virus NS3 SF2-helicase (PDB 5E4F [118]). (**D**) The outward-facing maltose transporter complex with ADP—VO_4_^3—^ bound (PDB ID 3PUV, [116]).

**Figure 6:**
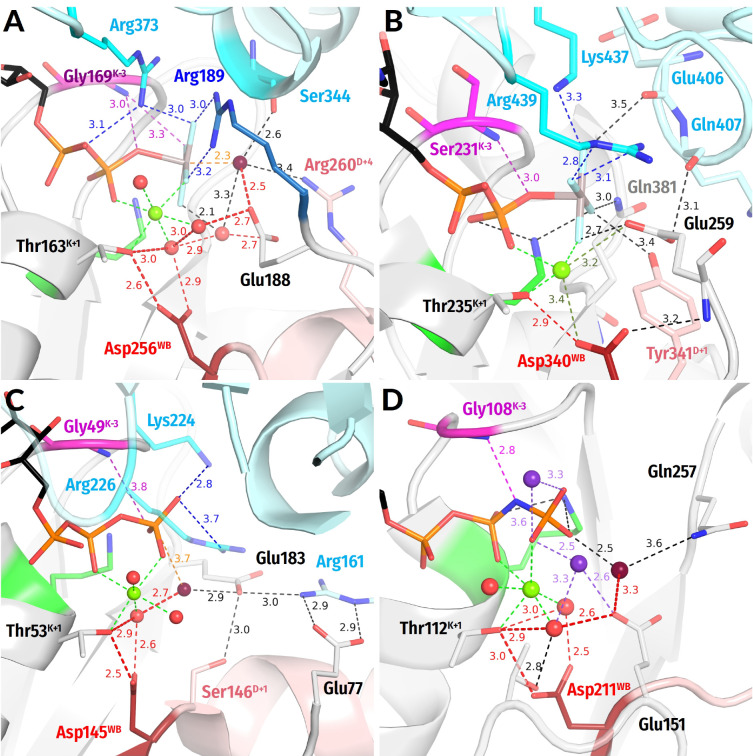
**Representative proteins of the RecA/F_1_-like class of the P-loop NTPases.** The Arg finger as provided by the P-loop domain proper is shown in deep blue (panel (**A**)); K^+^ ions are shown as purple spheres (panel (**D**)). Red dashed lines mark protonic connections between W_cat_ and Asp^WB^. Other colours as in Figure 1E,F and Figure 3. All distances are in ångströms. (**A**) Bovine F_1_-ATPase (PDB ID 1H8E, [43]). (**B**) DnaB replicative helicase from *Vibrio cholerae* (PDB 6T66 [129]). (**C**) Circadian clock protein KaiC (PDB ID 4TL7, [123]). (**D**) RadA recombinase (PDB ID 3EW9, [130]).

Section 3.2.2 is rather large. Therefore, those readers with little interest in the features of specific families and classes of P-loop NTPases can skip this section and simply consult Table 1 and the subsequent Section 3.2.3. This section summarizes the previously unreported common properties of P-loop NTPases that we have inferred, in Section 3.2.2, from examination of representative structures shown in Figures 3–6 and described in Table 1. Anyhow, most of these structures are referenced throughout the following sections so that the reader will have the opportunity to admire the ingenuity of nature and beauty of these enzymes.

In Section 3.2.4 we analyse the patterns of water molecules around the coordination shell of Mg^2+^ ion in P-loop NTPases. 

Section 3.3 is devoted to the global computational analysis of H-bonding between the Walker A and Walker B motifs.

### 3.1. Generic Designation of Structure Elements in P-loop Fold NTPases

In the companion article [30], we introduced and used a generic amino acid numbering for the conserved regions of P-loop NTPases. Hereafter, we use the same nomenclature and number the amino acids residues of the Walker A, Switch I, and Walker B motifs (including the functionally relevant residues following the Walker B motif) relatively to strictly conserved reference residues—namely, Lys^WA^ (K^WA^) of the Walker A motif, Asp^WB^ (D^WB^) of the Walker B motif, and [Thr/Ser]^SwI^ ([T/S] ^SwI^) of the Switch I motif, respectively, as shown in Figure 1 (see the companion article [30] for further details). In this numbering, the “catalytic” glutamate residue that follows Asp^WB^ in the sequence of many P-loop NTPases is labeled as Glu^D+1^. In those rare cases where a Glu residue serves as Asp^WB^, the subsequent residue is denoted as Xxx^E+1^. 

For ease of reference, we also introduce and use, hereafter, a novel generic numbering for the strands of the β-pleated sheet (for comparison, the conventional numbering of β-strands is shown in Figure S2 for major classes of P-loop NTPases). The need for such a general numbering comes from the variable position of the Walker B motif-containing β-strand in the amino acid sequence of diverse P-loop NTPases. Indeed, in small P-loop NTPases, the Walker B motif is at the C-cap of the β_3_-strand [9]. However, in other NTPases, it is at the C-cap of other β strands or even at the N-terminus [24]. Furthermore, larger P-loop NTPases can have additional β-strands that precede, in the amino acid sequence, the β_1_-strand that becomes the P-loop. Furthermore, the Walker B motif is located at the C-cap of the β_2_-strand in the shikimate, gluconate, and adenosine phosphosulfate kinases [9]. The respective structures show that the conserved Asp^WB^ residue makes both canonical H-bonds with [Thr/Ser]^K+1^ and the Mg^2+^-coordinating water. However, even recent review papers on these kinases refer to the Walker B motif in the β_3_-strand, where it is conspicuously absent. 

Therefore, hereafter, for simplicity, we call the β-strand, which is in line with the triphosphate chain, the “*Walker B strand*” or *WB-strand*. This strand is easy to find in a structure because it carries an aspartate or, rarely, a glutamate residue close to its C-cap; the carboxyl of this residue is usually H-bonded to the [Thr/Ser]^K+1^ residue of the Walker A motif as shown in Figure 1E,F. In addition, this carboxylic residue is usually preceded by four non-polar amino acids of the Walker B motif; see Figure 1A and [1]. Other strands of the same β−pleated sheet, independently of their position in the amino acid sequence, can be numbered by their position relatively to WB-strand as WB-1 and WB-2 or as WB+1, WB+2, and so on, as shown in Figure 1C. For the sake of brevity, “WB” could be omitted, and the strands could be numbered −1, −2, 0 (for the WB strand), +1, +2, and so on, as depicted in Figure 1D, Figures S3 and S4. 

In those cases where the crystal structures of NTPases with their cognate activating partners are available, these partners, according to our observations, usually interact with a stretch of amino acids that follows the Walker B motif and approximately corresponds to residues from Asp^WB^+1 (aka D+1) to Asp^WB^+12 (aka D+12); this stretch is coloured red in Figure 1C,D and pale red in Figure 1E,F. In the case of small NTPases, this region corresponds to their α_3_-helices (Figure 1C). 

In some publications, the first few amino acids of this stretch are united with the Walker B motif into the “extended Walker B motif”. However, the amino acids beyond the conserved Asp^WB^ residue show no conservation throughout P-loop NTPases (see Figure S3); hence, they make no conserved motif. By its shape, this stretch resembles a cock’s crest and usually rises above the neighbouring β-strands as clearly seen in Figure 1C,D. Hereafter, we will name this structural element the “*Walker B crest*” or *WB-crest*.

### 3.2. Catalytically Relevant Amino Acids, Stimulatory Patterns, and Activation Mechanisms in Different Classes of P-loop NTPases

The P-loop NTPases are thought to form two divisions—namely, the Kinase-GTPase division and ASCE (Additional Strand Catalytic E (glutamate)) division, with both divisions containing several enzyme classes; see Figures S2–S4 and [9,10,12,14,19]. 

For most classes of P-loop NTPases, we selected representatives out of a set of proteins with available crystal structures in the Protein Data Bank (PDB) at www.rcsb.org (last access on 11 June 2022) [67,68]. These structures are listed in Table 1. Each selected structure contains a Mg^2+^ cation and an NTP molecule or its analogue bound; if available, we chose high-resolution structures that contained TS analogues. 

For these NTPases, after assessing the overall interaction with the activating partner, we checked the following features: (i) amino acids that coordinate the Mg-triphosphate moiety; (ii) positively charged, potentially stimulatory moiety(ies) inserted into the catalytic site during the activation (their cumulative analysis is presented in the companion article [30]); (iii) amino acids that interact with W_cat_; and (iv) other auxiliary amino acids that interact with oxygen atoms of the γ-phosphate group (or its analogues). The results of this manual analysis are described below and summarized in Table 1.

#### 3.2.1. Coordination of the Mg-triphosphate Moiety

First, representative structures from different classes were superposed with the structure of AlF_4_^—^-containing, K^+^-dependent GTPase MnmE (PDB ID 2GJ8, resolution 1.7 Å [35]), the activation mechanism of which we scrutinized elsewhere [29]. We aligned 20 amino acids of the β_1_-strand, P-loop, and α_1_-helix with the corresponding amino acids 217–236 of MnmE. The whole shape of the P-loop was found to be strictly conserved across all classes of P-loop NTPases (Figure 2A), in agreement with an earlier observation made with a smaller set of P-loop NTPase structures [4]). Accordingly, the binding mode of the triphosphate chain, which is described below, appears to be conserved throughout P-loop NTPases.

In all manually inspected structures, the NTP molecule (or its analogue) is bound to the P-loop of the Walker A motif in an extended conformation, which is supposedly catalytically prone (Figure 1E,F and Figure 2A); elsewhere, we showed that this conformation is similar to those of Mg-ATP and Mg-GTP in water in the presence of large monovalent cations, such as K^+^ or NH_4_^+^ [29]. The configuration of the catalytic site is enforced by a plethora of conserved bonds that mostly involve the backbone amide groups (hereafter, **HN** groups) of the Walker A motif; see Figure 1A–F and Figure 2A,B and [15,23,71]. 

The partial positive charges of these groups compensate for negative charges of the triphosphate chain oxygens. The structural elements responsible for stabilization of the triphosphate chain by amino acids of the Walker A motif are almost universally conserved (Figure 2). The conservation of invariant residues in the [G/A]xxxxGK[T/S] motif has straightforward reasons: the Gly^K−1^ and Gly^K−6^ residues mark the beginning and end of the P-loop and enable the bending of the backbone; Gly^K−1^, in addition, electrostatically stabilizes the O^3A^ atom of α-phosphate. The Lys^WA^ residue interacts with O^1B^ and O^2G^ atoms and additionally appears to stabilize the P-loop by interacting with backbone carbonyl oxygens of the K−5 and K−6 residues (Figure 2).

In NTPases of all classes except several families of nucleotide monophosphate kinases, the Walker A and Walker B motifs are linked by a H-bond between the side chains of [Thr/Ser]^K+1^ and Asp^WB^ (Figure 1E,F and Figure 2A). This interaction is considered in more detail in Section 3.3.1, where its comprehensive computational analysis is presented.

The same side chain of the conserved [Ser/Thr]^K+1^ serves as the ligand #4 of Mg^2+^. As shown in Figure 1B, other strictly conserved ligands are the O^2B^ and O^1G^ atoms of the triphosphate chain, such as ligands #1 and #2, respectively, and water molecules, such as ligands #5 (W5) and #6 (W6). Position #3 is taken either by water W3 or by diverse amino acid residues in different enzyme families; see [24] for details. The positions of Mg^2+^ ligands are similar over the entire superfamily of P-loop NTPases. 

Figure 2A,B shows that the O^2A^ and O^3G^ atoms of the triphosphate are “free” and not bound to P-loop residues. Not surprisingly, in many TS-like structures, they interact with stimulators (Figure 1E,F). For the MnmE GTPase, our molecular dynamics (MD) simulations showed that the insertion of a K^+^ ion and its simultaneous interaction with O^2A^, O^3B^ and O^3G^ atoms rotates γ-phosphate and leads to the formation of a new H-bond between the O^2G^ atom and **HN** of Asn226^K−3^; see [29] and the companion paper [30]. 

Generally, the position of **HN**^K−3^ in the vicinity of the O^2G^ atom (or the corresponding atom of an NTP analogue) is structurally conserved across P-loop NTPases owing to the highly conserved H-bond of **HN**^K−3^ with the bridging O^3B^ oxygen (Figure 2). In the manually inspected representative structures from Table 1, the distances between **HN**^K−3^ and the closest oxygen atom of γ-phosphate (or its structural analogue) were shorter in the case of TS analogues. A comprehensive computational analysis of **HN**^K−3^-O^2G^ distances in all relevant PDB structures is presented in the companion paper [30].

#### 3.2.2. Variability of Catalytically Relevant Amino Acids, Activating Partners, Stimulatory Patterns, and Coordination of W_cat_

Below, we consider only those families and classes of P-loop NTPases for which the available structures allowed us to obtain unambiguous structural information. The results of this analysis are summarized in Table 1. Those classes of P-loop NTPases for which the structural information is ambiguous are discussed in the Supplementary File S1.

**Kinase-GTPase division***:* The Kinase-GTPase division unites three classes of NTPases: the TRAFAC (from *tra*nslation *fac*tors) class of translational factors and regulatory NTPases, SIMIBI (*si*gnal recognition, *M*inD, and *Bi*oD) class of regulatory dimerizing ATPases and GTPases, and the class of nucleotide kinases (Figures S2 and S3).

In the NTPases of **the TRAFAC class,** the α_1_-helix is followed by an elongated Switch I loop that is specific to the class. The elongated Switch I loop goes into the β-strand, which is antiparallel to all other β-strands of the core β-pleated sheet (Figure 1C and Figure S3). Apart from the TRAFAC class, other classes of P-loop NTPases have predominantly all-parallel core β-pleated sheets. 

The Switch I loop contains the only strictly conserved [Thr/Ser]^SwI^ reference residue with its side chain coordinating Mg^2+^ as the ligand #3 (Figure 1C,E and Figure S3). In most TRAFAC NTPases, the **HN** group of [Thr/Ser]^SwI^ forms a H-bond with γ-phosphate (Figure 1E and Figure 3). The backbone carbonyl group (hereafter, the **CO** group) of [Thr/Ser]^SwI^ interacts with the W_cat_ molecule seen in TS-like crystal structures (Figure 1E and Figure 3). 

The TRAFAC class NTPases show a remarkably broad variety of stimulatory interactions; they are listed in Table 1; shown in Figure 1C,E and Figure 3; and described below.

***Monovalent cation-dependent NTPases***, which we have considered elsewhere [29], are usually stimulated by K^+^ ions, such as the aforementioned GTPase MnmE; see Figure 2 and Figure S3A, Table 1, and [35,36]. The K^+^ ion in MnmE GTPase is coordinated by O^2A^, O^3B^, and O^3G^ atoms of the triphosphate chain, two **CO** groups of the K-loop, and the side chain of Asn^K-3^; see Figure 2A, [29,35] and the companion article [30]. 

In the unique eukaryotic protein family of dynamins, NTP hydrolysis can be stimulated by either K^+^ or Na^+^ ions [72]. Here, a Na^+^ or a K^+^ ion interacts only with the O^3B^ and O^3G^ atoms but does not reach the O^2A^ atom; see Figure 3A, the companion article [30], and [29,36] for details. 

These K^+^/Na^+^-stimulated TRAFAC class NTPases belong to the family of HAS (hydrophobic amino acid substitution) NTPases [73]. No hydrophilic side chains are present in the vicinity of W_cat_; as indicated in Table 1, W_cat_ interacts only with the nearby atoms of the protein backbone, e.g., **CO**^SwI^, **HN**^SwI^, and **HN**^D+2^ in the case of the MnmE GTPase (see also the companion paper [30]). In dynamins (Figure 3A), these are the side chains of Gln^K−4^, **CO**^SwI^, and **HN**^D+3^.

The titular family of ***translation factors*** unites ribosome-dependent GTPases that are directly involved in the translation, such as the elongation factors EF-Tu and EF-G (Figure S3B, Table 1). These GTPases showed K^+^-dependence both when studied in the absence of the ribosome [74,75] and under the conditions of protein synthesis [76,77,78,79]. 

Earlier, we suggested that these translational GTPases, similar to K^+^-dependent GTPases, are stimulated by a K^+^-ion bound between the side chain of Asp^K−3^ and **CO** of Gly^T−2^ [80]. More recently, crystal structures of these GTPases indeed showed a monovalent cation in the predicted position [81]. 

In the translational GTPases, W_cat_ is uniquely stabilized by the side chain of His^D+4^ residue in addition to **HN** of Gly^D+3^ and **CO** of Thr^SwI^; the side chain of His turns towards W_cat_ in response to the activating interaction of the WB-crest with the small ribosomal subunit and tRNA [82].

In ***GB1/RHD3-type GTPases*** (e.g., atlastins) the stimulatory Arg finger is in the K−3 position of the P-loop and links the O^2A^ and O^3G^ atoms of GTP when two protein monomers dimerize in response to the interaction with the activating partner (PDB ID 4IDQ [83], Figure 3B). In this case, Arg fingers stimulate GTP hydrolysis in the same P-loop domain that they belong to. In the case of atlastins, W_cat_ is seen to be stabilized by **CO**^SwI^ and **HN**^D+3^ (Table 1, Figure 3B).

In the GTPase domains of ***α-subunits of heterotrimeric G-proteins***, the intrinsic Arg finger, as provided by a family-specific insertion domain, links O^2A^ and O^3G^ atoms of GTP and is on a H-bond compatible distance from the O^3B^ atom; see Figure 3C and [5,32,84]. The W_cat_ is stabilized by Gln^D+4^ and **HN**^D+3^ of the WB-crest as well as by **CO**^SwI^; see Figure 3C, Table 1, and [32,84].

The family of ***Ras-like GTPases*** named after its oncogenic members (from *rat sarcoma*), is one of the best-studied groups in the TRAFAC class [15,38,39,85]. In these proteins, the Switch I loop interacts with diverse physiological modulators of activity, whereas the specific GTPase activating proteins (GAPs) bind to the WB-crest (also called Switch II in these proteins); see Figure 1C,E, Figure S3C and [15,38,86,87]. In most structures of Ras-like GTPases complexed with their GAPs (Figure 1C,E), similarly to α-subunits of heterotrimeric G-proteins shown in Figure 3C, the NH2 group of the stimulatory Arg finger links the O^2A^ and O^3G^ atoms (see the companion article [30] for the discussion of apparent exceptions). The W_cat_ molecule is stabilized by the side chain of GlnD+4, **HN** of GlyD+3, and **CO**^SwI^; see Table 1 and [15,86,88]. 

In P-loop NTPases of the ***kinesin and myosin families,*** a Ser residue (Ser^SwI^) serves as a reference residue of Switch I; Ser^SwI^ provides its side chain oxygen atom to coordinate the Mg^2+^ ion as ligand #3 (Figure 3D and Figure S3D). In these proteins, the Asn^S−4^ residue inserts between α- and γ-phosphates [89,90,91], so that the side chain amino group of Asn^S−4^ links the O^2A^ and O^3G^ atoms; see Figure 3D and the companion article [30].

Additional coordination of the γ-phosphate appears to be provided by the side chain of [Ser/Thr]^K−4^ of the P-loop and Ser^S−1^ of Switch I. The **CO**^SwI^ group and, likely, the side chain of Ser^S−1^ form H-bonds with W_cat_. One more H-bond with W_cat_ is provided by the **HN** of Gly^D+3^ (Table 1, Figure 3D).

**The SIMIBI class NTPases** include ATPases and GTPases that dimerize upon interaction with the activating partner in such a way that catalytic sites of the monomers interact “face to face”; see Figure S3E and [94,95]. Each monomer inserts either a Lys (Figure 4A) or an Arg residue (Figure 4B) into the catalytic site of the other monomer [94,95]. Lysine and arginine fingers, as used by SIMIBI proteins, form H-bonds with both O^2A^ and O^3G^ atoms (Figure 4A,B). Many SIMIBI class NTPases display similar activation patterns, where an NTP-bound (homo)dimer demands the interaction with an activating protein (or RNA in SRP/SR complexes) to bring the specific “catalytic” residue(s) closer to the catalytic site where they can contribute to the H-bond network around W_cat_ [94,96].

Signal recognition particles (***SRPs***) and their cognate receptors ***(SRs***) stand separately within the SIMIBI class. Their GTPase domains form a pseudo-homodimer, where the two GTP binding sites interact face-to-face; however, GTP hydrolysis occurs in only one of the two monomers. These proteins employ Arg residues that are inserted reciprocally so that the guanidinium group interacts with the α- and γ-phosphate of the GTP molecule bound by “its” subunit and the α-phosphate of the GTP molecule bound by the other subunit.

Many SIMIBI proteins have a Gly^D+3^ residue that appears to provide its **HN**^D+3^ for coordination of the γ-phosphate, similarly to Gly^D+3^ in TRAFAC class proteins; see Figure 4A,B and [10]. Additional coordination of the γ-phosphate is provided by amino acids located outside of the conserved motifs. Such residues are protein-family specific and are often introduced into the catalytic site from the adjacent monomer upon the interaction with the activating partner and dimerization (Figure 4A,B). 

The W_cat_ molecule in SIMIBI NTPases is routinely stabilized by **HN** of GlyD+3 and the Asp/Glu residue at the C-cap of the WB+1 strand; the carboxyl group of this “catalytic” residue links W_cat_ with the Mg2+-coordinating W3 molecule (Figure 4A,B). The residues of the other monomer and even the ribose 3′ hydroxyl group of the “other” GTP molecule can contribute to the coordination of W_cat_; see Table 1 and Figure 4A,B. The NTP-bound SIMIBI dimer is believed to require interaction with an activating protein (or RNA in SRP/SR complexes) to bring these specific “catalytic” residue(s) closer to the catalytic site where they can contribute to the H-bond network around W_cat_ [94,96].

**Nucleotide Kinases** are ubiquitous enzymes that usually transfer a γ-phosphoryl residue from ATP to a wide range of “second” substrates, primarily small molecules [9,101]; see Figure S3F. The key roles in the catalysis by P-loop kinases are usually played by Arg or Lys residue(s) located in the so-called “LID” domain, a small helical segment that “covers” the catalytic site and often carries several positively charged residues [9,101,102]; see Figure 4C,D. Thus, P-loop kinases do not need other proteins or domains to provide stimulatory Arg/Lys fingers. Instead, the binding of their second substrate is sufficient to trigger the Lid domain rearrangement that results in the insertion of Arg/Lys finger(s). While Arg residues serve as stimulatory moieties in most kinases (Figure 4C), a Lys residue appears to be involved in adenylylsulfate kinases, where the LID domain inserts a Lys finger that interacts directly with the γ-phosphate and is connected to α-phosphate via a water molecule (Figure 4D) [103]. 

One of the stimulatory fingers usually inserts between the α- and γ-phosphates. As in many other P-loop NTPases, the catalysis in kinases is usually assisted by auxiliary arginine and lysine fingers that position the interacting molecules and neutralize the negative charges of phosphate groups; see Figure 4C,D, Table 1 and [46,104]. In thymidylate kinases, the Arg^D+1^ finger is assisted by Arg^K−3^, similarly to atlastins, cf Figure 3B and Figure 4C.

In kinases, the hydrolysis of the phosphoryl donor molecule is mediated by the acceptor molecule, which, similarly to W_cat_ in other reactions of NTP hydrolysis, appears to initiate the nucleophilic attack and formation of the pentavalent intermediate. In nucleotide monophosphate kinases, the attacking group is a negatively charged phosphate moiety that requires no specific proton acceptor (Figure 4C). In those kinase families, where the deprotonation of the attacking molecule is needed to produce a nucleophile, the [Asp/Glu]^D+2^ residue appears to serve as an immediate proton acceptor; see Figure 4D and Table 1. This residue links the would-be nucleophilic group with the Mg^2+^ ligand in position 3—usually a water molecule (Figure 4D).

**ASCE division*:*** The NTPases of the ASCE division have all-parallel β-pleated sheets with an inserted, as compared to the sequences of the Kinase-GTPase division NTPases, β-strand in the WB+1 position; see Figure 1B, Figures S2 and S4. In many enzyme classes of this division, further additional β-strands have been identified; see Figures S2 and S4 and [14,18,19]. For the coordination of W_cat_, in addition to class-specific residues, ASCE NTPases typically use a “catalytic” glutamate residue that either directly follows the conserved Asp^WB^ or is located at the C-cap of the WB+1 strand, similarly to SIMIBI NTPases. 

These two features define the name of this division: *additional strand* and*catalytic E* (ASCE) [10,12,105,106]. According to current views [14,18,19] and the most recent phylogenetic scheme depicted in Figure S2, the ASCE NTPases are divided into two clades that differ by the number of β-strands in their P-loop domains. The group of “middle-size” domains with up to five-six β-strands includes AAA+ ATPases, helicases of superfamily 3 (SF3), as well as STAND and KAP ATPases. The clade of “large” ATPases domains, with many β-strands, includes helicases of superfamilies 1 and 2 (SF1/2), ABC-ATPases, RecA/F_1_ ATPases, VirD/PilT-like ATPases, and FtsK-HerA-like ATPases. 

**AAA+ATPases** are *ATPases associated with various cellular activities*, where “+” stands for “extended”; see Figure 5A and Figure S4A. These enzymes contain an N-terminal P-loop domain and an additional α-helical C-terminal domain; see [6,12,13,16,34,107,108] for comprehensive reviews. The P-loop domain of the AAA+ ATPases carries conserved Arg/Lys residue(s) from the side that is opposite to the P-loop. 

The P-loop domains interact upon oligomerization (most often a hexamer is formed), so that the nucleotide-binding site of one subunit receives the Arg/Lys finger(s) from the neighbouring subunit (Figure 5A,B) and/or, in some protein families, an additional Arg/Lys residue from its own C-terminal helical domain (Figure 5A); see [16]. One of the stimulatory residues interacts with γ-phosphate and is called “finger” whereas the other one occupies the space between α- and γ-phosphates and is called “sensor 2”. We could not find structures of AAA+ ATPases with bound TS analogues; based on the available data on site-specific mutants [16], W_cat_ appears to be coordinated by the “catalytic” [Glu/Asp]^D+1^ residue, Arg/Lys finger and, perhaps, the Asn/Ser/Thr residue at the C-cap of the WB-1 strand (“sensor 1”; see Figure 5A). 

As shown in Figure 5A, Glu^D+1^ connects a would-be W_cat_ with Mg^2+^-coordinating W3 in the structure of N-ethylmaleimide sensitive factor (PDB ID 1NSF [109]). AAA+ NTPases are rather diverse, and they can use further polar residues to interact with W_cat_ or γ-phosphate. While the Arg/Lys finger that interacts with γ-phosphate is usually present, the sensor 1 or sensor 2 residues are absent in some enzyme families, as reviewed in [6,12,13,16,34,107,108]. The topology of domains that interact with the P-loop domain can vary. The helicases of superfamily 3 (SF3 helicases) have a different topology of their C-terminal helical domain [13]. Their other specific feature is the presence of a Glu^WB^–Asp^E+1^ pair at the C-cap of the WB strand; the Asp^E+1^ residue builds a link to the Mg^2+^-coordinating W3 molecule (Figure 5B).

**SF1/2 class helicases** are mostly monomeric or dimeric with each polypeptide chain containing two P-loop-fold domains; see [3,110] for reviews. While SF1 and SF2 helicases differ in the structural motifs that couple their DNA/RNA binding sites with ATP-hydrolyzing catalytic pockets, the pockets proper are quite similar (cf Figure 1F and Figure 5C, respectively). In both SF1 and SF2 helicases, the ATP molecule binds to the functional Walker A and B motifs of the N-terminal domain. The C-terminal domain, although it has a P-loop-like fold, lacks the Walker A and B motifs. Following the interaction with an RNA or a DNA molecule, two Arg residues of the C-terminal domain are usually inserted into the ATP-binding site [111]. One of the stimulatory Arg residues of SF1/SF2 helicases forms H-bonds with both α- and γ-phosphates, whereas the other Arg residue interacts with γ-phosphate (or its analogue) and W_cat_. 

The W_cat_ molecule is coordinated by the “catalytic” Glu^D+1^ residue and class-specific Gln and Arg residues (Figure 1F and Figure 5C). The same Glu^D+1^ residue links W_cat_ with the Mg^2+^-coordinating W3 molecule (Figure 1F and Figure 5C and Table 1). 

**ABC (*ATP****-****binding cassette*) ATPases** are multidomain proteins that usually operate as homo- or heterodimers; see Figure 5D and Figure S4B and [112,113]. Their ATP-hydrolyzing domains are usually used to drive large scale conformational changes, e.g., in membrane-embedded ABC-transporters or in water-soluble complexes involved in DNA repair or translation [19,114,115]. 

In dimers of ABC-NTPases, the nucleotide-binding sites of P-loop domains are located on the interface between the monomers, in the same way, as in dimers of SIMIBI NTPases, cf. Figure 5D with Figure 4A,B. Instead of an Arg or Lys residue, each monomer inserts a whole signature motif LSGGQ into the catalytic pocket of the other monomer (Figure 5D and Figure S4B); see also the companion article [30] for details. 

The structures with TS analogues bound are available for the maltose transporter complex; see Figure 5D and [116]. In these structures, the side chain of serine and **HN** of the second glycine residue of the signature motif interact with the O^3G^ atom of γ-phosphate. The side chain of serine is located between the α- and γ-phosphates in the position of the Na^+^ ion in dynamin-like proteins; cf Figure 5D with Figure 3A.

Several amino acids commonly found in the catalytic sites of ABC transporters can stabilize W_cat;_ see Figure 5D and Table 1. In the case of maltose transporter, these are a histidine residue at the C-cap of the WB−1 strand and Glu^D+1^; the latter, in addition, links W_cat_ with the Mg^2+^-coordinating W6 molecule. In the maltose transporter, the activating monomer in a dimer contributes to the coordination of W_cat_ by providing a backbone **CO** group of a residue that is located outside of the signature motif; see Figure 5D and [116].

**The RecA/F_1_ NTPases** class encompasses oligomeric ATP-dependent motors involved in homologous recombination and DNA repair (RecA and RadA/Rad51), catalytic subunits of rotary F/N- and A/V-type ATP synthases, helicases of superfamilies 4 and 5 (SF4 and SF5), as well as several other protein families [3,105,119]. In NTPases of this class, similarly to most AAA+ ATPases, the stimulatory moiety(ies) is/are inserted in the catalytic site by the P-loop domain of the adjacent monomer (Figure 6 and Figure S4C). 

Our structure analysis showed that the stimulation mechanism appears to differ between rotary F_1_-ATPases/SF5 helicases, on the one hand, and recombinases/SF4 helicases, on the other hand. 

In the ***F_1_-ATPases***, the stimulatory Arg residue of the adjacent monomer interacts with both α- and γ-phosphates, whereas an additional, intrinsic Arg residue coordinates γ-phosphate, approaching it apically (see Figure 6A and Figure S4C and Table 1). A similar stimulation mechanism is realized in ***SF5 helicases***; see, e.g., the Rho helicase (PDB ID 6DUQ [120]). 

The W_cat_ molecule is seen in the catalytic apical position only in the ADP:AlF_4_^−^ -containing structure of the bovine F_1_-ATPase (PDB ID 1H8E, Figure 6A) [43]. While, in most ASCE ATPases, the W_cat_-coordinating Glu^D+1^ residue directly follows the Asp^WB^ of the Walker B motif, the W_cat_-coordinating Glu188 of the F_1_-ATPase, similarly to SIMIBI NTPases, is at the C-cap of the WB+1 strand; within the ASCE division, this feature is specific for the RecA/F_1_ class members [8]. This glutamate residue also links W_cat_ with the Mg^2+^-coordinating W3 molecule (Figure 6A). The W_cat_ molecule is also stabilized by Arg260^D+4^; this residue concurrently interacts with the activating neighbouring monomer, which also provides a **CO** group to stabilize W_cat_.

In ***RecA-like recombinases*** and ***SF4 helicases***, two stimulatory positively charged moieties interact only with the γ-phosphate group, in contrast to F_1_-ATPases. In several families, the adjacent subunit provides one Lys residue and one Arg residue that form a short KxR motif [121], with both residues reaching only γ-phosphate. These are, for instance, the bacterial helicase DnaB (Figure 6B; see also [122]), circadian clock protein KaiC (Figure 6C [123]), and gp4d helicase from the T7 bacteriophage (PDB ID 1E0J, [124]). 

In bacterial RecA recombinases, the adjacent monomer in the homooligomer provides two Lys residues that approach the phosphate chain laterally and interact with γ-phosphate as in the RNA recombinase of *E. coli* (see Table 1 and PDB ID 3CMX, [125]). In archaeal and eukaryotic RadA/Rad51-like recombinases, the positions of terminal groups of stimulatory Lys/Arg residues are occupied by two K^+^ ions, which appear to interact with γ-phosphate; see Figure 6D and [121,126]. 

In the case of RecA-like ATPases, the AlF_4_^−^-containing structures of the bacterial DnaB helicase of *Vibrio cholerae* (Figure 6B), *Bacillus stearothermophilus* (PDB ID 4ESV, [127]), and RecA recombinase of *E. coli* (PDB ID 3CMX, [125]) are available. However, the apical water molecule is absent from these structures, the possible reasons of which are discussed in [122]. Therefore, the full set of W_cat_-coordinating groups remains unknown for RecA-like ATPases; the available structures imply the participation of glutamate residues at the C-terminus of the WB+1 strand (Figure 6B–D), as in other RecA/F_1_ class enzymes. The same residue appears to link the would-be W_cat_ with the Mg^2+^-coordinating W3 molecule (Figure 6B–D). 

Generally, the H-bond networks around γ-phosphate and W_cat_ seem to be richer in RecA/F_1_-NTPases than in other classes of P-loop NTPases. Specifically, the coordination of W_cat_ may potentially also involve the D+1 residue of the WB strand, which is usually a H-bonding Ser/Thr/Asn/Tyr, as well as other polar residues at the C-caps of the WB-1 and WB+1 strands [110,123,128]. 

#### 3.2.3. Interim Summary on the Common Structural Traits of P-loop NTPases

The comparative structural analysis in Section 3.2.2 uncovered the following previously not described common structural features of P-loop NTPases:

(i) In all inspected structures for which TS-like structures are available, some of the amino acid residues that immediately follow Asp^WB^ (at the D+1–D+5 positions of the WB-crest) are involved in catalytic interactions with W_cat_ (Figure 1, Figure 2, Figure 3, Figure 4, Figure 5 and Figure 6 and Figure S5, Table 1 and Table S1).
(ii) In all TS-like structures except those of TRAFAC NTPases, one of the W_cat_-coordinating residues is a “catalytic” Glu or Asp that links W_cat_ with Mg^2+^-ligands in positions 3 and/or 6; see Figure 1E,F and Figure 4, Figure 5 and Figure 6.
(iii) The H-bond distances between Asp^WB^ and [Ser/Thr]^K+1^ appear to be unusually short—up to 2.4 Å in the presence of ADP:AlF_4_^−^ (Figure 1, Figure 2, Figure 3, Figure 4, Figure 5 and Figure 6).

These common structural traits, which are shared by P-loop NTPases of different classes, have been mostly overlooked thus far. 

#### 3.2.4. Water Molecules in the Vicinity of the Mg^2+^ Coordination Shell

The W_cat_ molecule should be deprotonated on some stage of the catalytic cycle; see Figure S1 and [15,37,46,49,50,51,53,54,55,131]. However, the pK_a_ value (hereafter, pK for simplicity) of a water molecule in the catalytic site is 14.0, which hinders the deprotonation both thermodynamically and kinetically. 

The P-loop NTPases, in general, are not alone in their need to deprotonate a water molecule and to use the resulting hydroxyl for hydrolysing the O-P bond. In various other, evolutionarily unrelated Mg-dependent enzymes, Mg^2+^ ions are thought to decrease the pK value of potential nucleophilic water molecules [52,132,133,134,135]. In such cases, usually, the would-be nucleophilic water molecule belongs to the first or second coordination shell of the metal cation. In the case of P-loop NTPases, the Mg^2+^ ion is remote from W_cat_ and can incite its deprotonation only if there is a protonic pathway between W_cat_ and the coordination shell of Mg^2+^. Therefore, we manually searched for connections between W_cat_ (or the would-be W_cat_) and the Mg^2+^-coordinating ligands. 

**Figure 7:**
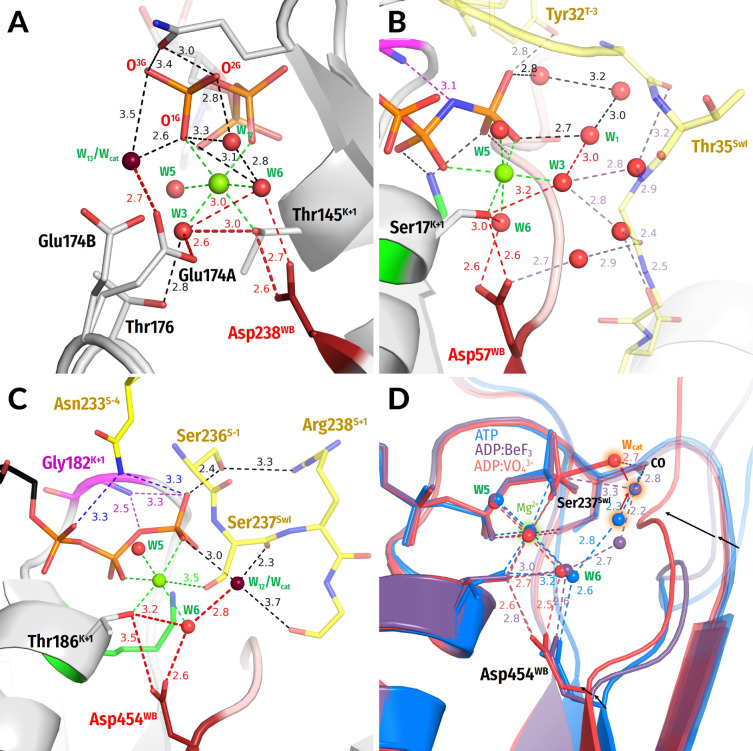
**Networks of water molecules in the catalytic sites of P-loop NTPases**. (**A**) ATP-soaked ATPase domain of RadA from *Pyrococcus furiosus* (PDB ID 4A6X, [136]). (**B**) Human H-Ras GTPase (PDB ID 5B30 [137]). (**C**) ATP-soaked Myosin II from *Dictyostelium discoideum* (PDB ID 1FMW, [140]). Colour code as in Figure 1E,F and Figure 3, Figure 4, Figure 5 and Figure 6. (**D**) Three overlapped crystal structures of myosin II from *Dictyostelium discoideum,* the same as in panel 7(**C**); blue, ATP-soaked structure (PDB ID 1FMW, [140]); violet, ADP:BeF_3_-containing structure (PDB ID 1W9K); red, ADP—VO_4_^3−^-containing, TS-like structure (PDB ID 1VOM [93]); the would-be W_cat_ water molecules are surrounded by orange halos. colour.

In many high-resolution structures of P-loop NTPases with bound substrate molecules or their analogues, water molecules are present in the vicinity of γ-phosphate and interact with the O^1G^, O^2G^, and O^3G^ atoms of γ-phosphate as seen in Figure 7. In principle, a water molecule can interact with up to two oxygen atoms, which allows categorizing the γ-phosphate-bound water molecules as, for example, W_12_, W_13_, or W_23_. Most likely, one of these molecules becomes the W_cat_ upon activation. 

For instance, Figure 7A shows a structure of RadA from *Pyrococcus furiosus* that was post-soaked with ATP; see PDB ID 4A6X and [136]. The structure clearly shows W_12_ and W_13_ molecules and resolves two conformations of the “catalytic” Glu174 of the WB+1 strand. In both conformations, Glu174 interacts with W_13_, the apparent would-be W_cat_ in this enzyme. However, only in the Glu174A conformation, the side chain of Glu174 connects W_cat_ with the Mg^2+^-coordinating W3 molecule. 

Similarly, the “catalytic” Glu or Asp residues link W_cat_ with Mg^2+^-ligands #3 or #6 in TS-like structures of NTPases of all classes (except the TRAFAC class), as already repeatedly noted throughout the Section 3.2. These tentative proton pathways from W_cat_ towards Mg^2+^-ligands are shown by red dashed lines in Figure 4A,B,D, Figure 5A–D, Figure 6A,C,D and Figure 7A. 

In the case of TRAFAC class NTPases, which lack the “catalytic” Glu/Asp residues, the connections between W_cat_ and the Mg^2+^-coordinating ligands are not as evident. The water molecules around γ-phosphate appear to be highly mobile and hardly crystallizable. For instance, in TS-like structures of small GTPases of the TRAFAC class, the Mg^2+^-coordinating ligand that is closest to W_cat_ is usually [Ser/Thr]^SwI^ of Switch I, which is at about 4–5 Å from W_cat_ (Figure 1E and Figure 3A–D). 

In these proteins, however, the Switch I motif is dynamic, and thus the protein fluctuates between “open” and “closed” conformations even when a substrate or its analogue is bound [137,138,139]. In the closed conformation [Ser/Thr]^SwI^ serves as ligand #3 for Mg^2+^ (Figure 1E and Figure 3A–D). In the open conformation, however, the whole Switch I loop moves away from Mg^2+^, and a water molecule serves as ligand #3 (Figure 7B and Figure S5B). 

Notably, Matsumoto and colleagues succeeded in controlling the open-to-close transition upon the crystallization of H-Ras GTPases by changing the humidity [137]. The structure of H-Ras that crystalized in the open conformation at low humidity (PDB ID 5B30) reveals an additional water molecule next to W3 (Figure 7B). It is depicted as W_1_ and can mediate proton exchange with the would-be W_cat_ molecules also seen in Figure 7B. In the closed conformation (Figure 1E and Figure 3A–D), Switch I directly coordinates Mg^2+^ and ousts W_1_ and W3; only W_cat_ is present in the TS-like structure (cf Figure 1E and Figure 7B).

In the absence of TS-analogues, those water molecules that interact with γ-phosphate in TRAFAC class NTPases are often resolvable in the structures if constrained by additional interactions. In the ATP-soaked myosin structure in Figure 7C (PDB ID 1FMW [140]), W_12_ is resolved because it is additionally bound to **CO** of Ser237^SwI^. This W_12_ molecule is at a H-bond-compatible distance from the Mg^2+^-coordinating W6 molecule. Figure 7D shows three overlapped structures of *Dictyostelium discoideum* myosin—namely, the red ATP-soaked myosin structure that is the same as in panel 7C (PDB ID 1FMW [140]), the violet structure with a substrate analogue ADP:BeF_3_ bound (PDB ID 1W9K), and the red TS-like structure with ADP: VO_4_^3–^ bound (here an oxygen atom of VO_4_^3–^ mimics W_cat_ in the apical position (PDB ID 1VOM [93]). 

One can see how a water molecule, which is stabilized by **CO** of Ser237^SwI^ in all three structures, “moves” towards the apical position. It is noteworthy that an additional water molecule links this would-be W_cat_ with the Mg^2+^ ligand sphere in the “intermediate” violet structure. This linking water is seen in several myosin structures and appears to be stabilized by invariant backbone groups of the D+2 residue. In the red TS-like structure, the WB-crest comes closer to the nucleotide and interrupts the water connection with the ligands of Mg^2+^ (Figure 7D). 

### 3.3. Global Structural Analysis of Hydrogen Bonding between the Walker A and Walker B Motifs in the Whole Set of P-loop NTPases with Bound Mg-NTP Complexes or Their Analogs

As described in Section 2, as well as in the in the companion article [30], we extracted from the Protein Data Bank (PDB) at www.rcsb.org (accessed on 11 September 2019) [67,68] those structures of P-loop NTPases that have Mg-NTPs or their analogues bound in the catalytic site, yielding as many as 1383 structure entries with 3136 full-fledged catalytic sites (as of 11 October 2019; many of the structures contained several catalytic sites). The criteria for selection of full-fledged catalytic sites and the routine of their subsequent structural analysis are described and depicted in Section 2 of the companion article [30]. 

As discussed in Section 3.2 and depicted in Figure 2, the shape of the P-loop and the spatial position of Asp^WB^ relatively to it are strictly conserved among P-loop NTPases. Building on that, we were able to use, here and in the companion article [30], the atomic coordinates of the Mg-triphosphate to pinpoint the catalytically relevant residues in an almost sequence-agnostic way, as described in Section 2 of both articles. We applied this approach to perform analysis of nucleotide-binding sites in thousands of structures of P-loop NTPases. The relevant data on all these catalytic sites are collected in Supplementary Table S1 (hereafter, Table S1). 

Based on the type of the molecule bound, the catalytic sites could be sorted into four groups: 1043 sites contained native ATP/GTP molecules; 1612 sites contained bound non-hydrolyzable NTP analogues, such as adenosine 5′-[β,γ-imido]triphosphate, (AMP-PNP), guanosine 5′-[β,γ-imido]triphosphate (GMP-PNP), adenosine 5′-[*β*,*γ*-methylene]triphosphate (AMP-PCP), guanosine 5′-[*β*,*γ*-methylene]triphosphate (GMP-PCP), adenosine 5′-[γ-thio]triphosphate (ATP-γ-S), and guanosine 5′-[γ-thio]triphosphate (GTP-γ-S); 234 sites contained NDP:fluoride complexes mimicking the substrate state, such as NDP:BeF_3_ and NDP:AlF_3_, and 247 sites were with TS analogues NDP:AlF_4_^−^ (204), NDP:MgF_3_^−^ (10), and ADP:VO_4_^3−^ (33). 

#### 3.3.1. The H-Bond between Asp^WB^ and [Ser/Thr]^K+1^ Is Shorter in the Presence of Transition State Analogs

Upon our manual analysis presented in the previous section, we noted that the H-bond between Asp^WB^ and [Ser/Thr]^K+1^ is particularly short in many NDP:AlF_4_^−^-containing representative structures (see Figure 1, Figure 2, Figure 3, Figure 4, Figure 5 and Figure 6). The H-bond between Asp^WB^ and [Ser/Thr]^K+1^ is special because it links the Walker A and Walker B motifs and may be important for catalysis. 

Therefore, we measured the distances between the two residues to characterize their H-bonding in all the available structures of P-loop NTPases with bound substrates or their analogues. The procedure of filtering for structure quality is described in Section 2. The results of these measurements for all the analysed structures are presented in Table S1. The statistical data are provided in the Supplementary Statistics File.

**Figure 8:**
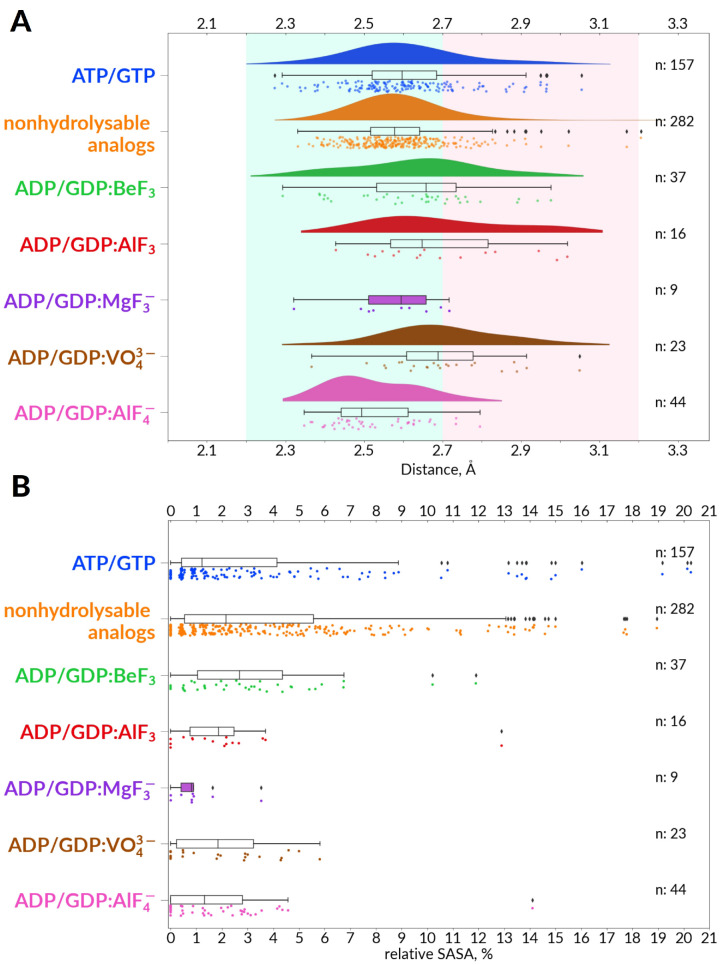
**Properties of Asp^WB^ residues in high-quality in P-loop NTPase structures with bound Mg-NTP molecules or their analogues** (resolution threshold 2.5 Å; see Table S1 for the entire data set). (**A**) Distances between the side chains of [Asp]^WB^ and [Ser/Thr]^K+1^. For each type of complexes, distances are visualized as a kernel density estimate (KDE) plot, a boxplot and individual data points, each point representing one catalytic site in one structure. For ADP/GDP:MgF_3_^−^ complexes the density plot is not shown owing to the scarcity of data. The range of short H-bonds (2.2–2.7 Å) is highlighted in cyan, and the typical H-bond range (2.7–3.2 Å) is highlighted in pale pink. (**B**) Relative solvent-accessible surface area (SASA) values for Asp^WB^ residues. The calculated SASA values are shown for the same catalytic sites as on panel 8 (**A**).

Figure 8A summarizes the data only on the best-resolved structures (with a resolution of 2.5 Å and better) with a typical Walker B motif. The integrity of catalytic sites was checked by two criteria: the [Ser/Thr]^K+1^–Mg^2+^ distance ≤ 2.5 Å and Mg^2+^–Asp^WB^ distance ≤ 6 Å. The data for the three TS analogues are plotted separately for the sake of clarity. For the NDP:AlF_4_^−^-containing structures, we excluded the structures with improperly bound AlF_4_^−^ moieties (see the companion paper [30] for details). 

We consider as short the H-bonds of <2.7 Å [141]; the range of short H-bonds is highlighted in cyan. As seen in Figure 8A, the length of the H-bond between Asp^WB^ and [Ser/Thr]^K+1^ is, on average, <2.7 Å in all groups of structures and <2.6 Å in structures with ATP/GTP, nonhydrolyzable NTP analogues, and such TS-analogues as NDP:MgF_3_^–^ or NDP:AlF_4_^−^ as TS analogues. Specifically, in NDP:AlF_4_^–^-containing structures the H-bond distance is on average as short as 2.5 Å. 

When comparing only TS analogues, fluoride complexes were superior to vanadate complexes as can be judged from the distributions of the Asp^WB^–[Ser/Thr]^K+1^ bond lengths (Figure 8A). From the same data it follows that the NDP:AlF_4_^−^ complexes “overperform” other metal fluorides. In the case of NDP:AlF_4_^−^containing complexes, the average length of the H-bond was 2.5 Å.

It is well known that short H-bonds readily form between buried amino acid residues [142]. Therefore, we determined the solvent accessible surface area (SASA) of Asp^WB^ for the same structures for which the Asp^WB^–[Ser/Thr]^K+1^ bond lengths were measured. As it can be seen in Figure 8B, Asp^WB^ is well buried in the case of TS-like structures, its SASA values are <6%. 

#### 3.3.2. The TS-Analog AlF_4_^−^ Makes More Bonds within the Catalytic Site than MgF_3_^−^ or AlF_3_

Here, we show that the H-bond Asp^WB^—[Ser/Thr]^K+1^ is shortest in the structures with NDP:AlF_4_^-^ complexes bound (Figure 1, Figure 2, Figure 3, Figure 4, Figure 5, Figure 6 and Figure 8). In the companion paper [30], we showed that the distance between the backbone HN^K−3^ group of the P-loop and the O^3G^ atom of γ-phosphate or its analogue is the shortest also in the in the structures with NDP:AlF_4_^−^ complexes bound. All these observations indicate that binding of an NDP:AlF_4_^−^ complex appears to additionally constrict the binding pocket. 

We hypothesized that the unique ability of AlF_4_^−^ to pull the catalytic site together may correlate with its ability to enter more bonds than MgF_3_^−^, AlF_3_, or BeF_3_. We tested this hypothesis by comparing the structures of the same activated complexes with bound NDP:AlF_4_^−^ and NDP:MgF_3_^−^/AlF_3_. We counted and measured all the bonds in the catalytic sites for the two pairs of such structures. One pair of structures consisted of RhoA GTPases in complex with their activator RhoGAP and GDP:AlF_4_^−^ (PDB ID 1TX4, resolution 1.65 Å [143]) or GDP:MgF_3_^−^ (PDB ID 1OW3, resolution 1.8 Å [44]), respectively, bound. 

The other pair was composed of catalytic sites of bovine mitochondrial F_1_-ATPase with bound ADP:AlF_4_^−^ (PDB ID 1H8E, resolution 2.0 Å [43]) or ADP:AlF_3_ (PDB ID 1E1R, resolution 2.5 Å [60]), respectively. In the latter case, the fluoride derivative can be identified as a genuine ADP:AlF_3_ by the similarity of its geometry to that of the Mn-ADP:AlF_3_ complex in the Zika virus helicase (PDB ID 5Y6M [144]); see the companion paper [30] for details.

The findings are summarized in Supplementary Table S2 (hereafter, Table S2), which shows that AlF_4_^−^ forms more bonds with surrounding residues than MgF_3_^−^ or AlF_3_. Specifically, AlF_4_^−^ usually makes two H-bonds, one moderate and one weak, with Lys^WA^, whereas MgF_3_^−^ or AlF_3_ make only one bond. In addition, AlF_4_^−^ makes two H-bonds with **HN**^K−3^, whereas MgF_3_^−^ or AlF_3_ make only one (cf Tables S1 and S2). Furthermore, many of the AlF_4_^-^ bonds are shorter than those of MgF_3_^−^; see Figure 8A, Tables S1 and S2, as well as the companion paper [30]. Apparently, the NDP:AlF_4_^−^ complexes have more potency to constrict the catalytic site compared with other TS analogues. 

## 4. Discussion

Here, and in the companion article [30], we reported the results of a comparative structural analysis of more than 3136 catalytic sites of P-loop NTPases with nucleoside triphosphates or their analogues bound. The aim of the analysis was to deduce the main structural features common to the catalytic sites of P-loop NTPases and use them to elucidate the mechanism(s) of stimulated NTP hydrolysis. Here, we discuss the results of both papers in relation to the hydrolysis mechanism in P-loop NTPases.

In our work, we used the tools of evolutionary biophysics, which assumes that certain structural elements are conserved by evolution because of their functional importance and that the degree of conservation of the structural elements involved should be considered when choosing from several possible biophysical mechanisms consistent with the structural data; see [121,145,146,147,148,149] for some examples of this approach. 

In the case of P-loop NTPases, the *in-silico* approach is particularly justified since the experimental investigation of all members of this vast superfamily would be unrealistic. In addition, P-loop NTPases are unique in that their catalytic repertoire is unusually narrow for such a vast superfamily [11], which increases the chances of finding a common catalytic mechanism.

### 4.1. Structure Comparison of P-loop NTPases

#### 4.1.1. Constriction of the Catalytic Site in the Transition State

The data presented here, as well as data from the companion article (see Figure 3 in [30]), show that binding of TS analogues leads to greater constriction of catalytic sites than binding of ATP, GTP, or other analogues. As it follows from Figure 8 and Figure 3 of the companion paper [30], fluoride complexes are superior to vanadate complexes in their ability to constrict the catalytic site. This is consistent with the fact that the more electronegative fluorine atoms form stronger H-bonds than do oxygen atoms. NDP:AlF_4_^−^ complexes are superior to NDP:MgF_3_^−^ complexes (Figure 8 of the main text and Figure 3 of the companion paper [30]) because, as documented in Table S2, they enter into more bonds.

In some cases, this constriction can be revealed by comparing the structures of same P-loop NTPases with bound substrate/substrate analogues and TS-analogues, respectively; see Figure 7D and Figure S5. From the literature, it is known that the distance between W_cat_ and the analogues of the P^G^ atom is also the shortest in the complexes with NDP:AlF_4_^-^ bound [37,46], which corresponds to the WB-crest moving closer to γ-phosphate in P-loop NTPases. The highest constriction of the catalytic site in the presence of ADP:VO_4_^3−^ as a TS analogue was also reported from the comparative analysis of myosin structures. In this work, the volume of the catalytic pocket was assessed from SASA measured with ADP, ADP:BeF_3_, and ADP:VO_4_^-^ as ligands [150]. 

The constriction also manifests itself in the convergence of the Walker A and Walker B motifs. As it follows from Figure 8A and Table S1, the most H-bonds between the side chains of Asp^WB^ and [Ser/Thr]^K+1^ residues can be categorized as short in those structures that contain ATP/GTP or their non-hydrolyzable analogues. Apparently, these short H-bonds form upon “closing” of the catalytic site in response to the substrate binding. Still, these H-bonds are even shorter in the presence of NTP:AlF_4_^-^ and NTP:MgF_3_^−^ (Figure 8A, Table S1), which indicates a further constriction of the catalytic pocket in the presence of these TS analogues. 

The constriction may by functionally important because it would expel water from the catalytic pocket. Indeed, measurements of the relative SASA for Asp^WB^ in diverse NTPases showed that its solvent accessibility drops below 6% in the presence of TS analogues (Figure 8B). We also have noted that the extent of H-bonding of TS analogues within catalytic sites of P-loop NTPases (Figure 8A, Tables S1 and S2 and Figure 3 of the companion paper [30]) appear to correlate with their ability to induce self-assembly of activated complexes, as described in the literature [37,38,42,46,88,151,152,153,154,155,156,157]. A detailed discussion of this point is presented in the Supplementary File S2. Such a correlation implies that these H-bonds stabilize the TS upon hydrolysis of ATP or GTP. 

In sum, as it follows from Figure 8 and Figure S5, Table S1, and the data in the companion article [30], the structures with bound TS analogues, and specifically, NDP:AlF_4_^−^, have particularly short H-bonds between **HN**^K-3^ and O^2G^, as well as between Asp^WB^ and [Ser/Thr]^K+1^, which suggests an additional constriction of the catalytic site in the TS. 

#### 4.1.2. Coordination of W_cat_ and Auxiliary Interactions 

The W_cat_ molecule is usually stabilized by the combined action of evolutionary conserved and variable moieties. The conserved part is represented by residue(s) of the WB-crest and contains residues that bind either W_cat_ alone or W_cat_ and γ-phosphate, such as Gly^D+3^ and Gln^D+4^ of small GTPases (Figure 3), Asp^D+2^ of many kinases (Figure 4C,D), or Glu^D+1^ of many ASCE ATPases (Figure 5). Variable residues are provided by the C-caps of the β-strands, family-specific protein loops, or even external activating proteins/domains, as documented in Table S1. 

In the Kinase-GTPase division, not only the position but also the nature of W_cat_-coordinating residues varies between classes and families (Figure 1E, Figure 3, Figure 4 and Figure S2); specifically, residues and/or **HN** groups in different positions of WB-crest are involved (Figure 1E,F, Figure 3 and Figure 4, Table 1). In most members of the ASCE division, W_cat_ interacts with the “catalytic” Glu^D+1^ residue of WB-crest (Figure 5, Table 1). In Rec/F1 ATPases, the “catalytic” Glu residue is provided by the WB+1 strand and complemented by varying residues of WB-crest (Figure 6, Table 1). 

In addition, polar, W_cat_-stabilizing ligands may be provided by the activating partners. In some Ras-like NTPases, GAPs can provide both the Arg finger and the W_cat_-stabilizing Gln [158]. In SIMIBI NTPases, the “activating” monomer of the dimer provides not only the Lys finger but also the Asp or a Glu residue that stabilizes W_cat_ (Figure 4A,B). In maltose transporter ABC ATPase (Figure 5D) and F_1_-ATPases (Figure 6A), the adjacent monomer that provides the stimulator also provides the backbone **CO** atom to coordinate W_cat_ (Figure 5D and Figure 6A). Such behaviour, where both the stimulator and the W_cat_-stabilizing residue(s) are controlled by the interaction with the activating partner, might represent a kind of a “two-key mechanism” to better control and prevent accidental unwanted NTP hydrolysis. 

In most cases, other auxiliary positively charged groups, such as **HN** groups and/or additional Arg/Lys residues, are involved in the coordination of the oxygen atoms of γ-phosphate and/or W_cat_ in addition to the “main” stimulator. This is observed in AAA+ NTPases (Figure 5A), many helicases (Figure 1F and Figure 5B,C), ABC NTPases (Figure 5D), hexamers of F_1_/RecA-like ATPase (Figure 6), PilT-like proteins (Figure SF1_2A in the supplementary File S1); see also the accompanying article [30]. These auxiliary residues, indicated for the various classes of P-loop NTPases in Table 1 and Table S1, are poorly conserved even within individual families of P-loop NTPases. 

#### 4.1.3. Summary on Novel Common Structural Traits of P-loop NTPases

Our comparative structural analyses, unraveled the following common structural features of P-loop NTPases:

(i) In P-loop NTPases of different classes, the NTP molecule (or its analogue) is bound by the Walker A motif in a same extended conformation (Figure 2A); this conformation is likely to be catalytically prone [29].
(ii) In agreement with previous data (see, e.g., [39,159]), the activating partner usually binds to the P-loop domain by making new H-bonds and salt bridges with the residues of the WB-crest; see Figure 1C,D and Figure S5. In all inspected structures for which TS-like structures are available, some of the amino acid residues that immediately follow Asp^WB^ (at the D+1–D+5 positions of the WB-crest) are involved in catalytic interactions with W_cat_ (Figure 1, Figure 2, Figure 3, Figure 4, Figure 5, Figure 6, Figure 7, Figure S5, Table 1 and Table S1). The energy of binding of the activation partner seems to be used for pushing W_cat_ towards the P^G^ atom of γ-phosphate, constricting the catalytic site, and inserting the stimulatory moiety (see Supplementary File S2 for a detailed consideration).
(iii) In the companion article [30], we provide evidence that the common trait of all inspected stimulators is their mechanistic interaction with the oxygen atom(s) of γ-phosphate, which may cause its rotation by 30–40°.
(iv) Comparing the structures with bound analogues of ATP/GTP and TS, respectively, we noticed that the binding of ADP:AlF_4_^−^ as a TS-analogue results in greater constriction of catalytic sites than the binding of ATP or GTP. The constriction manifests itself in the shorter distances between Asp^WB^ and [Ser/Thr]^K+1^, which are as short as 2.5 Å on average (Figure 1, Figure 2, Figure 3, Figure 4, Figure 5, Figure 6 and Figure 8A). The distances between **HN**^K-3^ and the analogues of γ-phosphate are also shorter in the structures with ADP:AlF_4_^−^ bound; see Figure 1, Figure 2, Figure 3, Figure 4, Figure 5, Figure 6 and the companion article [30].
(v) In the TS-like configurations of P-loop NTPases of all major classes, except the TRAFAC NTPases, the W_cat_-coordinating “catalytic” Glu or Asp residue links W_cat_ with Mg^2+^-ligands in positions 3 and/or 6; see Figure 1E,F, Figure 4, Figure 5 and Figure 6 and Figure 7A.

As argued in the following section, these features are of key importance for understanding the common mechanism of P-loop NTPases. 

### 4.2. ATP and GTP Hydrolysis by P-loop NTPases

#### 4.2.1. Background on the Catalysis by P-loop NTPases

The cleavage of γ-phosphate by P-loop NTPases is thought to involve a nucleophilic attack of W_cat_/OH^—^_cat_ on the P^G^ atom (Figure S1). However, it is yet unclear whether the reaction proceeds in two steps separated by formation of a metastable intermediate (Figure S1A,B) or in one concerted transition with the pentavalent trigonal bipyramidal (tbp) transition state; see Figure S1C and [15,37,46,49,50,51,52,53,54,55,131]. 

In the case of a two-step mechanism, it is also unclear whether the reaction follows the dissociative S_N_1 pathway (Figure S1A) or the associative S_N_2 pathway (Figure S1B). In the S_N_1 mechanism, the rate-limiting step is dissociation of the terminal O^3B^−P^G^ bond to form a metaphosphate intermediate, which interacts with OH^-^_cat_ yielding inorganic phosphate (P_i_); see Figure S1A. In the S_N_2 mechanism, the rate-limiting reaction is the nucleophilic attack by OH^-^_cat_ on P^G^ and the formation of the pentavalent tbp intermediate, which then dissociates into NDP and P_i_; see Figure S1B. 

The research community is roughly evenly divided between proponents of these three mechanisms; see, e.g., [55,131,160]. However, it is not clear whether the difference between the mechanisms is fundamental in this case. It was experimentally shown that the mechanism of ATP hydrolysis varies even in water; it changes from dissociative in pure water to associative in the presence of positively charged chelators for γ-phosphate [161]. 

Therefore, instead of focusing on these differences, we would like to emphasize the universally recognized common features of the reaction pathways shown in Figure S1, namely:

(1) In any model, the covalent bond between O^B3^ and P^G^ must be destabilized [49,50,51].
(2) The γ-phosphate group undergoes a steric inversion when forming a covalent bond with the nucleophile; see Figure S1 and [49]. Therefore, catalytic interactions should planarize the γ-phosphate [162,163].
(3) Negative charges of the oxygen atoms of γ-phosphate must be compensated to increase the electrophilicity of the P^G^ atom and make it prone to nucleophilic attack [15,37,46,49,50,51,53,54,55,131,163].
(4) Conditions in the catalytic site should enable deprotonation of W_cat_ by a suitable proton acceptor [49,50,51,52,53].
(5) Upon dissociation of the O^B3^–P^G^ bond, a large negative charge on the O^3B^ atom must be effectively compensated as it contributes significantly to the activation barrier [54,61].

By correlating the results of our structural analysis with the literature, we outline below how P-loop NTPases may handle these five tasks.

#### 4.2.2. Catalytic Factors in P-loop NTPases: Destabilization of the O^3B^–P^G^ Bond

Even prior to activation, the NTP molecule is bound within the closed catalytic site in a conformation more extended compared to its conformation in water, as noted earlier for particular enzymes [28,29,163,164]. As Figure 2A shows, this extended conformation of the bound NTP molecule is common to all major families of P-loop NTPases. In enzyme-bound NTP molecules, the β- and γ-phosphates are in an almost perfectly eclipsed conformation due to interactions with Mg^2+^ and Lys^WA^ (see Figure 1D), in contrast to their staggered configuration in water [29,164]. The repulsion of the eclipsed oxygen atoms has the potential to destabilize the O^3B^–P^G^ bond [25,54,165,166]. 

Our finding that stimulators always interact with γ-phosphate indicates that this interaction may contribute further to destabilization of the O^3B^–P^G^ bond. For example, the twisted γ-phosphate may become more eclipsed relative to the α-phosphate, so that the oxygen atoms of the α- and γ-phosphate repel each other, as suggested by Rudack and colleagues [167]. Moreover, any twisting or pulling γ-phosphate can destabilize the entire Mg-NTP system, inevitably disturbing the coordination sphere of Mg^2+^, since the O^1G^ atom of γ-phosphate is one of the Mg^2+^ ligands (Figure 1D and Figure 2).

#### 4.2.3. Catalytic Factors in P-loop NTPases: Planarization of γ-phosphate 

Even in the absence of the stimulator, the oxygen atoms of the γ-phosphate are already “pulled up” to the β-phosphate by Lys^WA^, which interacts with O^1B^ and O^2G^, and by Mg^2+^, which interacts with O^2B^ and O^1G^; see Figure 1B and [168]. The stimulatory moiety in the AG site further planarizes γ-phosphate by drawing the O^3G^ atom toward O^2A^ and enabling the interaction between **HN**^K-3^ and O^2G^ (Figure 1, Figure 2, Figure 3, Figure 4, Figure 5 and Figure 6, Table S1). Indeed, in the presence of stimulatory moieties—for example in the pre-TS structures shown in Figure 4 of the companion article [30]—the O^3B^-P^G^-O^1G^, O^3B^-P^G^-O^2G^, and O^3B^-P^G^-O^3G^ angles are mostly less than the 109° expected for an ideal tetrahedron; see also [168].

Even when interacting only with γ-phosphate, the stimulator is often located between α- and γ-phosphates, as in dynamins (Figure 3A) or ABC transporters (Figure 5D) and can planarize γ-phosphate by tying O^3G^ to the “head” of the NTP molecule. For instance, the Na^+^ ion in dynamins, while not reaching the α-phosphate directly, is connected to it via two noncovalent bonds (Figure 3A). The signature motif of ABC-NTPases is H-bonded via conserved Ser and Gln residues to the O2′ atom of the ribose (Figure 5D).

Notably, the twisted or tilted γ-phosphate becomes more eclipsed relative to α-phosphate but *less* eclipsed relative to β-phosphate [29], which might be a prerequisite for inversion of the γ-phosphate group (otherwise, in the case of an ideal eclipse, the oxygen atoms of β-phosphate would prevent the inversion of γ-phosphate). 

#### 4.2.4. Catalytic Factors in P-loop NTPases: Electrostatic Compensation of the Negative Charge of the Phosphate Groups

The positive charges of amino group of Lys^WA^, the Mg^2+^ ion, and several **HN** groups of the P-loop compensate for the negative charges of phosphate oxygen atoms (see Figure 2B) so that electrons are “pulled away” from the P^G^ atom; see for example [25,62,159,160,161,163,167]. Notably, the phosphate chain “sits” on the last N-terminal turn of the α_1_-helix, which generally carries a dipole positive charge of about 0.5 [25,169]. This positive charge should also contribute to electrostatic compensation. 

The positions of the groups involved are strictly conserved (Figure 2), so that such a compensation is common to all major families of P-loop NTPases. This may, at least partly, explain why the binding of the GTP molecule to the catalytic site of Ras GTPase accelerated hydrolysis by five orders of magnitude, as compared to hydrolysis in water, even in the absence of an activating partner [28,170]. 

In addition to these common positively charged moieties, class-specific auxiliary residues could be involved, as, for instance, “sensors 3” in some AAA+ ATPases. On activation, the negative charges of the γ-phosphate oxygen atoms are additionally compensated by the positive charges provided by most stimulators; see Figure 1, Figure 2, Figure 3, Figure 4, Figure 5 and Figure 6 and the companion article [30]. The additional electrostatic compensation significantly increases the electrophilicity of the P^G^ atom. 

Rudack and colleagues showed in their QM/MM calculations that the insertion of the arginine finger alone increases the partial positive charge on P^G^ to 1.46 elementary charges [167]. The interaction of O^2G^ with **HN**^K-3^, as described in the companion article [30] and in [29] should further increase the positive charge on P^G^ in the pre-transition state.

In Supplementary File S3, we argue that the electrostatic potential at the catalytic sites of diverse P-loop NTPases is distributed unevenly, which has already been noted for particular enzymes of this family; see, e.g., [171]. Notably, the local electric field is directed approximately from the positively charged cluster around the P-loop to the negatively charged cluster around Asp^WB^. Consequently, in those cases where the stimulator is positively charged, its positive charge not only secures the bonding with particular oxygen atom(s) of triphosphate and increases the positive charge on P^G^ but additionally polarizes the whole catalytic pocket. 

#### 4.2.5. Catalytic Factors in P-loop NTPases: Does the [Ser/Thr]^K+1^–Asp^WB^ Pair Accept a Proton from W_cat_?

In their comprehensive review on enzymatic mechanisms of phosphate transfer, Cleland and Hengge wrote about myosin: “The problem for an ATPase … is thus to position a water molecule so that it *is* in a position to attack the γ-phosphorus. This requires steric restraints as well as organized hydrogen bonding networks. More specifically, there must be a path for one proton of the attacking water molecule to reach a suitable acceptor” (quoted from [52]). 

In P-loop NTPases, the proton from W_cat_ is commonly thought to be taken up either by γ-phosphate in TRAFAC class NTPases [57,131] or by W_cat_-coordinating, “catalytic” Glu or Asp residues in other classes of P-loop NTPases [8,43,97,100,116,117,125,130,172,173,174]. Still, our comparative structure analysis showed that such glutamate or aspartate residues, present in all classes of NTPases except the TRAFAC class, are non-homologous. 

Indeed, in SIMIBI NTPases, W_cat_-coordinating Glu or Asp residues are at the C-tip of their WB+1 β-strands (Figure 4A,B). In most classes of ASCE ATPases, the “catalytic” Glu^D+1^ follows Asp^WB^; see Figure 5 and [14,16,24]. In F_1_/RecA ATPases, the “catalytic” Glu residue is located at the C-tip of the WB+1 β-strand; see Figure 6 and [8,14,19]. Those kinases that require deprotonation of the second substrate have usually an Asp^WB^-Leu^D+1^-[Asp/Glu]^D+2^ motif where the D+2 residue interacts with the prospective nucleophilic group; see Figure 4D and sequence alignments in [9]. Such an absence of homology is unusual for catalytically critical residues.

More importantly, neither γ-phosphate nor W_cat_-coordinating glutamate or aspartate residues can hold the proton from W_cat_. Indeed, the inverse solvent isotope effect on the GAP-activated hydrolysis by the Ras GTPase, as measured upon H/D substitution [175], indicates that the deprotonation is likely to occur before the rate-limiting step of bond cleavage [52]. The bond cleavage time is in the millisecond range (100 ms at 260°K in a Ras GTPase activated by the RasGAP [176]). During this time, the proton must be “detained” in such a way as to prevent its return to OH^–^_cat_ and the reversal of the reaction. In general, the ability to keep the proton is seen as a prerequisite for the completion of enzymic reactions with participation of deprotonated nucleophiles, as discussed e.g., for the eukaryotic cAMP-dependent protein kinase [177,178]. 

The common belief of proton trapping from water (pK_a_ = 14.0) either by a “catalytic” glutamate or aspartate residue in a polar environment (with expected pK_a_ in the range of 2.0–6.0) or by γ-phosphate (with pK < 3.0 [179,180]) is at odds with the basic rules of proton transfer as formulated by Eigen [181,182]. 

After Eigen, if the donor and acceptor of the proton are connected by a “hydrogen bridge”, proton transfer between them is “practically unhindered *provided the difference pK_acceptor_—pK_donor_ is positive*” (quoted from [182]). For the interaction of W_cat_ with γ-phosphate or W_cat_-coordinating Glu/Asp residues, the respective difference is strongly negative so the proton will promptly (in picoseconds) return to W_cat_.

It was speculated that the W_cat_-coordinating groups may facilitate its catalytic deprotonation by decreasing the proton affinity of W_cat_; see, e.g., [183]. Our structural analysis of TS-like structures showed that positively charged side chains of Lys or Arg, which could significantly lower the pK value of W_cat_, interact with it only in a few classes of P-loop NTPases, e.g., in some AAA+ ATPases; see Section 3.2.2 and Table 1. Even in these cases, their positive charges can hardly decrease the apparent pK of W_cat_ by >10 pH units, which is needed for sustainable protonation of a “catalytic” Glu/Asp residue or γ-phosphate. Hence, neither “catalytic” acidic residues nor γ-phosphate can trap a proton from W_cat_ per se. 

As early as in 2004, some of these problems were recognized by Frick and colleagues [184,185] who calculated the pK values for ionizable residues of the SF2 class Hepatitis C virus (HCV) NS3 helicase by using the MCCE software [186]. Frick and colleagues wrote about supposedly W_cat_-coordinating Glu291^D+1^ and its preceding Asp290^WB^: “to function as a catalytic base, the pKa of Glu291 would need to be much higher than that of a typical Glu in a protein. However, electrostatic analysis of all HCV helicase structures reveals that neither Glu291, nor any nearby Glu, has an abnormally high pKa. 

In contrast, Asp290 has a pKa as high as 10 in some structures and as low as 3 in others. Interestingly, in structures in the open conformation (such as 8OHM), the pKa of Asp290 is low, and in the closed conformation (e.g., 1A1V), the pKa of Asp290 is higher than 7, suggesting that Asp290 picks up a proton (like a catalytic base) when the protein changes from the open to the closed conformation. Thus, Asp290 could serve as a catalytic base…” (quoted from [185]). 

Frick and colleagues made their calculations with crystal structures of helicases that contained neither Mg-ATP nor its analogues. Therefore, they were unaware of the exact positions of Asp290^D^ and Glu291^D+1^ relative to the bound substrate and could only guess by analogy with known structures of related P-loop NTPases. The two subsequently resolved ADP:AlF_4_^–^-containing, TS-like crystal structures of HCV NS3 helicase (PDB IDs 3KQL [187] and 5E4F [118]) show a typical for SF2 helicases arrangement of Asp290^WB^ and Glu291^D+1^ (see Figure 5C). Furthermore, both structures show short 2.4 Å H-bonds between Ser211^K+1^ and Asp290^WB^ as seen in Figure 5C. 

Unlike “catalytic”, W_cat_-coordinating glutamate or aspartate residues, Asp^WB^ is strictly conserved in P-loop NTPases. Equally strictly conserved is the [Ser/Thr]^K+1^ residue [9,10,12,188]. The replacement of any of these residues leads to the loss of activity; see, e.g., [185,189,190,191,192,193,194,195,196].

Both Asp^WB^ and [Ser/Thr]^K+1^ residues can translocate protons. Furthermore, they are the only strictly conserved residues of the Walker A and Walker B motifs that can do that. Therefore, building on our comparative structure analysis and following Frick and colleagues who suggested Asp290^WB^ as a catalytic base in the HCV NH3 helicase [184,185], we propose here that *the buried, H-bonded pair of strictly conserved [Ser/Thr]^K+1^ and Asp^WB^ residues serves as a universal catalytic module in P-loop NTPases of all classes. It accepts a proton from W_cat_ and holds it as long as needed.*

It can be countered that the [Ser/Thr]^K+1^–Asp^WB^ pair does not interact directly with W_cat_. However, direct tracing of intra-protein displacements of protons in energy converting enzymes and chemical models (see [181,182,197,198,199,200,201,202,203,204,205,206,207,208,209] for reviews) showed that fast proton transfer over a distance of up to 20 Å can be mediated by water bridges, provided that the distance between the groups involved is ≤3.0 Å, in accordance with Eigen [182]. Thereby it does not matter that water molecules are equally poor proton acceptors (with pK_b_ of 0.0) and proton donors (with pK_a_ of 14.0) because protons pass water by the so-called von Grotthuss mechanism [210,211,212]. 

According to the modern version of this relay mechanism, the bridging molecule receives an external proton simultaneously with the transfer of its own proton to the next carrier at picoseconds [211,212]. Apart from water molecules, the side chains of serine, threonine, tyrosine, cysteine, and neutral histidine are suitable for proton transfer by the von Grotthuss mechanism: they have a proton-accepting lone pair of electrons and also their own proton to transfer on [213]. In addition, protons are transferred fast between carboxyl groups when they are bridged by single water molecules [181,182,203,207]. Zundel and colleagues showed that such systems are highly polarized [197,214], which lowers the activation barriers for proton transfer. 

**Figure 9:**
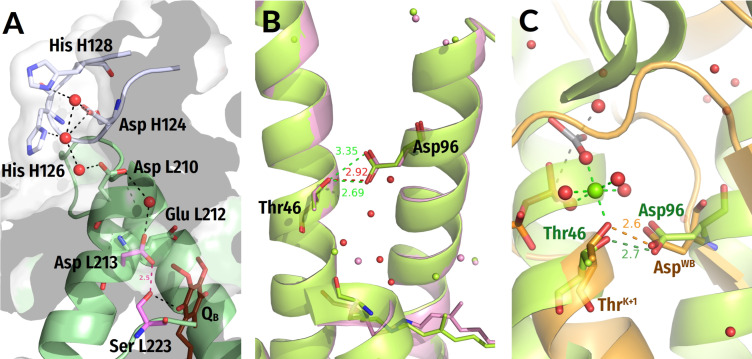
Proton traps in the photochemical reaction center and bacteriorhodopsin as compared with the Thr^K+1^-Asp^WB^ pair of P-loop NTPase. Distances are given in ångströms. (**A**) A high-pK proton trap in the photosynthetic reaction center (PRC) from *Rhodobacter sphaeroides.* Shown is the PRC in the charge-separated state (PDB ID 1DV3 [215]). Here, a proton path connects the protein surface with the buried binding site of the secondary ubiquinone acceptor Q_B_. The proton is translocated over more than 20 Å via amino acid residues of the two PRC subunits, L and H. The proton pathway begins at the two His residues on the surface (His-H126 and His-H128); they are thought to have pK values of about 7.0 and to harvest protons that are ejected onto the photosynthetic membrane surface by the ATP synthase. From these histidine residues, the proton goes, via water-bridged carboxyl groups of Asp H-124 and Asp-L210, to the Q_B_ -binding pocket. Proton transfer from the surface to the more hydrophobic interior of the protein becomes possible after the Q_B_ molecule docks to the Glu-L212–Asp-L213–Ser-L223 cluster [216] and turns it into a buried, high-affinity proton acceptor (a *membrane proton trap* according to Mitchell [217]), with a functional pK of about 9.5–10.0 [218,219,220,221,222]. This high functional pK is apparently due to the expulsion of water upon Q_B_ binding, the negative charge on the Glu-L212–Asp-L213 pair, and the H-bond between Asp-L213 and Ser-L223, through which the proton passes to Q_B_. The functional pK value of the trap increases to > 12.0 after the appearance of an electron on Q_B_ and the formation of the Q_B_^−·^ anion radical, which attracts further protons into the buried catalytic site [202]. Mutations of either Glu-L212, or Asp-L213, or Ser-L223 block proton transfer from the surface [218,223,224]. For further details, see [206,215,225,226,227,228]. (**B**) A high-pK proton trap in bacteriorhodopsin (BR), a membrane protein that pumps a proton across the membrane in response to the photoexcitation of its retinal pigment; see [199,200,229,230] for reviews. Shown is the superposition of femtosecond X-ray laser-captured structures of bacteriorhodopsin from [231] in a closed resting state (PDB ID 6G7H, light-green) and in an “open” state 8.3 ms after illumination (PDB ID 6G7L, pink). Additional red-coloured water molecules were taken from a high-resolution crystal structure of the V49A bacteriorhodopsin mutant that was crystalized in an “open” state (PDB ID 1P8U, [232]). In the BR, the key proton carrier Asp-96 has pK of ~12.0 owing to the absence of water in the vicinity and a H-bond with Thr-46 of the nearby α-helix. Replacement of Thr46 by a valine decreased the apparent pK of Asp96 by approximately two pH units [233], which may characterize the contribution of the H-bond between Thr46 and Asp96 to the unusually high apparent pK of the latter. The photoisomerization of the retinal twists the α-helices and allows water molecules in the space between them; see the pink structure [199,208,209,234,235,236,237,238]. The pK value of Asp-96 shifts to 7.1, which allows its deprotonation and proton transfer, via a transiently formed water chain, to the Schiff base of retinal at about 10 Å; see [239,240]. Later, the cleft between helices closes again, the pK of Asp-96 returns to 12.0, and it is re-protonated from the surface [199,200,230,235]. (**C**) The Thr46-Asp96 pair of BR it its resting closed state (the green structure from panel 10B, PDB ID 6G7H [231]) superimposed with the Thr^K+1^—Asp^WB^ pair from the transition-state-like, VO_4_^-^-containing structure of myosin shown in Figure 3D, orange (PDB ID 1VOM [93]).

The best-studied examples of such proton-conducting chains are known from the photochemical reaction center (PRC) of α-proteobacterium *Rhodobacter sphaeroides* (as shown in Figure 9A and described in its caption) and bacteriorhodopsin of an archaeon *Halobacterium salinarum* (as shown in Figure 9B and described in its caption). 

The two proton transfer routes shown in Figure 9A,B were reconstructed from direct electrometric tracking of flash-induced proton displacements [199,241,242,243,244,245]; kinetic IR, and UV/Vis spectroscopy data [199,200,220,221,225,239,242,246]; kinetic ESR measurements [229,234,236,247]; and comparative structure analyses [208,216,227,235]. These are the two best-understood cases of intra-protein proton transfer by far. 

In the case of PRC (Figure 9A), the buried proton acceptor must have a functional pK value greater than 9.0 to compensate for the desolvation penalty of about three pH units, which must be “paid” for delivery of a proton from a pK-neutral “antenna” group at the protein surface into the hydrophobic membrane [248,249]. However, if such a buried proton acceptor is equilibrated with the protein surface, its pK value can hardly exceed the ambient pH, which is usually neutral, because the groups with even higher pK values are already protonated. Hence, the strong proton acceptor shown in Figure 9A is not fully equilibrated with the surrounding solution. In this case, it is better to speak about the *apparent/functional* pK or high *proton affinity*. Such strong proton acceptors usually emerge only transiently [222,250].

The proton traps in Figure 9A,B include H-bonded [Ser/Thr]—Asp pairs (modules). These examples show that proper H-bonding of a protein-buried Asp residue with a nearby Ser/Thr residue in a hydrophobic environment can generate a trap with a high proton affinity; such traps can, in principle, accept protons even from water. Specifically, in the absence of pH-buffers and protons from the ATP synthase, the protons that pass through the PRC to compensate the negative charge at the Q_B_ site (Figure 9A) appear to detach from surface water molecules, presumably polarized by surface charges [225]. 

**Figure 11:**
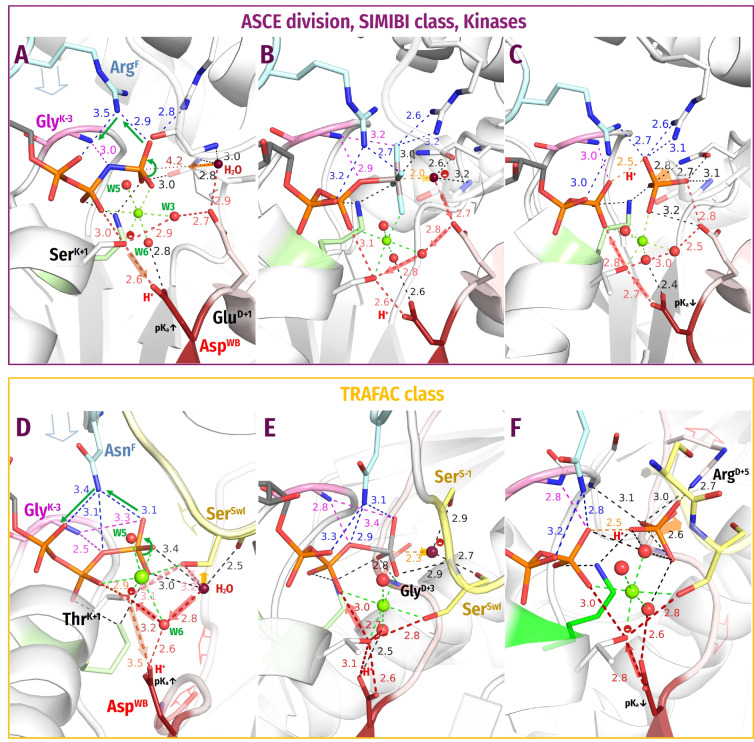
**Two tentative mechanisms of proton transfer from W_cat_ to Asp^WB^ in P-loop NTPases.** The shade of the red arrows of proton transfers varies through panels (**A**–**F**) to emphasize that the kinetic coupling of these steps with each other and reactions in the catalytic site could vary depending on the thermodynamics of particular enzymes. Other colours as in Figure 3, Figure 4, Figure 5 and Figure 6. Top (**A**–**C**): Tentative mechanism of proton transfer via the W_cat_-coordinating Glu/Asp residue in all classes of P-loop NTPases but TRAFAC class enzymes, as illustrated by structures of the SF1 helicase Pif1. (**A**) In the pre-catalytic state, which is exemplified by a structure with a bound non-hydrolyzable ATP analogue ANP (PDB ID 6HPH [263]), the binding of ATP and closure of the catalytic site increases the proton affinity of Asp^WB^. Activation-induced constriction of the catalytic site and twist of γ-phosphate prompt the proton redistribution from [Ser/Thr]^K+1^ to Asp^WB^. (**B**) In the TS, which is exemplified by a structure with a bound TS analogue ADP:AlF_4_^–^ (PDB ID 5O6B [174]), a proton from W_cat_ fills the vacancy at the anionic [Ser/Thr]^K+1^ via the W_cat_-coordinating Glu^D+1^ and W3; the resulting **OH^—^_cat_** attacks γ-phosphate. (**C**) in the post-TS, which is exemplified by a structure with ADP:MgF_4_^2-^ bound (PDB ID 6S3I [264]), where we replaced the H_2_PO_4_^2-^ mimic MgF_4_^2−^ by H_2_PO_4_^2-^, the proton goes from Asp^WB^, via Ser^K+1^, to β-phosphate to compensate its negative charge (see the main text). Bottom (**D**–**F**): Tentative mechanism of proton transfer from W_cat_ to Asp^WB^ in TRAFAC class NTPases, as illustrated by structures of myosin. (**D**) For the pre-catalytic state, we used the ATP-soaked crystal structure of myosin II (PDB ID 1FMW [140]) with the Switch I loop taken from the structure of Myosin V complexed with ADP-BeF_3_ (PDB ID 1W7J [265]) and superimposed via Lys^WA^ and Mg^2+^. In this state, the binding of ATP and closing of the catalytic site increases the proton affinity of Asp^WB^. Constriction of the catalytic site upon activation and the twist of γ-phosphate prompt the proton redistribution from Thr^K+1^ to Asp^WB^ (purple arrow). The anionic Thr^K+1^ accepts a proton from W6 that, in turn, takes a proton from W_12_ (red arrows, cf. Figure 7C,D); alternatively, the proton route from W_12_ to Thr^K+1^ may involve the conserved Ser^Sw1^ (dashed red arrows) The resulting OH^−^_cat_ is brought into the apical position by the **CO** group of Ser^SwI^ (coloured yellow) and the WB-crest; (**E**), in the transition state, which is exemplified by a structure with a bound TS analogue ADP:VO_4_^–^ (PDB ID 1VOM [93]), OH^—^_cat_ attacks P^G^. In this constricted transition state, the protonic connection between OH^—^_cat_ and the Mg^2+^-coordinating water molecules appears to be broken. In the subsequent stage of the catalytic transition, the proton goes from [S/T]^WA^ to β-phosphate and compensates for the large negative charge that builds up upon the detachment of γ-phosphate (the red arrow). (**F**) In the post-transition state, which is exemplified by a structure with bound ADP and H_2_PO_4_^2^^−^ (PDB ID 4PJK [41]), the negative charge on β-phosphate is compensated by a hydrogen bond between β- and γ-phosphate. Asp^WB^ gives the proton back to Thr^K+1^ concomitantly with the opening of the catalytic pocket (see also the main text).

Our suggestion that a similarly H-bonded [Ser/Thr]^K+1^–Asp^WB^ pair accepts the proton from W_cat_ in P-loop NTPases challenges the current ideas on catalytic proton transfer in enzymes of this superfamily. Therefore, we further substantiate the suggested mechanism below (see also Figure 10 and Figure 11):

(1) We discovered that the “catalytic” Glu/Asp residues connect W_cat_ with ligands of Mg^2+^ in positions #3 or #6 in TS-like structures of P-loop NTPases of various classes (except the TRAFAC class); see Figure 1F, Figure 4, Figure 5, Figure 6 and Figure 7A. Notably, the six ligands of Mg^2+^ form a regular octahedron with edges 2.9–3.0 Å long, so that the ligands #3 and #6 are on a H^+^-transfer distance from [Ser/Thr]^K+1^ (Figure 10). The short H-bond between [Ser/Thr]^K+1^ and Asp^WB^ completes the proton-conducting pathway that connects W_cat_ with Asp^WB^ in all families of P-loop NTPases except the TRAFAC class. The proton pathways from W_cat_ to Asp^WB^, which resemble proton translocation systems of PRC and BR (cf Figure 9), are shown by the red dashed lines for P-loop NTPases of different classes in Figure 4, Figure 5, Figure 6 and Figure 7 and by dashed arrows in Figure 10.
(2) Mutations of Asp^WB^ to Asn, while retarding dramatically the activated hydrolysis, had no effect on the NTP binding [185,189,190,191,192,193]. In the case of *E. coli* F_1_-ATPase, the mutation even increased the affinity for ATP [191]. The Asp^WB^ to Asn mutation mimics the charge state of a protonated Asp^WB^. Hence, the protonation of Asp^WB^ is unlikely to distort the catalytic pocket and be the cause of universal catalytic incompetence of the Asp^WB^ to Asn mutants. We attribute this incompetence to the inability of Asn^WB^ to trap a proton from [Ser/Thr]^K+1^.
  **Figure 10:**
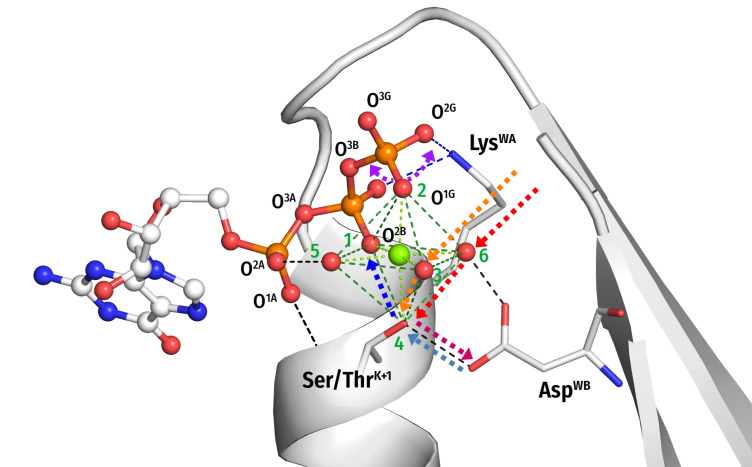
**Schematic presentation of tentative proton routes along the edges of the octahedral coordination shell of Mg^2+^ ion**. The Mg^2+^ ligand #3 is assumed to be a water molecule. Proton entry points via ligand #6 or ligand #3 are shown with red and orange arrows, respectively. Protonic connection between [S/T]^K+1^ and Asp^WB^ are shown by magenta and light blue arrows, the route from [S/T]^K+1^ to O^2B^ of β-phosphate is shown as a dark blue arrow. The movement of the O^1G^ atom as a result of γ-phosphate twist is shown by purple arrows.
(3) The pKa of an aspartate residue in water is about 4.0, much lower than that of water (14.0). However, unlike the “catalytic” Glu/Asp residues or γ-phosphate surrounded by charged residues (Figure 1E,F, Figure 3, Figure 4, Figure 5 and Figure 6), Asp^WB^ is in a nonpolar environment and its functional pK is likely to be high when the catalytic site is closed. Asp^WB^ is in the middle of an αβα sandwich, on the interface between the β-pleated sheet and the α_1_-helix; such interfaces are stabilized by hydrophobic interactions [169]. In addition, Asp^WB^ is preceded by four hydrophobic residues of the Walker B motif (Figure 1); the adjacent β strands, as well as the α_1_-helix, also contain many hydrophobic residues; see the sequence alignments in [9,10,12,188].
  Our data show that the relative SASA of Asp^WB^ drops below 6% in the presence of TS-analogues (Figure 8B). Upon constriction of the catalytic pocket and expulsion of eventually present water molecules, the hydrophobic environment should elevate the proton affinity of the H-bonded Asp^WB^, as it happens with similarly H-bonded Asp96, which has a functional pKa of ~12.0 in a hydrophobic environment of the ground-state BR. Figure 9C shows that the structure of the Ser186^K+1^–Asp454^WB^ pair of myosin overlaps nicely with the Thr46–Asp96 pair of BR.
(4) Unfortunately, we are not aware of non-commercial software for reliable calculating the absolute pKa values in proteins. Hence, we used the PROPKA web server at https://www.ddl.unimi.it/vegaol/propka.htm (accessed on 14 August 2022). The server uses the PROPKA v. 2.0 half-empiric software that allows to assess pKa changes in response to ligand binding in the catalytic center [66].
  We applied PROPKA to the bovine ATP synthase, an extremely well-studied enzyme with a plethora of structures available [251]. The three catalytic centers of this ring-shaped enzyme work in turn, according to the so-called binding change mechanism [252]. Therefore, the catalytic pockets are usually open to varying extents. Using mixtures of nucleotides and their analogues, Walker and co-workers managed to obtain several structures where different centers in the same structure are as if in different stages of the catalytic cycle [253,254,255].
  We calculated the changes in pKa values of Asp256^WB^ and the catalytic Glu188 (see Figure 6A) in response to opening/closing of the catalytic pockets and in the presence of different ligands. The values given in Supplementary Table S3 show that the estimated pKa of Asp256^WB^ varies from around 9.0 (closed or constricted site with a nucleotide bound) to around 2.5 (empty site or a site with Pi bound). Interestingly, an intermediate pKa value of around 5.5 was obtained for a site containing ADP and Pi, which is believed to be half-opened [43]. Hence, the pKa of Asp^WB^ can increase by 7 units upon closing of the catalytic pocket. The estimated pKa values of Glu188 are much lower, usually around 5.0 when a nucleotide is bound (Table S3).
(5) In contrast, the pK of [Ser/Thr]^K+1^, which is about 13.0 in water, is likely to be reduced when [Ser/Thr]^K+1^ serves as a Mg^2+^ ligand. Coordination of a Zn^2+^ ion by a serine side chain is known to decrease the pK value of the latter up to 5.5 yielding a serine anion (alkoxide) at neutral pH; see [52] and the references therein. The impact of a Mg^2+^ ion should be weaker; still, within a closed/constricted catalytic site, the low dielectric permittivity would enhance electrostatic interactions. [Ser/Thr]^K+1^ is the most deeply buried of the Mg^2+^ ligands (see Figure 1, Figure 2, Figure 3, Figure 4, Figure 5, Figure 6 and Figure 10), and thus it should be the most sensitive to electrostatic effects. As a result, the functional pK value of [Ser/Thr]^K+1^ may dramatically decrease upon constriction of the catalytic site.
(6) The shortening of H-bonds found in the TS-like structures (Figure 8A) enables estimation of the difference in functional pK values of [Ser/Thr]^K+1^ and Asp^WB^ in the TS. It is well established, based on ample experimental evidence, that hydrogen bonds “generally shorten as ΔpKa, the difference in the donor and acceptor pKa values, decreases” (quoted from [142]). Specifically, Herschlag and colleagues observed, on various systems, that the Δp*K*a decreases linearly from 20 to 0 with the decrease in the O—H••••O distance from 2.9 Å to 2.4 Å, with a slope of 0.02 Å/p*K*a unit, [142,256]. In NDP:AlF_4_^-^-containing structures with constricted catalytic site, the length of the H-bonds between [Ser/Thr]^K+1^ and Asp^WB^ varies around 2.5 Å (Figure 1E,F, Figure 3, Figure 4A,B, Figure 5C,D, Figure 6A and Figure 8A), which corresponds to ΔpK < 3.0 and indicates a low-barrier hydrogen bond [142,256]. Hence, [Ser/Thr]^K+1^ and Asp^WB^ may have comparably high proton affinities in a constricted catalytic site.
(7) Limbach and colleagues combined low-temperature UV-Vis and ^1^H/^13^C NMR spectroscopy (UVNMR) to study the effect of solvent polarity on the proton equilibrium between phenols and carboxylic acids [257,258,259], which system can be viewed as a model of the [Ser/Thr]^K+1^—Asp^WB^ H-bonded pair. These authors have shown that proton relocates from the hydroxy group to the carboxyl with decrease in polarity.
(8) In the octahedral coordination shell of Mg^2+^, the O^1G^ atom of γ-phosphate is the ligand opposite to [Ser/Thr]^K+1^ (Figure 10). Therefore, the stimulator-induced rotation of γ-phosphate, by moving O^1G^ in *any* direction (as shown by dashed purple arrows in Figure 10), would inevitably increase the distance between O^1G^ and the hydroxyl of [Ser/Thr]^K+1^. Pulling away the negatively charged O^1G^ will increase the cumulative positive charge at [Ser/Thr]^K+1^ prompting the relocation of its proton to Asp^WB^, e.g., in response to a thermal fluctuation [259] (Figure 11A).
(9) We suggest that the resulting Mg^2+^-coordinated Ser/Thr anion (alkoxide), used as a proton acceptor from water by many enzymes [52,260,261,262], withdraws the proton from W_cat_ (or the sugar moiety in some kinases) via proton pathways shown in Figure 4, Figure 5, Figure 6, Figure 7A, Figure 10 and Figure 11B. This proton transfer should be additionally driven by strong local electric field (see Supplementary File S3 on the uneven electric field distribution in the catalytic sites of P-loop NTPases). The resulting state where both [Ser/Thr]^K+1^ and Asp^WB^ are protonated corresponds to the ground state of the Thr46-Asp96 pair in the BR (see the light-green structures in Figure 9B,C).
(10) The formed anionic nucleophile (e.g., OH^−^_cat_), while stabilized and polarized by its ligands, is attracted by the electrophilic P^G^ atom (Figure 11B). The proton affinity of the anionic nucleophile decreases as it gets closer to P^G^, so that proton return from the [Ser/Thr]^K+1^—Asp^WB^ couple becomes increasingly unfavorable, eventually satisfying the Eigen’s condition for proton transfer and making it complete.

In TS-like structures of TRAFAC class NTPases, the H-bond between [Ser/Thr]^K+1^ and Asp^WB^ is as short as in other classes of P-loop NTPases; see Figure 1F and Figure 3A–D, and Table S1. However, these NTPases have no “catalytic” W_cat_-coordinating Glu/Asp residues. Instead, they all have a strictly conserved [Thr/Ser]^SwI^ residue that coordinates Mg^2+^ by its side chain as ligand #3 and, at the same time, stabilizes W_cat_ with its **CO** group (Figure 1E, Figure 3 and Figure 7C,D). 

In this configuration, the side chain oxygen of [Ser/Thr] ^SwI^ is exactly 2.9 Å away from the side chain oxygen of [Ser/Thr]^K+1^, as they are neighbouring ligands in the octahedral coordination shell of Mg^2+^ (see Figure 11). In the absence of a bound TS-analogue, however, [Thr/Ser]^SwI^, as well as the whole Switch I loop, are highly mobile with water sporadically being involved as the Mg^2+^ ligand #3 instead of [Thr/Ser]^SwI^; see Figure 7B–D and Figure S5B and [137,266]. 

It is tempting to speculate that the side chain of mobile [Thr/Ser]^SwI^ may be involved in a von Grotthuss-type proton transfer from the would-be W_cat_ to [Ser/Thr]^K+1^ in the pre-TS state. Kumawat and colleagues, studying the small Rho GTPase by MD simulations, showed that seven water molecules, on average, come within 4 Å distance from Thr37^SwI^ in a closed, GTP bound state [267]. This amount should be sufficient to transfer a proton from the would-be W_cat_ to anionic [Ser/Thr]^SwI^ over a distance of only 4–5 Å concomitantly with the constriction of the catalytic site. 

Alternatively, a W_12_ or W_13_ water molecule can give its proton to [Ser/Thr]^SwI^ and then move by 1–2 Å into the apical position turning into OH^—^_cat_ as documented in Figure 7D. For myosin, where W_12_ is “visible” owing to extra bonds with residues of Switch I (Figure 7D), even two tentative/alternative proton route(s) are predictable as shown by solid and dashed red arrows in Figure 11D. Similar proton pathways can be proposed for Ras-like GTPases based on the “open” structure shown in Figure 7B. 

In TS-like structures of TRAFAC class NTPases, as already mentioned, [Ser/Thr]^SwI^ is fixed in a distinct position where it coordinates both the W_cat_ and Mg^2+^; here the distance from its hydroxyl group to W_cat_ is about 4.5 Å (Figure 1E, Figure 3A–D and Figure 7D), there appear to be no water molecules in between. It is tempting to speculate that this configuration may hamper the unwanted return of a proton from Asp^WB^ to OH^—^_cat_. 

#### 4.2.6. Catalytic Factors in P-loop NTPases: Charge Compensation at β-phosphate

According to Herschlag and colleagues [54,61], the activation barrier of NTP hydrolysis is due to the strong negative charge that develops at β-phosphate as γ-phosphate breaks away; this charge should be compensated upon catalysis. The post-TS structures of the RhoA/RhoA-GAP complex (PDB ID 6R3V [183]) and activated myosin (PDB IDs 4PFP, 4PJK [41]) with NDP and detached P_i_ (Figure 11F), as well as the structure of SF1 helicase Pif1 with a H_2_PO_4_^2^^−^ mimic ADP:MgF_4_^2^^−^ bound (Figure 11C) show that the negative charge of β-phosphate is compensated by the joint action of Mg^2+^, Lys^WA^, Arg/Lys/Asn fingers and **HN**^K-3^. 

At this stage, the H-bond between **HN**^K−3^ and O^2G^ is lost so that **HN**^K−3^ is fully involved in compensating for the negative charge at β-phosphate. In addition, the structures show a short H-bond of 2.4–2.5 Å between the oxygen atoms of β-phosphate and P_i_ (see Figure 11C,F and [41,183,264,268]); this H-bond apparently also compensates for the negative charge at β-phosphate. 

The proton for the H-bond between β-phosphate and P_i_ is thought to stem from W_cat_ [41,183,268] and should be somehow transferred to β-phosphate. In the octahedral coordination shell of Mg^2+^, [Ser/Thr]^K+1^ is 2.9 Å away from β-phosphate, so that the proton that came from W_cat_ can directly pass on and compensate the negative charge (as shown in Figure 10 and Figure 11C,E). The proton vacancy on [Ser/Thr]^K+1^ could be then refilled by a proton from Asp^WB^ (Figure 10 and Figure 11C,F), thus, restoring the initial protonic configuration. Since the octahedral arrangement of Mg^2+^ ligands (Figure 10) is similar in all P-loop NTPases, the proton can relocate from Asp^WB^ to β-phosphate, via [Ser/Thr]^K+1^ in all of them.

#### 4.2.7. Evidence for Transient Protonation of Asp^WB^ from Infrared Spectroscopy Data

In PRC and BR shown in Figure 9, the protonation of Asp/Glu residues was directly followed in the infrared (IR) spectral range [200,220,221,230,239,246,269]. In general, Asp and Glu residues are unique because the ν(C=O) vibration of their protonated carboxyl groups absorbs in the 1710–1760 cm^−1^ spectral region that is free from overlap with absorption of other protein components [270]. Specifically, the protonated Asp-96 of BR has an absorption maximum at 1741 cm^−1^ [239], whereas the protonated GluL212-AspL213-Ser223 complex of PRC absorbs at 1728–1725 cm^−1^ [220,221]. 

The application of the IR-spectroscopy to P-loop NTPases is complicated by the transient nature of Asp^WB^ protonation—proton passes through Asp^WB^ on the time scale of the NTPase turnover. Nevertheless, IR measurements in this spectral range were performed by Kim and colleagues who studied human Eg5, a kinesin-like motor protein of TRAFAC class, where Asp265^WB^ and Thr112^K+1^ are connected by a short H-bond of 2.59 Å (PDB ID 3HQD [271]). 

In their steady state experiments [272], Kim and colleagues investigated the action of monastrol, an allosteric inhibitor that binds some 12 Å away from the catalytic site [273] and increases the reversals in this enzyme by hampering the release of H_2_PO_4_^2−^ [274]. In the presence of monastrol, an absorption maximum at 1726–1722 cm^−1^ was recorded and attributed to the protonation of a carboxylic group [272]. These data are consistent with proton trapping at Asp265^WB^ when the overall equilibrium of ATP hydrolysis shifts to the left, as it happens in the presence of monastrol [274].

In kinetic experiments, the FTIR spectra of human Eg5 were monitored in real time and in response to the photorelease of caged ATP [275]. In the absence of microtubules, the hydrolysis by Eg5 proceeded at a time scale of seconds, which facilitated the IR measurements. A sharp absorption maximum at 1743 cm^−1^ appeared at approx. 3 s and then decayed, whereas H_2_PO_4_^2^^−^, as measured at 1049 cm^−1^, appeared at approximately 5 s and reached its maximum at 10 s (see Figure 1 in [275]). According to the views of the time, the authors attributed the maximum at 1743 nm to an “organized water cluster undergoing protonation” (quoted from [275]). 

This attribution, however, seems far-fetched. In proteins, protonated water clusters absorb at higher wave numbers of 1900–1800 cm^−1^ and show broad continuum bands [269]. It is also unlikely that a protonated water cluster with pK around zero could have a lifetime of seconds at neutral pH of the experiment. It is tempting to suggest that the well-defined, sharp transient maximum at 1743 cm^−1^ was in fact due to the transient protonation of Asp265^WB^ next to Thr112^K+1^ in human Eg5 (cf with the maximum at 1741 cm^−1^ of protonated Asp96, which is H-bound to Thr-46 in BR [239]). 

#### 4.2.8. Re-Assignment of Functions in and around the Walker A and Walker B Motifs 

In the framework proposed here, the Asp^WB^ of the Walker B motif serves as an ultimate trap for the proton from W_cat_, whereas the coordination shell of the Mg^2+^ ion functions as an octahedral proton transfer hub with almost all Mg^2+^ ligands involved (Figure 10). The proton is transferred from W_cat_ to Asp^WB^ in two steps: first, the proton shifts from [Ser/Thr]^K+1^ to Asp^WB^ in response to the constriction of the catalytic site and stimulatory interaction, and then a proton from W_cat_ fills the proton vacancy at [Ser/Thr]^K+1^, as shown in Figure 10. 

In sum, one proton relocates from W_cat_ to Asp^WB^. Still, the existence of separate proton transfer steps, as shown in Figure 10 and Figure 11 by different shades, provides flexibility; the mechanism could be adapted to the dissociative, associative, or concerted mechanism of hydrolysis. For instance, if the reaction is concerted, the proton from W_cat_ can pass directly through the Mg^2+^-coordinating ligands to β-phosphate, with the proton vacancy at [Ser/Thr]^K+1^ being filled by Asp^WB^ later, concomitantly with the loosening of the catalytic site and decrease in proton affinity of Asp^WB^, as shown in Figure 11D–F. 

The Asp^WB^ residue is almost strictly conserved throughout P-loop NTPases; see Table 1 and [9,10,12,24,105]. Only in several cases, its function is performed by Glu; see [24] for details. Some of such cases are shown in Figure S6 and described in its extended caption. The tentative interplay between Glu^WB^, Asp^E+1^ and Glu^E+4^ residues in Vir/PilT-like ATPases is considered separately in the Supplementary File S1 together with other ambiguous cases. 

Notably, the cause for the strict conservation of the entire Walker B motif has remained obscure thus far; to our best knowledge, no function has been attributed to the motif as a whole. The here suggested scheme invokes Asp^WB^ as the common terminal acceptor of a proton from W_cat_. Other four hydrophobic residues of the same motif serve as “hydrophobic protonic insulators”. They may be needed to increase the proton affinity of Asp^WB^ in the constricted catalytic site and to prevent eventual unwanted proton escape from Asp^WB^. 

In our model, [Ser/Thr]^K+1^ also acquires a new key function of a catalytic nucleophilic alkoxide, which may explain its strict conservation (see Table 1) and the loss of catalytic activity upon its replacement [196]. 

A few known exceptions are shown in Figure S7 and described in its extended caption. The most common outliers are glycine residues that substitute for [Ser/Thr]^K+1^ in several distinct families of P-loop nucleotide monophosphate kinases [9]; in these cases, a water molecule serves as the Mg^2+^ ligand #4; see Figure S7B. 

These multiple losses of [Ser/Thr]^K+1^, in fact, provide additional support for here proposed mechanism. It is in these P-loop kinases that the attacking nucleophile––the anionic phosphate moiety of a nucleotide monophosphate––needs no deprotonation and, therefore, does not need a catalytic alkoxide. Furthermore, the successful involvement of a water molecule as the fourth ligand of Mg^2+^ in these kinases indicates that the conservation of [Ser/Thr]^K+1^ in all other classes of P-loop NTPases may be related not to its function as a Mg^2+^ ligand but to its functioning as a catalytic alkoxide. 

As noted earlier, kinases do not interact with separate activator proteins; they are activated by binding the second substrate, which causes covering of the catalytic site by the LID domain and insertion of stimulatory finger(s) (Figure 4C,D and Figure S7B). Therefore, there is always some danger that the insertion of the finger(s) will stimulate a nucleophilic attack on γ-phosphate not by the anionic phosphate group of the second substrate but by a haphazard water molecule, leading to a futile ATP hydrolysis. Within our proposed scheme, an unwanted ATP hydrolysis can be prevented by replacing [Ser/Thr]^K+1^ with a residue incapable of accepting a proton from water. 

That is what independently occurred in several lineages of nucleotide monophosphate kinases; see Figure S7B and multiple alignments in [9]. In particular, a glycine residues are present in the K+1 position of human adenylate kinases except for adenylate kinase 6, which has a threonine residue [276]. It is for this kinase that both kinase and ATPase activities have been shown [277]. Hence, the consistent loss of [Ser/Thr]^K+1^–independently in several families of nucleotide monophosphate kinases [9]—finds a plausible explanation. These losses increase the specificity of nucleotide monophosphate kinases.

Neither of the exceptions in Figures S6 and S7 calls into question the suggested mechanism where the buried [Ser/Thr]^K+1^–[Asp/Glu]^WB^ module turns into a deep proton trap after the constriction of the catalytic site in most P-loop NTPases.

Why then the strictly conserved, H-bonded [Ser/Thr]^K+1^–Asp^WB^ pair has not established itself as a proton acceptor from W_cat_ thus far? The theory of catalysis by P-loop NTPases was developed initially and intensively for small GTPases of TRAFAC class, such as G_α_-proteins and Ras-like GTPases. Their W_cat_ molecules are stabilized not by a “catalytic” Glu/Asp but by **CO**^SwI^, Gly^D+3^ and Gln^D+4^ (Figure 1F and Figure 3C), so that Gln^D+4^ was initially suggested as the catalytic base. 

Warshel and colleagues challenged this view arguing that a Gln residue with pK < −2.0 is unlikely to accept a proton from water [57]. Among possible alternatives, they considered Asp57^WB^ of Ras GTPase but discarded it because its mutation did not affect the slow “intrinsic” activity of the lone GTPase. Instead, the γ-phosphate group was proposed as a universal proton acceptor for W_cat_ [57] and has been considered as such ever since in the TRAFAC class NTPases. 

Although, only a year later, it had been shown that mutations of Asp^WB^ slowed by orders of magnitude the fast activated hydrolysis by Ras/RasGAP complexes [193], the role of γ-phosphate as the proton acceptor was not revisited. After Cleland and Hengge noted that the direct proton transfer from W_cat_ to γ-phosphate is not possible “as the geometry does not permit such a four center reaction” [52], the involvement of additional water molecules between W_cat_ and γ-phosphate was hypothesized for TRAFAC class NTPases. 

In the case of other NTPase classes, the W_cat_-coordinating Glu (Asp) residues were considered as “catalytic” bases. Unfortunately, the seminal work by Frick and colleagues on the suitability of buried Asp^WB^ as a proton acceptor from W_cat_ [184,185], mentioned above, has not changed the common view. The inability either of γ-phosphate or “catalytic” Glu/Asp residues to serve as proton traps because of their low pK values has been overlooked. 

Our suggestion that the proton affinity of Asp^WB^ depends on the length of its H-bond with [Ser/Thr]^K+1^ explains why mutations of Asp57^WB^ affect the fast RasGAP-activated GTP hydrolysis [193] but have no impact on the slow intrinsic hydrolysis by lone Ras GTPases [57]. In lone GTPases, the difference between the time of proton equilibration over the whole protein (microseconds) and the catalysis time (minutes/hours) is so large that the mere presence of a dedicated proton acceptor for W_cat_ is unlikely; see also [54]. 

Since the catalytic site is fully exposed in the absence of a GAP and its closing is not possible, W_cat_ eventually loses a proton to the neutral bulk solution. Analogously, some group with a pK value slightly higher than pH provides a proton for β-phosphate and then is repleted from the solution. The terminal acceptor/donor of the proton is the neutral bulk solution so that the overall reaction is slow (as discussed elsewhere in relation to proton transfer in PRC [225]). 

Hence, the intrinsic hydrolysis in the absence of the GAP is insensitive to mutations of Asp^WB^ because this residue is unlikely to be specifically involved. In the exposed catalytic site of a lone Ras GTPase, the pK value of Asp^WB^ is expected to be ≤5.0 (which corresponds to the 2.6–2.7 Å length of the [Ser/Thr]^K+1^–Asp^WB^ H-bond in the structures that contain NTPs or their analogues; see Figure 8 and [142]), so that Ser17^K+1^ stays protonated (see the neutron crystal structure of Ras, PDB ID 4RSG, [278]) and cannot accept a proton from W_cat_. Such a mechanism of slow, solution-controlled hydrolysis may also operate, even in the presence of an activator, in those P-loop NTPases where Asp^WB^ and/or [Ser/Thr]^K+1^ were mutated [185,189,190,191,192,193,194,195,196]. 

In a RasGAP-stimulated wild-type complex, the situation differs. The constriction of the catalytic site and shortening of the H-bond with Ser17^K+1^ up to 2.4–2.5 Å would increase the proton affinity of Asp57^WB^ (see Figure 8 and [142]), turning it into a deep trap for a proton from Ser17^K+1^. The anionic alkoxide of Ser17^K+1^ is thermodynamically much more favourable proton acceptor from W_cat_ than the pH-neutral solution. The same proton passes by the Grotthuss relay to β-phosphate when the latter is ready to accept it; there is no need to wait for a proton from the solution. As a result, hydrolysis is 10^5^ times faster than in the case of the lone Ras GTPase. Expectedly, the Asp57^WB^ to Asn mutation slowed the rate of the activated hydrolysis by Ras/RasGAP to the “intrinsic” level of hydrolysis observed in the absence of GAP [193]. 

In no way do we state that γ-phosphate is banned for the “catalytic” proton from W_cat_. Distance measurements indicate that the catalytic proton can pass to [Ser/Thr]^K+1^ and Asp^WB^ also through the oxygen atom(s) of γ-phosphate in NTPases of all classes, which, to some extent, justifies numerous QM/MM models of water-mediated proton transfers from W_cat_ to γ-phosphate. These proton routes, however, involve “uphill” proton transfer from water to enzyme-bound triphosphate moiety with pK < 3.0 [179,180]. Consequently, these routes are thermodynamically unfavourable and, therefore, slow. Unfortunately, the QM modelling does not capture thermodynamic obstacles of this kind. It is this hindrance that may have prompted repeated independent emergence of γ-phosphate-bypassing proton pathways from W_cat_ to the [Ser/Thr]^K+1^–Asp^WB^ pair, which we identified in TS-like structures of diverse P-loop NTPases; see Figure 3, Figure 4, Figure 5, Figure 6, Figure 7, Figure 10 and Figure 11. 

We turned towards the strictly conserved Asp^WB^ and [Ser/Thr]^K+1^ as proton acceptors from W_cat_ after we realized that the “catalytic” Glu/Asp residues are provided by topologically diverse protein segments in different classes of P-loop NTPases; see Figure 1F, Figure 4, Figure 5, Figure 6, Figure 7A, Table 1, Section 3.2, and [9,10,24,279]. Our analysis shows that proton pathways via “catalytic” Asp or Glu residues, emerged independently in kinases, SIMIBI NTPases, “medium-size” ASCE ATPases, and large F_1_/RecA-like ATPases, i.e., four times, at least. 

This observation was unusual since catalytic bases are usually conserved within enzyme families. At the same time, the discovered connections between W_cat_ and the coordination shell of Mg^2+^, although involving non-homologous Glu/Asp residues, strongly resembled each other, as well as the proton path in the PRC (cf Figure 4, Figure 5 and Figure 6 and Figure 7A with Figure 9A), which suggested their functioning as proton pathways. 

In the scheme proposed here, these non-homologous, “catalytic” Asp/Glu residues, as well as, likely, [Ser/Thr]^SwI^ of TRAFAC class NTPases, are regarded as intermediate proton carriers, whereas thermodynamics of catalysis are determined by the difference in proton affinities between W_cat_ (or its analogues in kinases) and somewhat distantly located, but evolutionary conserved [Ser/Thr]^K+1^–Asp^WB^ module, which serves as a *catalytic base.*

#### 4.2.9. Minimal Mechanistic Model of NTP Hydrolysis by P-loop NTPases

**Figure 12:**
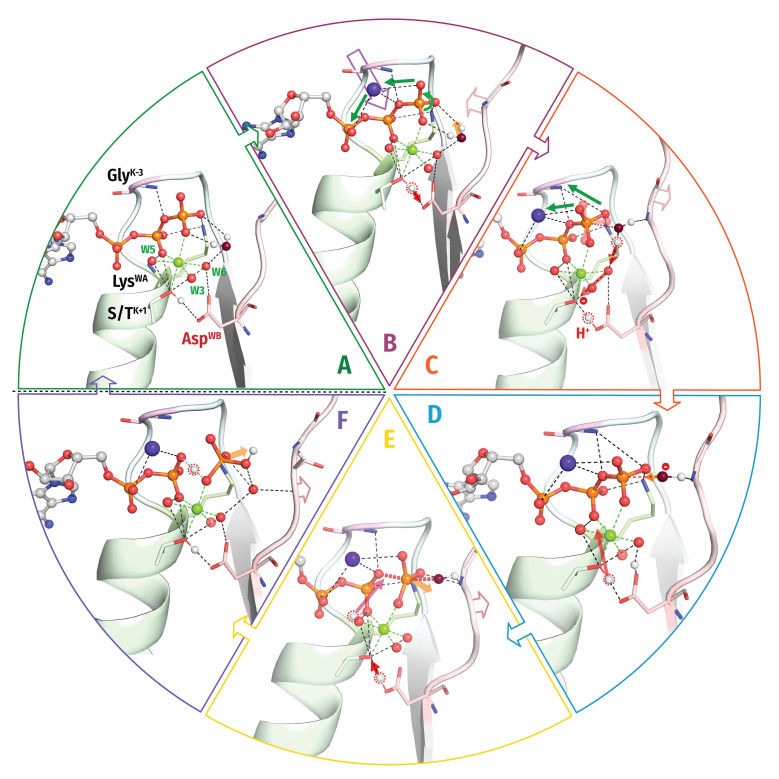
**Common scheme of stimulated hydrolysis in P-loop NTPases.** Empty purple arrow, insertion of the stimulator (shown as a purple sphere); pink arrows, movements of the WB-crest; green arrows, rotation and planarization of γ-phosphate; red arrows, proton displacements; orange arrows, detachment of Pi. The following crystal structures were used as templates: panels (**A**,**B**)—PDB ID 1FMW [140]; panels (**C**–**E**)—PDB ID 1VOM [93]; panel (**F**)—PDB ID 4PFP [41]. See the main text for a detailed explanation.

Building on the comparative structure analysis of over 3100 catalytic sites presented here and in the companion article [30], as well as on the available experimental and theoretical data, we describe the activated catalysis typical for P-loop NTPases by a simple mechanistic model depicted in Figure 12. We show the minimalistic version of the model that includes ubiquitous Walker A and Walker B motifs, Mg^2+^-NTP, a simple stimulator, such as a K^+^ ion or a Lys/Asn residue, and a few water molecules one of which serves as W_cat_. The model, however, can be easily expanded/adapted to fit distinct NTPase families by adding further stimulatory interactions, auxiliary and W_cat_-coordinating residues, as well as by replacing W_cat_ by other nucleophiles. 

According to the model, the catalytic transition proceeds in the following steps:

(A) A Mg-NTP complex binds to the Walker A and Walker B motifs; the binding energy is used to bring the NTP molecule into an elongated conformation with eclipsed β- and γ-phosphates and to surround the triphosphate chain by positively charged groups that are provided by the P-loop, WB-crest and, only in TRAFAC NTPases, Switch I loop. The accompanying protein conformational changes can power useful mechanical work. The catalytic site is further stabilized by the H-bond between Asp^WB^ and [Ser/Thr]^K+1^. The H-bond length is about 2.6–2.7 Å (Figure 8A); in this state Asp^WB^ is negatively charged.
(B) An exergonic interaction between the activating partner (another protein domain and/or an RNA/DNA molecule) and the WB-crest (i) shields and constricts the catalytic site, (ii) moves the WB-crest residues closer to the γ-phosphate, and (iii) inserts the stimulator(s) next to the phosphate chain. The constriction of the catalytic site shortens the H-bond between [Ser/Thr]^K+1^ and Asp^WB^ to 2.4–2.5 Å, turning Asp^WB^ into a potent proton trap. In most cases, (one of) the stimulator(s) links the O^2A^ and O^3G^ atoms of the triphosphate (Figure 1E,F, Figure 3B–D, Figure 4A–D, Figure 5A–C and Figure 6A) and twists γ-phosphate counter clockwise; the rotated γ-phosphate is stabilized by a new H-bond between O^2G^ and **HN**^K-3^.
  In other cases, the stimulators drag only γ-phosphate and, supposedly, twists it in some direction; see Figure 3A, Figure 5D and Figure 6B–D. The interaction of stimulators with γ-phosphate (i) increases the electrophilicity of the P^G^ atom, (ii) weakens the O^3B^–P^G^ bond, (iii) promotes the transition of γ-phosphate to a more planar conformation, and (iv) inevitably affects the coordination of the Mg^2+^ ion by displacing the O^1G^ atom. The increase of local positive charge at [Ser/Thr]^K+1^—after O^1G^ is moved aside by the stimulator—promotes the relocation of proton from [Ser/Thr]^K+1^ to Asp^WB^.
(C) The anionic [Ser/Thr]^K+1^ alkoxide withdraws a proton from the polarized W_cat_ molecule via intermediate proton carriers. Here, we depicted the simplest proton route as envisioned for TRAFAC NTPases (see Figure 7D and Figure 11D–F). More complex proton routes via W_cat_-coordinating Glu/Asp residues, as found in other classes of NTPases, are indicated by red dashed lines in Figure 4, Figure 5 and Figure 6 and Figure 7A and differently shaded red arrows in Figure 10 and Figure 11A–C.
(D) The resulting OH^—^_cat_, stabilized/polarized by its ligands, attacks the P^G^ atom. Although the simplified diagram in Figure 12D shows only one stabilizing interaction of OH^—^_ca_**_t_** with the **HN** group of the WB-crest residue, several ligands are usually involved in the stabilization; see Figure 1E,F, Figure 3, Figure 4, Figure 5 and Figure 6. During this step, the proton stays on Asp^WB^. The formation of a covalent bond between OH^—^_cat_ and P^G^ increases the planarization of γ-phosphate; its oxygen atoms repel the β-phosphate oxygen atoms, resulting in a lengthening of the O^3B^–P^G^ bond. With the inversion of γ-phosphate, increase in the O^3B^–P^G^ distance, and γ-phosphate moving away from β-phosphate, **HN**^K−3^ detaches from γ-phosphate and, together with Lys^WA^, Mg^2+^ and the stimulator, neutralizes the negative charge appearing on the O^3B^ atom, thereby lowering the activation barrier. In addition, the negative charge on β-phosphate attracts a proton from [Ser/Thr]^K+1^.
(E) The proton that comes from [Ser/Thr]^K+1^ forms a new short H-bond between β- and γ-phosphate [41,183,268], which further stabilizes the negative charge on the β-phosphate. The proton at Asp^WB^ relocates to [Ser/Thr]^K+1^.
(F) The H-bond between β- and γ-phosphate gradually dissociates as H_2_PO_4_^2^^−^ leaves the catalytic site. The departure of H_2_PO_4_^2^^−^ is an exergonic reaction that may be coupled to conformational changes, detachment of the activating partner from the WB-crest, and useful mechanical work.

The simplified diagram in Figure 12 does not show the activation partner, as they are different in different families of P-loop NTPases. Accordingly, the exothermic interactions of the activator with the amino acid residues of the NTPase domain (see Supplementary File S2), which apparently result in the constriction of the catalytic site, are also not depicted. Some of them, however, are shown in Figure S8, where the proposed general scheme is applied to myosin. Despite several sets of excellent crystal structures available [41,89,140,150,280,281,282,283,284], the mechanism of ATP hydrolysis by myosin remains unclear [285]. Using the entire set of myosin crystal structures and the scheme in Figure 12, we attempted to reconstruct the catalytic cycle of myosin as shown in Figure S8 and described in its caption. 

## 5. Conclusions and Outlook

Here, and in the companion article [30], we applied the tools of evolutionary biophysics to elucidate the mechanisms of catalysis by P-loop NTPases. This approach allowed us not only to find support for previous MD simulation data on rotating γ-phosphate by the stimulatory moiety in P-loop GTPases [29,286] but also, rather unexpectedly, to suggest a common catalytic mechanism for P-loop NTPases. 

The key elements of the proposed mechanism are the activation-induced, oppositely directed changes in proton affinity of H-bonded [Ser/Thr]^K+1^ and Asp^WB^, which convert the latter into a deep trap for a proton from [Ser/Thr]^K+1^. The latter, in turn, withdraws a proton from W_cat_, thereby, yielding a strong nucleophile OH^—^_cat_. The suggested mechanism rationalizes the strict evolutionary conservation of [Ser/Thr]^K+1^ and Asp^WB^, neither of which has been ascribed a specific catalytic function thus far. 

Indeed, Walker A and Walker B motifs—together—contain only two strictly conserved residues capable of proton trapping—namely, [Ser/Thr]^K+1^ and Asp^WB^. It is amazing that this H-linked pair has not been considered in relation to the catalytic proton transfer from W_cat_ thus far.

As argued elsewhere, the ability to provide strong acids and bases in a timely manner is important for enzyme catalysis [222]. Specifically, enzymes were suggested to generate strong bases or acids—precisely when required—by transiently altering the length of relevant H-bonds [142,256,287,288,289,290,291,292,293]. In the case of P-loop NTPases, the mechanism is simple: the free energies of (i) substrate binding and (ii) interaction with the activation partner are used to close and constrict the catalytic site, which shortens the H-bond between [Ser/Thr]^K+1^ and Asp^WB^ and levels their functional pK values. Eventually, after γ-phosphate is rotated by the stimulator, the proton relocates from [Ser/Thr]^K+1^ to Asp^WB^ yielding a serine/threonine alkoxide as a strong nucleophile for W_cat_. 

The still prevalent notion that chemically different catalytic bases (phosphates in the TRAFAC class enzymes vs. glutamates in most other P-loop NTPases) perform the same key function in different classes of P-loop NTPases is bizarre, to say the least. Furthermore, neither phosphates nor glutamates are common as catalytic bases in other families of phosphate transferases. In one of the most comprehensive reviews on their mechanisms, Cleland and Hengge even indicated the oddness of the anticipated catalytic bases in P-loop ATPases: “… more specifically, there must be a path for one proton of the attacking water molecule to reach a suitable acceptor. ATPases appear not to use general bases, such as the aspartates usually found in kinase active sites….” (quoted from [52]). Our message is simple: P-loop NTPases do use aspartates as “endpoint” catalytic bases similarly to most other phosphate transferases. 

The proposed mechanism of proton transfer from W_cat_ to Asp^WB^ via Mg^2+^-coordinating ligands brings the P-loop NTPases into the general context of other Mg-dependent hydrolases and transferases [49,50,52,134,260,294,295]. The full range of theoretical approaches developed for such enzymes can now be applied to P-loop NTPases. 

In particular, the relationship between changes in the Mg^2+^ coordination shell, proton affinity of Mg^2+^ ligands, and catalysis is the subject of QM/MM modelling in this field; see, e.g., [296]. Nemukhin and colleagues recently suggested proton transfer via the Mg^2+^ coordination shell in adenylate cyclase [295], which corroborates with the here proposed mechanism of proton transfer in P-loop NTPases. Therefore, we suggest that a new generation of QM/MM models of P-loop NTPases, which will include the entire Mg^2+^ coordination shell as well as proton transfer networks around Asp^WB^, will help to quantitatively describe their catalytic mechanisms. 

Our tentative identification of the anionic [Ser/Thr]^K+1^ alkoxide as the proton acceptor from W_cat_ brings P-loop NTPases into another broad context of enzymes that generate a strong nucleophile by stripping a Ser/Thr residue of its proton [52,260,261,262]. These are numerous families of serine proteases where, within apparently similar but non-homologous “catalytic triads”, a proton is transferred from the catalytic Ser to a conserved aspartate residue via a histidine residue that apparently serves as a Grotthuss-type proton carrier. It is less known that the proton-accepting Asp residue of serine proteases is usually H-bonded to another conserved Thr/Ser residue [288,297,298]. 

In a similar way, the catalytic Ser/Thr of eukaryotic protein kinases gives its proton to the conserved Asp [178,299,300], which, in turn, is also H-bonded to another conserved Ser/Thr residue. 

In essence, we argue that P-loop NTPases use the same (bio)chemical strategies to produce a strong nucleophile as many other enzymes do; there is no reason to consider them different or special in this respect.

Notably, serine proteases, serine-threonine kinases, Mg-dependent hydrolases and transferases, as well as the P-loop NTPases considered here, form the largest known enzyme families. Many of them use H-bonded [Asp/Glu]–[Ser/Thr] functional modules in different roles, a feature they share with the membrane-embedded, energy converting machinery of PRC and BR; see Figure 9 and [238]. We consider that a further search for structural modules common to different enzyme families is a very promising task. 

Last but not least, the protonation of aspartate and glutamate residues can be traced in real time by IR spectroscopy [200,220,221,230,239,246,269,272,275]. Therefore, we believe that the proton shuttling between [Ser/Thr]^K+1^ and Asp^WB^ in various P-loop NTPases is worthy of being traced using photoactivated, caged ATP/GTP substrates and modern IR spectroscopy techniques; see, e.g., [28,176,269,301]. Additionally, the position of a hydrogen atom within a short H-bond can be identified by complementary X-ray and neutron crystallographic structure determination [291,292,293]. The investigation of P-loop NTPases by these methods will shed new light on enzyme mechanisms. 

## Figures and Tables

**Table 1:** Structural traits of representative P-loop NTPases of different classes.

P-loop Class, Activation Mechanism	Representative Protein Structure	Site ID in Table S1	Figure in the Text	PDB Entry ID, Resolution	NTP or NTP Analog	Walker A Motif (K-3 and K+1 Residues)		Walker B Asp/Glu	Stimulatory Moieties	Coordination of W_cat_ *^a^*
**Kinase-GTPase Division**										
**TRAFAC** Interaction with the activating partner or dimerization in the presence of the activating partner leads to the stabilization of the Switch I loop and insertion of diverse stimulatory moieties into the catalytic site	RhoA	3508	1C,E	1OW3, 1.8 Å	GDP:MgF_3_^−^	Ala15	Thr19	Asp59	Arg85†-NH2 (AG)	Gln63^D+4^-OE1, Thr37^SwI^-CO
	MnmE	236	2A	2GJ8, 1.7 Å	GDP:AlF_4_^−^	Asn226	Ser230	Asp270	K^+^ ion (AG)	Thr251^SwI^-CO, Gly249^T−2^-HN, Thr250^T−1^-HN, Gly273^D+3^-HN, Gly273^D+3^-CO*
	Dynamin	3550	3A	2X2E, 2.0 Å	GDP:AlF_4_^−^	Ser41	Ser45	Asp136	K^+^ or Na^+^ ion (G)	Thr65^SwI^-CO, Gly139^D+3^-HN, Gly139^D+3^-CO*, Gln40^K−4^-OE1*
	Atlastin	3661	3B	6B9F, 1.9 Å	GDP:AlF_4_^−^	Arg77	Ser81	Asp146	Arg77^K−3^-NH2 (AG)	Gly149^D+3^-HN, Thr120^SwI^-CO, Gly149^D+3^-CO*, Asp152^D+6^-OD2*
	Gα_3_	3545	3C	2ODE, 1.9 Å	GDP:AlF_4_^−^	Glu43	Ser47	Asp200	Arg178^T−3^-NH1 (AG)	Thr181^SwI^-CO, Gln204^D+4^-OE1, Gly203^D+3^-HN
	Myosin II	42	3D	1VOM, 1.9 Å	ADP-VO_4_^3−^	Gly182	Thr186	Asp454	Asn233^S−4^-ND2 (AG)	Ser237^SwI^-CO, Ser236^S−1^-OG, Gly457^D+3^-HN, Gly457^D+3^-CO, Arg238^S+1^*-NH1,Glu459^D+5^*-OE1
**SIMIBI** Monomeric domains dimerize and provide activating Lys or Arg fingers for each other in response to the interaction with the activating partner	GET3	64	4A	2YNM, 2.1 Å	ADP:AlF_4_^−^	Gly39	Ser43	Asp151	Lys37†-NZ (AG)	Asp66^WB+1^-OD2, Asp155†-OD1, Lys68^WB+1^-NZ, Gly154^D+3^-HN, Lys37†-HN*
	Signal recognition particle	3522	4B	2CNW, 2.39 Å	GDP:AlF_4_^−^	Gly108	Thr112	Asp187	Arg138-NH1 (AG)	Gly190^D+3^-HN, Asp135^WB+1^-OD2, GTP†-O3′*, Gly190^D+3^-CO*, Glu284†-OE1*
**Kinases** Rearrangement of the lid domain upon binding of the second substrate leads to the insertion of Arg/Lys fingers into the catalytic site	Thymidylate kinase	1207	4C	1NN5, 1.5 Å	ANP	Arg16	Ser20	Asp96	Arg16^K−3^-NH1 (AG?), Arg97^D+1^-NH2 (G)	The second substrate is coordinated by Arg45^WB+1^-NH2, Arg-97^D+1^-NE, Glu149^Lid^-OE1, Pro43-CO*
	Adenosine 5′-phosphosulfate kinase	1490	4D	4BZX, 1.7 Å	ANP	Gly453	Ser457	Asp478	Lys562^Lid^-NZ (G)	The second substrate is coordinated by Asp41^D+2^-OD1, Lys562^Lid^-NZ, Arg483^D+5^-NH2, Arg497-NH1
	Adenylate kinase	xxx0	S8B	3SR0, 1.6 Å	ADP:AlF_4_^−^	Gly10	Gly14	Asp81	Arg124^Lid^-NH1 (AG) Arg124^Lid^-NH2 (G) Arg150^Lid^-NH1 (G) Arg161^Lid^-NH1(G)	The second substrate (phosphate acceptor) is coordinated by Arg150^Lid^-NH2, Arg85^D+4^-NH1, Arg85^D+4^-NH2, Arg36^WB+1^-NH1, Arg36^WB+1^-NH2
**P-loop class,** activation mechanism	Representative protein structure	Site ID in Table S1	Figure in the text	PDB entry ID	NTP or NTP analogue	Walker A motif (K-3 and and K+1 residues)		Walker B motif, [Asp/Glu]^WB^	Stimulatory moieties	Coordination of W_cat_*^a^*
**ASCE Division**										
**AAA+/**SF3 ATPases In a hexamer, the binding/hydrolysis of ATP in one subunit causes conformational changes activating the adjacent subunit; the activation involves class-specific helical domain	N-ethylmaleimide sensitive factor	359	5A	1NSF, 1.9 Å	ATP	His546	Thr550	Asp603	Lys708‡-NZ (AG) Lys631†-NZ (G)	Asp604^D+1^-OD2, Lys631†-NZ, Ser647^WB−1^-OG
	SV40 large T antigen helicase (SF3)	372	5B	1SVM, 1.9 Å	ATP	Asp429	Thr433	Glu473	Lys418†-NZ (AG), Arg540†-NH2 (G)	Asp474^E+1^-OD1, Asn529^WB−1^-OD1, Arg498†-NH1, Arg540†-NH2
**Helicases SF1/2:** Rearrangement of the C-terminal domain upon DNA or RNA binding leads to the insertion of an Arg finger into the nucleotide-binding domain	Chikungunya virus nsP2 helicase (SF1)	N/A	1D, 1F	6JIM, 2.0 Å	ADP:AlF_4_^−^	Gly189	Ser193	Asp252	Arg312‡-NH2 (AG), Arg416‡-NH1 (G), Arg416‡-NH2 (G),	Glu253^D+1^-OE2,Gln283^D+1^-OE2, Gly384‡-HN
	HCV NS3 helicase (SF2)	136	5C	3KQL, 2.5 Å	ADP:AlF_4_−	Gly207	Ser211	Asp290	Arg467‡-NH2 (AG), Arg467‡ -NH1 (G), Arg467‡ -NH2 (G), Arg464‡ -NH1 (G), Arg464‡ -NH2 (G),	Glu291^D+1^-OE1,Gln460‡-OE1, Arg464‡-NH2,Gly417‡-NH, Ala323-NH^WB+1^*
	Multifunctional helicase Pif1p (SF1)	233	11B	5O6B, 2.0Å	ADP:AlF_4_^−^	Gly261	Ser265	Asp-341	Arg417‡-NH2 (AG)	Glu342^D+1^-OE1,Gln381^WB−1^-OE1, Arg734‡-NH1, Gly709‡-HN
**ABC ATPases:** Monomeric domains dimerize and provide activating LSGGQ motifs for each other in response to the substrate binding	Maltose transporter	154	5D	3PUW, 2.2 Å	ADP:AlF_4_^−^	Gly39	Ser43	Asp158	Ser135†-OG (G), Gly137†-HN (G)	Gln82^WB+1^-NE2,Glu159^D+1^-OE1, Glu159^D+1^-OE2,Asn163†-CO, His192 ^WB−1^-NE2
**F_1_/RecA-like:** In an oligomer, ATP binding/hydrolysis in one subunit causes conformational changes that activate the adjacent subunit by inserting Arg/Lys fingers	F_1_-ATPase	18	6A	1H8E, 2.0 Å	ADP:AlF_4_^−^	Gly159	Thr163	Asp256	Arg373†-NH1 (AG), Arg189^WB−1^-NH1 (G), Arg189^WB−1^-NH2 (G),	Glu188^WB−1^-OE1, Arg260^D+4^-NH2, Ser344†-CO
	Replicative helicase DnaB	N/A	6B	6T66, 3.9 Å	GDP-AlF_4_^−^	Ser231	Thr235	Asp340	Arg439†-NE (G), Lys437†-NZ (G)	Glu259^WB+1^**, Tyr341^D+1^** Gln381^WB−1^**Arg439†
	Circadian clock protein KaiC	683	6C	4TL7, 1.9 Å	ATP	Gly49	Thr53	Asp145	Lys224†-NZ (G) Arg226†-NH2 (G)	Glu183^WB−1^-OE1**, Ser146^D+1^**, Phe199†-CO
	Recombinase RadA	1376	6D	3EW9, 2.4 Å	ANP	Gly108	Thr112	Asp211	K^+^-503 (G), K^+^-504 (G)	Glu151^WB+1^**, Ser212^D+1^** Gln257^WB−1^**,
	RecA	100	N/A	3CMX, 3.4 Å	ADP:AlF_4_^−^	Ser69	Thr73	Asp144	Lys248†-NZ (G) Lys250†-NZ (G)	Glu96^D+1^**, Phe216**†-CO, Gln194**^D−1^,

Residue numbers are as in the listed PDB structures; *^a^*—polar atoms, located within 3.6 Å from the catalytic water molecule; AG—the stimulatory residue inserts between α-phosphate and γ-phosphate (or its mimic). G—the stimulatory residue is only coordinating γ-phosphate or its mimic. †—residue from a polypeptide chain other than the P-loop containing one; ‡—residues from a domain other than the described P-loop domain; *—residue coordinates the attacking water molecule via another water molecule (e.g., Figure 4B); **—catalytic water molecule not resolved, the coordinating residue(s) were inferred from structure superposition and literature data.

## Data Availability

Descriptions of each analysed catalytic site are available in Table S1. Scripts used to generate and annotate the data and quickly visualize selected sites listed in Table S1 are available from github.com/servalli/pyploop (accessed on 22 June 2022).

## Supplementary Materials

The following files with supporting information can be downloaded at: https://www.mdpi.com/article/10.3390/biom12101346/s1. ***Supplementary*** Figures S1–S8 ***with captions***; ***Supplementary File S1*** (Consideration of tentative activation mechanisms in STAND, KAP, VirD/PilT and FtsK-HerA classes of P-loop ATPases); ***Supplementary File S2*** (Transition state analogues and energetics of P-loop NTPases); ***Supplementary File S3*** (Local molecular electric field in the catalytic sites of P-loop NTPases); Supplementary Statistics File (an Excel file with a first Description Sheet); ***Supplementary Table S1*** (Results of the computational analysis of all available structures of the P-loop proteins in complex with NTPs and NTP-like molecules); the Sheet A of the Microsoft Excel table contains the list with characteristics of all analysed structures, together with indicated key functional residues of the Walker A and Walker B motifs, Arg, Lys, and Asn fingers, as well as distances from (1) the respective atoms of NTPs/their analogues to the K-3 residues and Arg/Lys fingers, as well as (2) from [Asp/Glu]^WB^ to [Ser/Thr]^K+1^. All columns present in the Sheet A of the Table S1 (data) are described in the Sheet B. Each row contains the data for one catalytic site in one structure. Catalytic sites containing “properly bound” NDP:AlF_4_^-^ complexes that we deemed to be reliable TS-analogues (see Table S3 in the companion paper [30] for detailed description of AlF_4_^−^ and Mg^2+^ binding in such structures) are marked with “y” or “*” in column “site rel”; they are coloured green. The sites with “improperly bound” NDP:AlF_4_^-^ complexes are coloured pink. ***Supplementary Table S2*** (Analysis of the bonding pattern within the catalytic sites of the RhoA GTPase and F_1_-ATPase as a function of the TS analogue bound), ***Supplementary Table S3*** (The pKa values of Asp256^WB^/“catalytic” Glu188 of the bovine ATP synthase as calculated for different states of the catalytic pocket by application of the PROPKA v. 2.0 software).
