# Supplementary material for: Common Mechanism of Activated Catalysis in P-loop Fold Nucleoside Triphosphatases—United in Diversity
Source: Biomolecules. 2022 Sep 22;12(10):1346. doi: 10.3390/biom12101346 (PMC9599734; doi:10.3390/biom12101346)
Supplement: Supplementary file 1 [file biomolecules-12-01346-s001.zip › Biomolecules_1832871_Supplementary_Figures.pdf]

Supplementary Figures to the manuscript "Common mechanism of activated catalysis in P-loop fold nucleoside triphosphatases - "united in diversity" by Maria I. Kozlova, Daria N. Shalaeva, Daria V. Dibrova, Armen Y Mulkidjanian

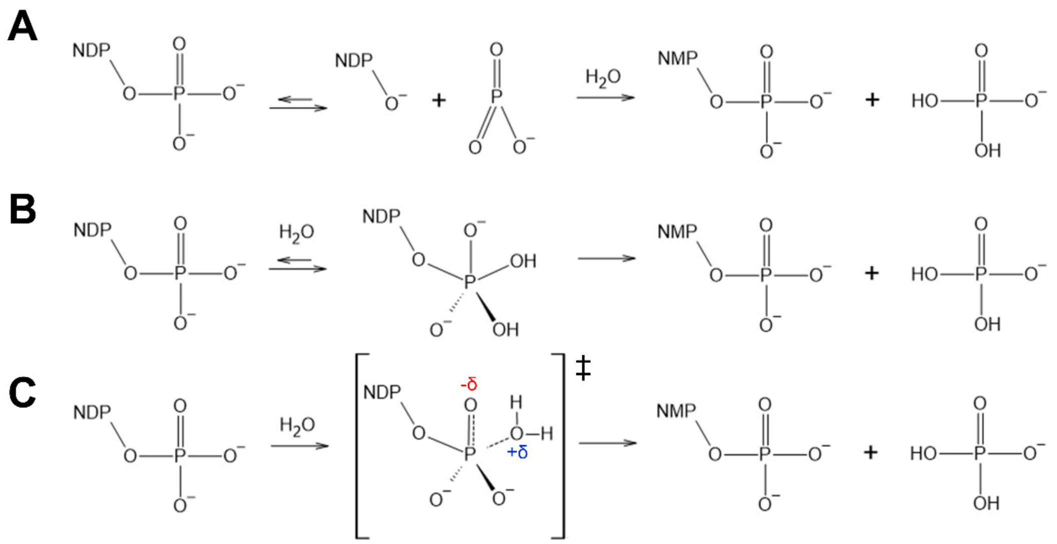

Figure S1. Tentative mechanisms of NTP hydrolysis. Dissociative (**A**), associative (**B**), and concerted pathways (**C**). See the main text for further details and references.

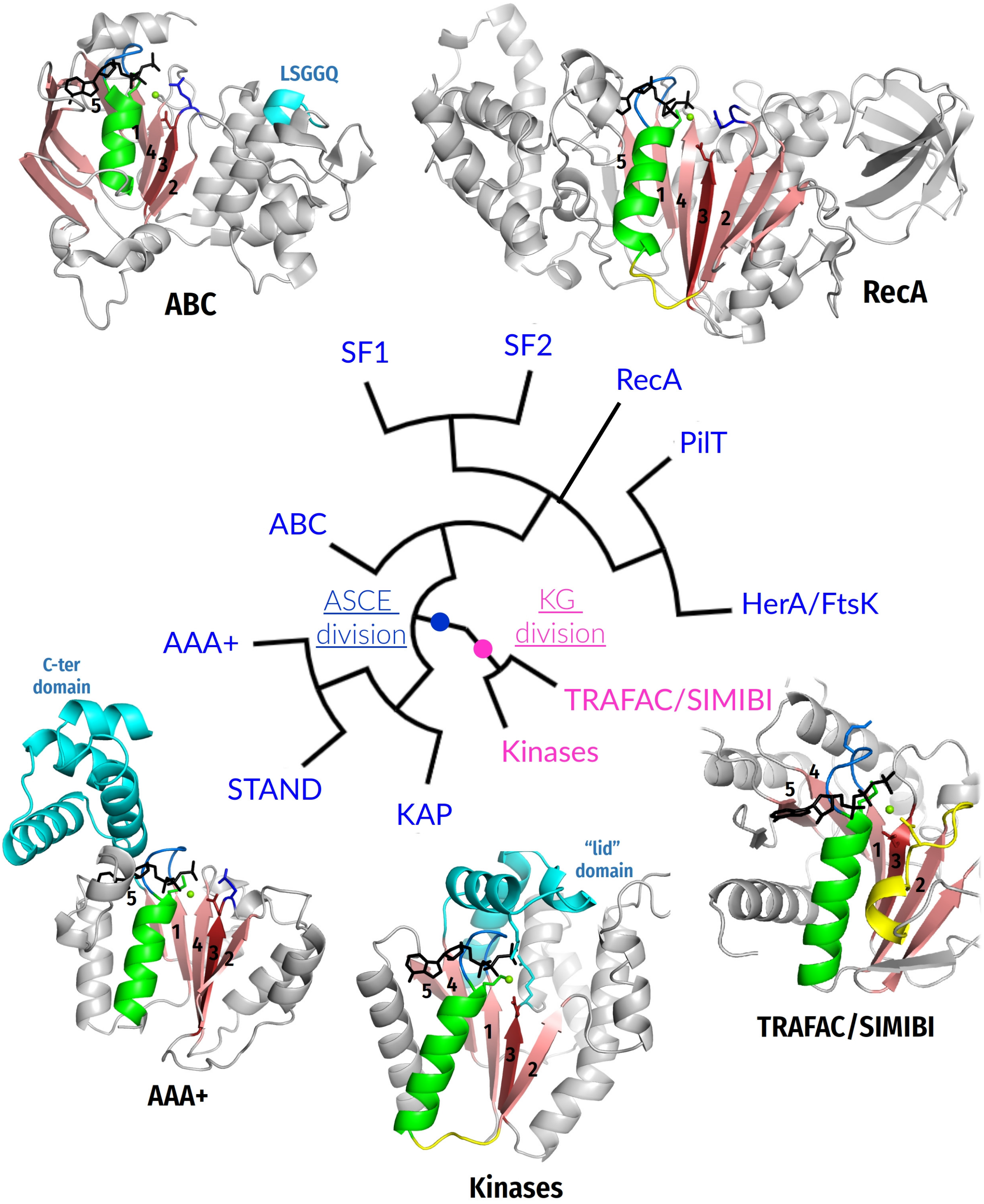

Figure S2. Cladogram of higher-order relationships between major divisions/classes of P-loop NTPases with depicted typical structures.

The cladogram (as modified from [1]) shows the two major divisions: the kinase-GTPase (KG) division and the ASCE (additional strand, catalytic E) division. The β-strands forming the cores

of P-loop domains are numbered in a traditional way [1, 2]. These β-strands are colored pink, the P-loop is shown in blue, the following α_1-helix is shown in green, the K-loop/Switch I in TRAFAC class NTPases as well as the corresponding loops in other NTPases are colored in yellow, NTP analogs are shown as black sticks, Mg^{2+} ions are shown as green spheres, the rest of proteins is shown as gray cartoons. The reference residues of Walker A and Walker B motifs (lysine (K) and aspartate (D), respectively), as well as the catalytic glutamate (E) in ASCE NTPases are shown as sticks. The following reference crystal structures are depicted: ABC – antibacterial peptide ABC transporter McjD of *Escherichia coli*, PDB ID 5EG1 [3]; RecA/F_1 –F_1-ATPase of *Caldalaklibacillus thermarum*, PDB ID 5HKK [4]; AAA+ –clamp loader γ-subunit *E. coli*, PDB ID 1NJG [5]; Kinases –thymidylate kinase of *E. coli*, PDB ID 4TMK [6]; TRAFAC/SIMIBI – nitrogenase ATPase subunit of *Azotobacter vinelandii*, PDB ID 4WZB [7].

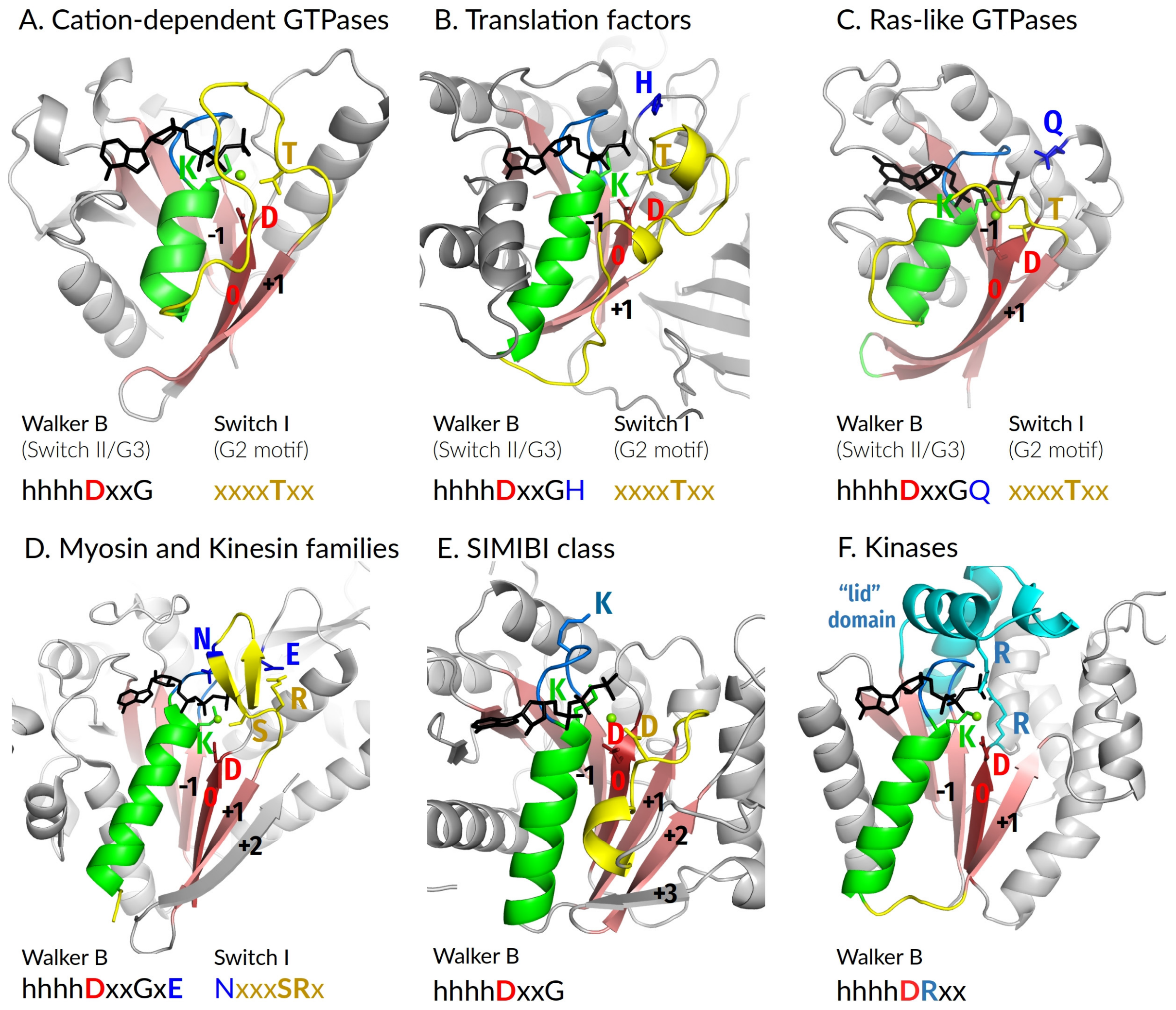

Figure S3. P-loop domain organization and conserved motifs in P-loop NTPases of the Kinase-GTPase division.

The β-strands forming the cores of P-loop domains are numbered relative to the Walker B motif-containing WB-strand as suggested in the main text. Color code as in Fig. S2. The following crystal structures are depicted: A, MnmE GTPase of *E. coli*, PDB ID 2GJ8 [8]; B, Elongation factor EF-Tu of *Thermus thermophilus* HB8, PDB ID 4V5L [9]; C, Human Ras-GTPase, PDB ID 1WQ1, [10]; D, Myosin II of *Dictyostelium discoideum*, PDB ID 1W9L; E, nitrogenase ATPase subunit of *Azotobacter vinelandii*, PDB ID 4WZB [7]; F, thymidylate kinase of *E. coli*, PDB ID 4TMK [6].

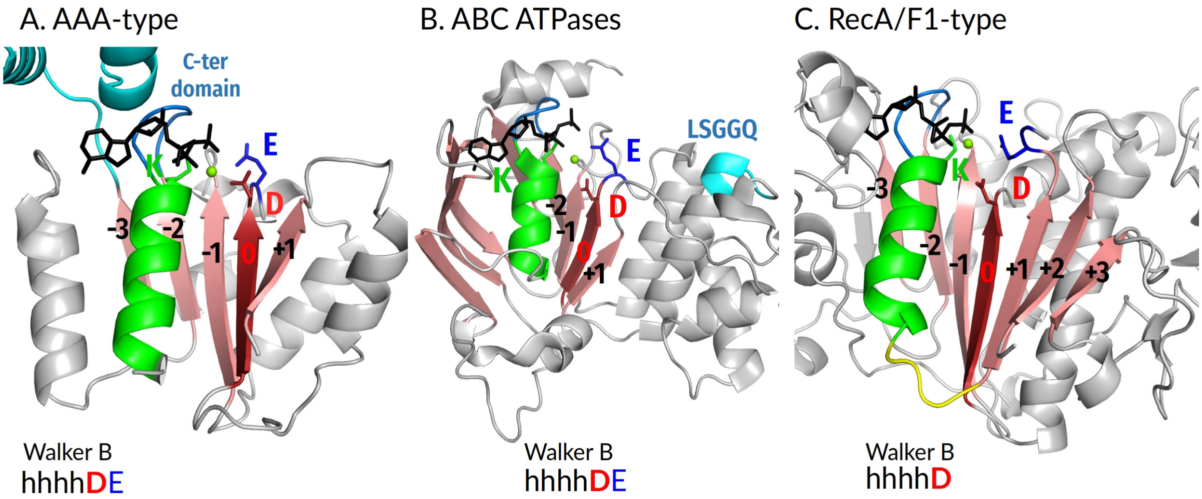

Figure S4. P-loop domain organization and conserved motifs in P-loop NTPases of the ASCE division.

The β-strands forming the cores of P-loop domains are numbered relative to the Walker B motif-containing WB-strand as suggested in the main text. Color code as in Fig. S2. The following crystal structures are depicted: A, AAA+ Clamp Loader Gamma Subunit of *E. coli*, PDB ID 1NJG [5]; B, antibacterial peptide ABC transporter McjD of *E.coli*, PDB ID 5EG1 [3]; C, F_1-ATPase of *Caldalaklibacillus thermarum*, PDB ID 5HKK [4].

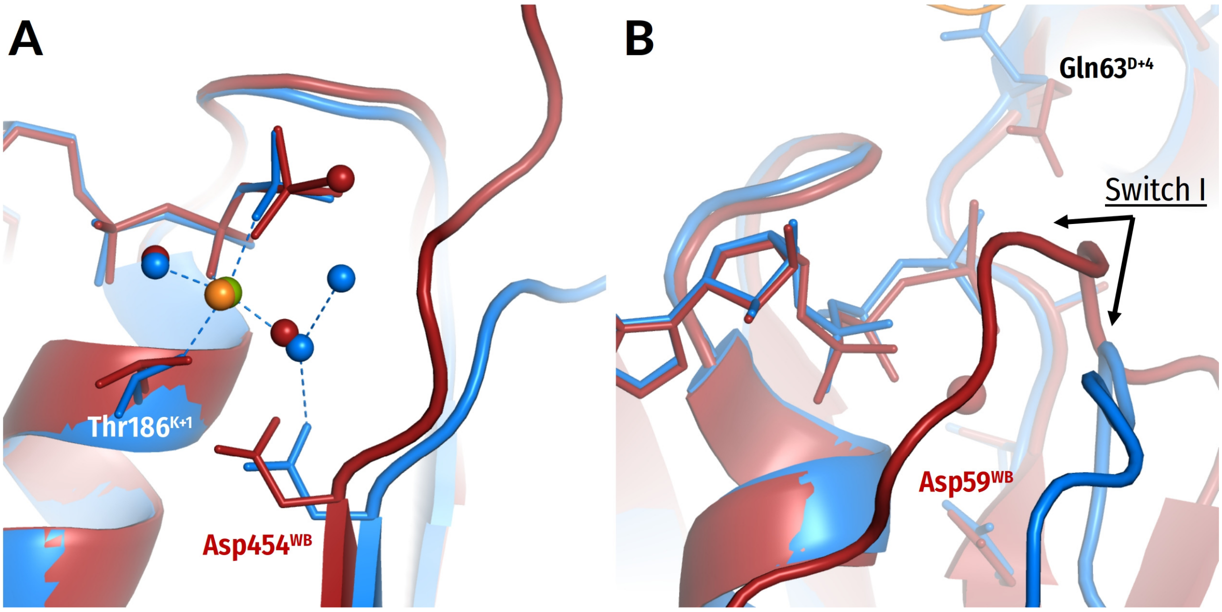

Figure S5. Structures of same P-loop NTPases with transition state analogs and substrate/substrate analogs bound, respectively.

The structures with bound substrate or substrate analogs are shown in blue with their Mg^{2+} ions in green; the structures with transition state analogs bound are shown in dark red with their Mg^{2+} ions in orange. Cations and water molecules are shown as spheres. The following crystal structures are depicted**:** **A,** Myosin II from *Dictyostelium discoideum* in complex with a bound ATP molecule (blue, PDB ID 1FMW [11]) and with a transition state analog ADP:VO_4^- bound (dark red, PDB ID 1VOM [12]); **B,** Ras-like GTPase RhoA with a bound non-hydrolyzable substrate analog GNP (blue, PDB 6V6M, [13]) and a complex of Rho with RhoGAP and a transition state analog GDP:MgF_3^- (dark red, ODB 1OW3, [14]).

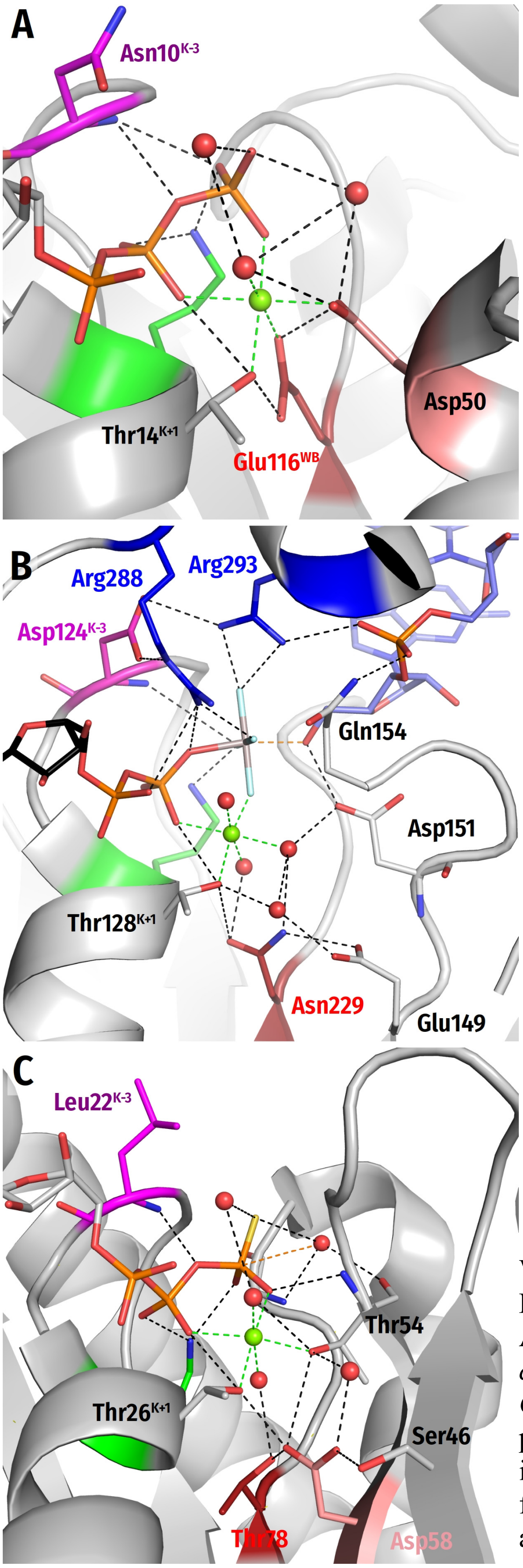

Figure S6. Deviations from a typical Walker B motif. Color code as in Fig. 1E,F.

A. In a few cases, which are listed in [15], a glutamate residue occupies the position of Asp^{WB}. In dethiobiotin synthetase, the long side chain of Glu^{WB} reaches Mg^{2+} and directly coordinates it, forming also a H-bond with [Ser/Thr]$^{K+1}$, as shown in Panel A for the for the enzyme from *Helicobacter pylori* complexed with GTP (PDB 3QXJ [16]). In In dethiobiotin synthetase, the γ-phosphate group is transferred on the carboxy group of 7,8-diaminononanoate, which is likely to be deprotonated at neutral pH, so that no proton trapping is needed. In a few more conventional cases, the Glu^{WB} residue, similarly to Asp^{WB}, does not reach Mg^{2+}, interacts with Mg^{2+}-coordinating water and makes an H-bond with [Ser/Thr]$^{K+1}$, see [15].

B. An Asn229 residue links W3, W6 and Thr131^{K+1} in the wild-type polynucleotide kinase of *Caenorhabditis elegans,* see Panel B for its structure with bound ssRNA dinucleotide GC and ADP:AlF$_4^-$ (PDB ID 4OI1 and [15, 17]). In this case, however, the adjacent WB+1 strand has a ^{149}ELD^{151} triad at its C-cap. In the TS-like AlF$_4^-$-containing structure, the Glu149 residue is connected via a bridging water molecule to Thr131^{K+1} and to W3, whereas Asp151 makes a short H-bond (2.48 Å) with the hydroxyl of the RNA ribose. Deprotonation of this hydroxyl yields the nucleophile that attacks the γ-phosphate. We believe that the functions of Asp^{WB} are divided between Asn229 and Glu149 in this polynucleotide kinase. The Asn229^{WB} residue serves as a structural linker to the Walker A motif, whereas Glu149 serves as a trap for the proton that is taken by Asp151 from W$_{cat}$.

While the same Asn-Glu-Asp triad is found in other Metazoa enzymes, Asp substitutes for Asn229 and Asn substitutes for Glu149 in *Saccharomyces cerevisiae*, *Schizosaccharomyces pombe*, and *Candida albicans.* Hence, in evolutionarily primary primitive microorganisms, a single Asp^{WB} appears to interact with W6 and Thr^{K+1} and to obtain a proton from the catalytic Asp residue at the C-cap of the adjacent WB+1 strand; in the *C. elegans* structure

(PDB ID 4OI1 [17]), Asn229 and Asp 151 are linked by W6. The "primordial" dyad of aspartates is functionally similar but structurally distinct from the $Asp^{WB}L^{D+1}Asp^{D+2}$ aspartate dyads found in some other kinase families, see Fig. 4D and [18]. Generally, the overall diversity of proton routes within kinases [18] might reflect the diversity of second substrates in these enzymes, see Section 2.2.

C. A natural mutation in an otherwise typical small TRAFAC class GTPase MglA changed Asp^{WB} to Thr. The structure of MglA GTPase of *Myxococcus xanthus* with a bound non-hydrolizable analog of GTP is shown (PDB ID: 6IZW and [19]). The enzyme retained its function owing to the appearance of an Asp residue at the N-terminus of the adjacent antiparallel WB+1 strand [15]. The resulting topology of the catalytic site is typical for P-loop NTPases.

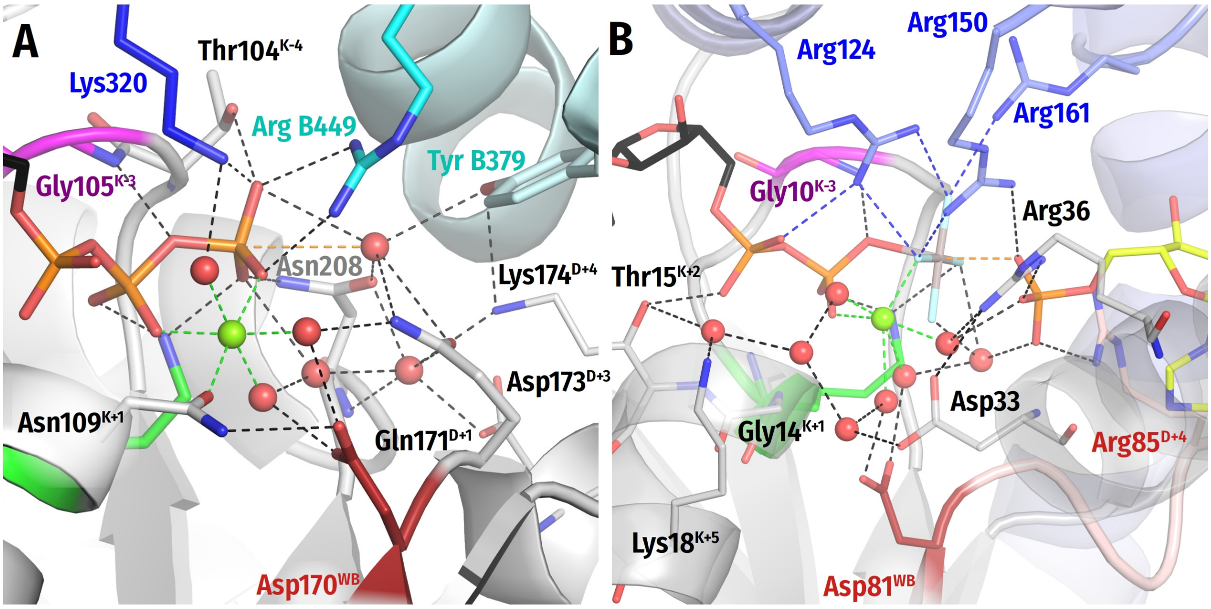

Fig. S7. Residues other than [Ser/Thr] in the K+1 position of the Walker A motif. Colors as in Fig. 3-6 in the main text. Lys^{WA} shown in green, Asp^{WB} in dark red, adjacent monomer and its residues in cyan, Arg and Lys finger from the same polypeptide chain in blue.

A, an asparagine residue replaces [Ser/Thr]$^{K+1}$ in such distantly related AAA+ ATPases as torsinA of metazoa and DnaC helicase loader of some enterobacteria including *E. coli* [15, 20, 21]. In addition, the potentially W_{cat}-stabilizing "catalytic" [Glu/Asp]$^{D+1}$ – ubiquitous in AAA+ ATPases [22] – is replaced by Asn^{D+1} in torsinA (but not in DnaC). The ATPase activity of both these proteins are very low, so it is not yet clear if they are true ATPases or simply work as switches that change their conformation depending on whether ATP or ADP is bound in the catalytic site [20, 21, 23, 24]. Shown is the high-resolution ATP-containing structure of human torsinA in complex with its activator LULL1 (PDB ID 5J1S [25]). It can be seen that Asn109^{K+1} makes the canonic H-bond with Asp170^{WB} which could increase the proton affinity of the latter. While Asn171^{D+1} cannot transfer a proton from W_{cat}, a water chain – similar to one observed in TRAFAC class NTPases (Fig. 7B-D, 11D-F) – connects W_{23} with Asp170^{WB} via the Mg^{2+}-coordinating W6 molecule. In AAA+ NTPases, a Lys/Arg residue always interacts with γ-phosphate and, supposedly, W_{cat} (see section 2.2.2.2, Fig. 5A,B, and [26, 27]). In torsinA, this is the Arg449 residue of LULL1. In the absence of negatively charged Asp/Glu^{D+1}, the interaction of a positively charged Arg449 with W_{cat}, by dramatically decreasing its proton affinity, may trigger proton transfer from W_{cat}, via W6, to Asp170^{WD}. Subsequently, the proton can move to β-phosphate via W6. Hence, the structure is compatible with involvement of Asp170^{WB} as a proton trap in torsinA and could explain its, albeit slow, ATPase activity.

B, Adenylate kinase of *Aquifex aeolicus* ([PDB 3SR0] [28]), see the main text.

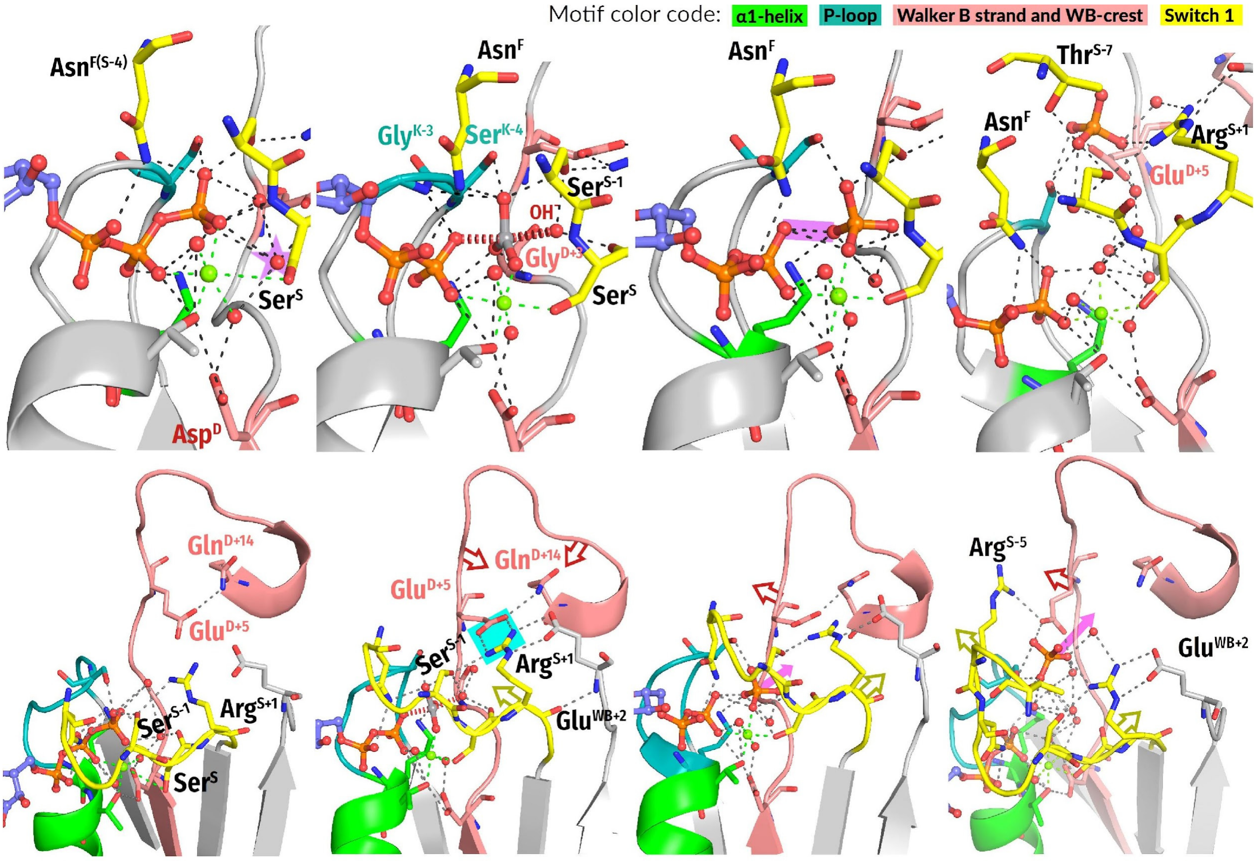

A. Pre-catalytic state 1fmv

B. Transition state 1vom

C. P_i release, early 4pfp

D. P_i release, late 4pjn

Figure S8. Catalytic cycle of myosin as inferred from the available set of crystal structures. The top and bottom rows show similar structures differently zoomed.

Given that myosin is one of the few P-loop NTPases with a set of high-quality crystal structures including those mimicking the pre-transition, transition, and several post-transition states (see also Supplementary Table 1), we tentatively reconstruct the catalytic cycle of myosin as follows:

A) (top) In the substrate-bound state (ATP-soaked myosin crystals, PDB ID 1FMW [11]), the catalytic pocket is not constricted, and the proton pathway from the would-to-be W_{cat} in the W_{12} position (highlighted with a star) to Asp^{WB} (via W6) is clearly seen.

(bottom) In this state, Glu^{D+5} of WB-crest and Arg^{S+1} of Switch I motif are remote from each other, and each residue is only interacting with its neighbors in the polypeptide chain. Glu^{D+5} is forming an H-bond with Gln^{D+14}, while Arg^{S+1} is in contact with Ser^{S-1} (bottom).

B) (top) In the ADP:VO_4^--containing structure, which mimics the transition state (PDB 1VOM [12]), the site is constricted, and W_{12}, after giving its proton via W6 to Asp^{WB}, is pushed upwards into the attack position of $\mathbf{OH^-_{cat}}$, here mimicked by the oxygen atom of VO_4^-. In this position, $\mathbf{OH^-_{cat}}$ is stabilized by the side chain of Ser^{S-1}, **CO** of Ser^S, and **HN** of Gly^{D+3}. Also in this state, the Asn finger comes closer to the phosphate chain and interacts with α-phosphate and one of the

oxygen atoms of VO_4^-, another oxygen atom of VO_4^- interacts with Gly^{K-3} and with the side chain of Ser^{K-4}. The WB-crest is brought closer to the catalytic site: an oxygen atom of VO_4^- is coordinated by Gly^{D+3}. Here, the distance between $\mathbf{OH^-_{cat}}$ and W6 is 4.8 Å, which, supposedly, precludes the proton at Asp^{WB} from returning to W_{cat}. The proton, however, can pass to β-phosphate; all the tentative proton transfer steps on the way to β-phosphate are of 2.9 Å.

(bottom) An extensive salt bridge network is formed between WB-crest, Switch I and the adjoining domain. Arg^{S+1} interacts with Ser^{S-1} and Glu^{D+5} (bonds highlighted in bright cyan), whereas subsequent residues of the WB-crest are contacting the adjoining protein domain.

C and D) In the two subsequent post-transition states (mimicked by differently P_i-soaked ADP-containing myosin structures with PDB IDs 4PFP and 4PJN [29]), catalytic site opens up: the release of $H_2PO_4^{2-}$ is coupled with breaking of salt bridges between WB-crest and Switch I. Hence the exergonic release of P_i drives the opening of the catalytic pocket, which, in turn, is coupled to the mechanical movement. The Asn finger no longer interacts with the outgoing $H_2PO_4^{2-}$ moiety. In the early post-transition state the proton is already engaged in the H-bond between O^{3B} and O^{3G} (C, top, the H-bond is highlighted in purple), while in the late post-transition state (D), hydrogen-bonded networks are further rearranged: the outgoing $H_2PO_4^{2-}$ no longer contacts residues involved in P_γ coordination during the previous steps (D, bottom); $H_2PO_4^{2-}$ gets directly engages with Glu^{D+5} and Arg^{S+1} pulling apart the WB-crest and Switch I (D, bottom).

References

[1] A. Krishnan, A.M. Burroughs, L.M. Iyer, L. Aravind, Comprehensive classification of ABC ATPases and their functional radiation in nucleoprotein dynamics and biological conflict systems, Nucleic Acids Res 48(18) (2020) 10045-10075.
[2] D.D. Leipe, Y.I. Wolf, E.V. Koonin, L. Aravind, Classification and evolution of P-loop GTPases and related ATPases, J Mol Biol 317(1) (2002) 41-72.
[3] S. Mehmood, V. Corradi, H.G. Choudhury, R. Hussain, P. Becker, D. Axford, S. Zirah, S. Rebuffat, D.P. Tieleman, C.V. Robinson, K. Beis, Structural and Functional Basis for Lipid Synergy on the Activity of the Antibacterial Peptide ABC Transporter McjD, J Biol Chem 291(41) (2016) 21656-21668.
[4] S.A. Ferguson, G.M. Cook, M.G. Montgomery, A.G. Leslie, J.E. Walker, Regulation of the thermoalkaliphilic F1-ATPase from Caldalkalibacillus thermarum, Proc Natl Acad Sci U S A 113(39) (2016) 10860-5.
[5] M. Podobnik, T.F. Weitze, M. O'Donnell, J. Kuriyan, Nucleotide-induced conformational changes in an isolated Escherichia coli DNA polymerase III clamp loader subunit, Structure 11(3) (2003) 253-63.
[6] A. Lavie, N. Ostermann, R. Brundiers, R.S. Goody, J. Reinstein, M. Konrad, I. Schlichting, Structural basis for efficient phosphorylation of 3'-azidothymidine monophosphate by Escherichia coli thymidylate kinase, Proc Natl Acad Sci U S A 95(24) (1998) 14045-50.
[7] F.A. Tezcan, J.T. Kaiser, D. Mustafi, M.Y. Walton, J.B. Howard, D.C. Rees, Nitrogenase complexes: multiple docking sites for a nucleotide switch protein, Science 309(5739) (2005) 1377-80.

[8] A. Scrima, A. Wittinghofer, Dimerisation-dependent GTPase reaction of MnmE: how potassium acts as GTPase-activating element, EMBO J 25(12) (2006) 2940-51.
[9] R.M. Voorhees, T.M. Schmeing, A.C. Kelley, V. Ramakrishnan, The mechanism for activation of GTP hydrolysis on the ribosome, Science 330(6005) (2010) 835-838.
[10] K. Scheffzek, M.R. Ahmadian, W. Kabsch, L. Wiesmuller, A. Lautwein, F. Schmitz, A. Wittinghofer, The Ras-RasGAP complex: structural basis for GTPase activation and its loss in oncogenic Ras mutants, Science 277(5324) (1997) 333-8.
[11] C.B. Bauer, H.M. Holden, J.B. Thoden, R. Smith, I. Rayment, X-ray structures of the apo and MgATP-bound states of Dictyostelium discoideum myosin motor domain, J Biol Chem 275(49) (2000) 38494-9.
[12] C.A. Smith, I. Rayment, X-ray structure of the magnesium(II).ADP.vanadate complex of the Dictyostelium discoideum myosin motor domain to 1.9 A resolution, Biochemistry 35(17) (1996) 5404-17.
[13] Y. Lin, S. Lu, J. Zhang, Y. Zheng, Structure of an inactive conformation of GTP-bound RhoA GTPase, Structure 29(6) (2021) 553-563 e5.
[14] D.L. Graham, P.N. Lowe, G.W. Grime, M. Marsh, K. Rittinger, S.J. Smerdon, S.J. Gamblin, J.F. Eccleston, MgF(3)(-) as a transition state analog of phosphoryl transfer, Chem Biol 9(3) (2002) 375-81.
[15] M. Kanade, S. Chakraborty, S.S. Shelke, P. Gayathri, A Distinct Motif in a Prokaryotic Small Ras-Like GTPase Highlights Unifying Features of Walker B Motifs in P-Loop NTPases, J Mol Biol 432(20) (2020) 5544-5564.
[16] P.J. Porebski, M. Klimecka, M. Chruszcz, R.A. Nicholls, K. Murzyn, M.E. Cuff, X. Xu, M. Cymborowski, G.N. Murshudov, A. Savchenko, A. Edwards, W. Minor, Structural characterization of Helicobacter pylori dethiobiotin synthetase reveals differences between family members, Febs J 279(6) (2012) 1093-105.
[17] A. Dikfidan, B. Loll, C. Zeymer, I. Magler, T. Clausen, A. Meinhart, RNA specificity and regulation of catalysis in the eukaryotic polynucleotide kinase Clp1, Mol Cell 54(6) (2014) 975-86.
[18] D.D. Leipe, E.V. Koonin, L. Aravind, Evolution and classification of P-loop kinases and related proteins, J Mol Biol 333(4) (2003) 781-815.
[19] J. Baranwal, S. Lhospice, M. Kanade, S. Chakraborty, P.R. Gade, S. Harne, J. Herrou, T. Mignot, P. Gayathri, Allosteric regulation of a prokaryotic small Ras-like GTPase contributes to cell polarity oscillations in bacterial motility, PLoS Biol 17(9) (2019) e3000459.
[20] M. Nagy, H.C. Wu, Z. Liu, S. Kedzierska-Mieszkowska, M. Zolkiewski, Walker-A threonine couples nucleotide occupancy with the chaperone activity of the AAA+ ATPase ClpB, Protein Sci 18(2) (2009) 287-93.
[21] A. Matte, L.T.J. Delbaere, ATP-binding Motifs, Encyclopedia of Life Sciences (ELS), John Wiley & Sons, Ltd, Chichester, 2010.
[22] L.M. Iyer, D.D. Leipe, E.V. Koonin, L. Aravind, Evolutionary history and higher order classification of AAA+ ATPases, J. Struct. Biol. 146(1-2) (2004) 11-31.
[23] A.E. Rose, R.S. Brown, C. Schlieker, Torsins: not your typical AAA+ ATPases, Crit Rev Biochem Mol Biol 50(6) (2015) 532-49.
[24] C. Zhao, R.S. Brown, A.R. Chase, M.R. Eisele, C. Schlieker, Regulation of Torsin ATPases by LAP1 and LULL1, Proc Natl Acad Sci U S A 110(17) (2013) E1545-54.

[25] F.E. Demircioglu, B.A. Sosa, J. Ingram, H.L. Ploegh, T.U. Schwartz, Structures of TorsinA and its disease-mutant complexed with an activator reveal the molecular basis for primary dystonia, Elife 5 (2016).
[26] T. Ogura, S.W. Whiteheart, A.J. Wilkinson, Conserved arginine residues implicated in ATP hydrolysis, nucleotide-sensing, and inter-subunit interactions in AAA and AAA+ ATPases, J. Structural Biology 146(1-2) (2004) 106-12.
[27] P. Wendler, S. Ciniawsky, M. Kock, S. Kube, Structure and function of the AAA+ nucleotide binding pocket, Biochim. Biophys. Acta 1823(1) (2012) 2-14.
[28] S.J. Kerns, R.V. Agafonov, Y.J. Cho, F. Pontiggia, R. Otten, D.V. Pachov, S. Kutter, L.A. Phung, P.N. Murphy, V. Thai, T. Alber, M.F. Hagan, D. Kern, The energy landscape of adenylate kinase during catalysis, Nat Struct Mol Biol 22(2) (2015) 124-31.
[29] P. Llinas, T. Isabet, L. Song, V. Ropars, B. Zong, H. Benisty, S. Sirigu, C. Morris, C. Kikuti, D. Safer, H.L. Sweeney, A. Houdusse, How actin initiates the motor activity of Myosin, Dev Cell 33(4) (2015) 401-12.
