# Supplementary material for: Common Mechanism of Activated Catalysis in P-loop Fold Nucleoside Triphosphatases—United in Diversity
Source: Biomolecules. 2022 Sep 22;12(10):1346. doi: 10.3390/biom12101346 (PMC9599734; doi:10.3390/biom12101346)
Supplement: Supplementary file 1 [file biomolecules-12-01346-s001.zip › Biomolecules_1832871_Supplementary_File S1.pdf]

Supplementary File S1 to the manuscript "Common mechanism of activated catalysis in P-loop fold nucleoside triphosphatases – united in diversity" by Maria I. Kozlova, Daria N. Shalaeva, Daria V. Dibrova, Armen Y Mulkidjanian

Classes of P-loop NTPases with ambiguous mechanisms of stimulation and W_{cat} stabilization.

For several large families and even classes of P-loop NTPases, there are no structures with full-fledged catalytic sites containing TS-analogs. Only assumptions can be made on their stimulatory moieties and environments of W_{cat}.

.

Kinase-GTPase Division:

Septins and septin-like proteins **of the TRAFAC class** appear to be stimulated by His residues, which are reciprocally inserted into the catalytic sites upon dimerization of the NTPase domains; the dimerization, supposedly, is induced by the interaction with the activating partner(s) [1, 2]. In septins, the NE2 group of the stimulatory His residue appears to enter the AG site and to link the O^{2A} and O^{3G} atoms [2]. For septins, no TS-like structures are available. Still, in the GNP-containing structure of human septin 12 (PDB ID 6MQ9 [3]) the water molecules next to γ-phosphate interact with NH^{D+3} and residues of the Switch I motif.

ASCE Division:

STAND (*signal transduction ATPases with numerous domains)* ATPases unite apoptotic ATPases (animal apoptosis regulators CED4/Apaf-1, plant disease resistance proteins, and bacterial AfsR-like transcription regulators) and the ATPases of the NACHT superfamily, which consists of the animal disease response ATPases such as CARD4, the NAIP proteins and other ATPases [4]. The Walker B motif of these ATPases contains two consecutive acidic residues [4]. In addition to the P-loop domain, most proteins of the STAND class carry a unique

HETHS domain - the third helical domain, which is present only in this class of proteins [4]. Since some proteins of the STAND class have a conserved Arg residue within the P-loop domain, analogous to the Arg finger of AAA+ ATPases, it was suggested that their activation mechanism is similar to that of AAA+ ATPases [4]. However, available structures of Apaf-1 (Apoptotic protease activating factor 1) suggest a direct involvement of the HETHS domain in the ATP hydrolysis. Structures of Apaf-1 are available for the two states of the protein: the ADP-bound (no Mg^{2+}), "inactive" state (PDB ID 3SFZ [5]), and the "active" state, with Mg-ATP and cytochrome *c* bound (PDB ID 3JBT [6]). It is important to note, that Apaf-1 does not have ATPase activity, so that "active" and "inactive" states differ in the ability of the protein to oligomerize into an apoptosome. In the ATP-bound state, a Tyr residue forms an H-bond with the γ-phosphate (Fig. SF1_1B). In the ADP-bound state, a His residue from the HETHS domain attains a position next to α and β-phosphates and from the same side as Arg/Lys fingers in other P-loop NTPases (Fig. SF1_1B). This His residue is conserved in the majority of STAND proteins [4]. Specifically, a His residue is seen in the similar position in the ADP-containing STAND protein with a tetratricopeptide repeat sensor PH0952 from *Pyrococcus horikoshii* (see Fig. SF1_1C, PDB ID 6MFV [7]). STAND proteins appear to be special; unlike other P-loop NTPases, they convert from a closed form occluding an ADP molecule to an ATP-bound open form prone to multimerize [7]. Hence, Apaf-1 and its homologs cannot serve as a reliable representative for the whole class, especially considering the diversity of domain architectures and the presence of a conserved Arg residue in the P-loop domains of some families. Still, it is tempting to speculate, that the His residue, which is conserved within the STAND class, may be the activating moiety in those enzymes that are capable of hydrolyzing ATP, similarly to aforementioned septins. Structures of STAND ATPases with TS analogs bound are needed for clarification of the activation mechanism and the positioning of $\mathbf{W_{cat}}$.

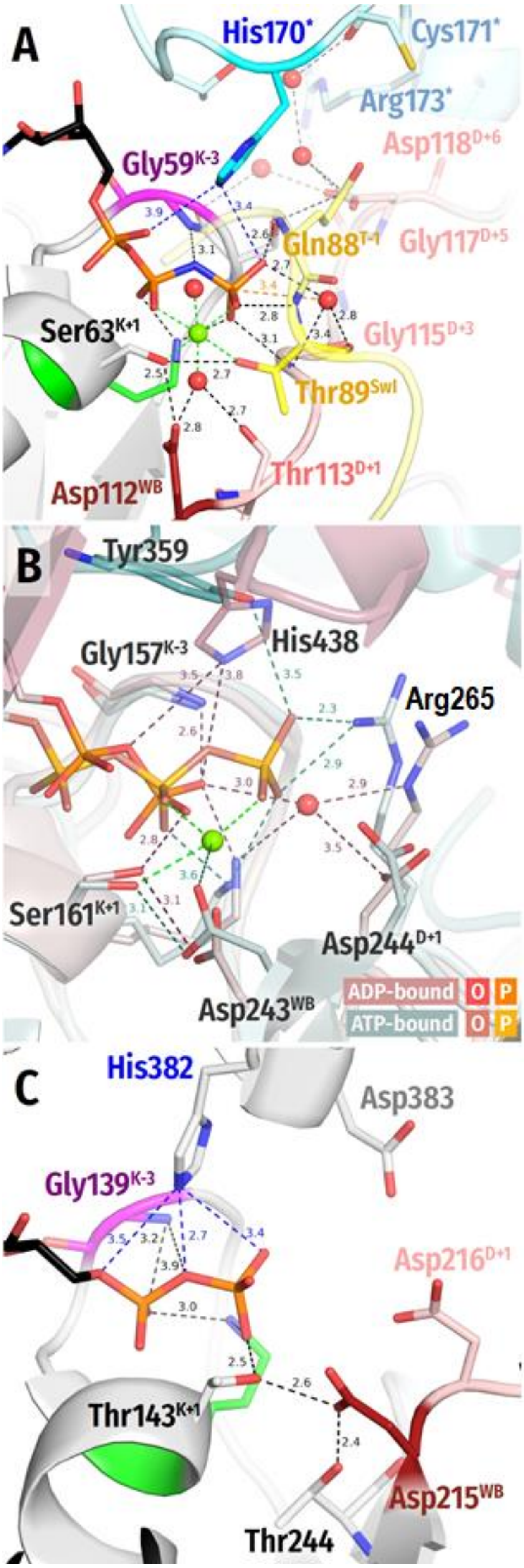

Figure SF1_1. Tentative His fingers in septins and STAND ATPases.

A. Structure of human septin 12 (PDB ID 6MQ9 [3]) complexed with GNP. Protein surrounding NTP-binding site is shown as a cartoon, functionally relevant residues are shown as sticks, water molecules are shown as red spheres, Mg^{2+} as a green sphere. P-loop lysine is shown in green, K-3 residue is shown in magenta. Asp^{WB} is shown in dark red and following residues in light red. Switch 1 residues are shown in yellow, residues belonging to adjacent monomer are highlighted in cyan and marked with an asterisk. All distances are given in ångströms.

B. Superimposed structures of murine apoptotic peptidase activating factor 1 (Apaf-1):

<u>In shades of pink:</u> ADP-bound form (PDB 3SFZ [5]). Water molecule is shown as a red sphere. HETHS domain is shown in a darker shade of pink. The distances are highlighted in dark pink.

<u>In shades of teal:</u> dATP-bound form (PDB ID 3JBT [6]). Mg ion is shown as a green sphere. HETHS domain is shown in a darker shade of teal. The distances are highlighted in dark teal.

C. Structure of the STAND protein with a tetratricopeptide repeat sensor PH0952 from *Pyrococcus horikoshii* (PDB ID 6MFV [7]). Colors as in panel A.

VirB/PilT-like class unites ATPases of the type IVa pili, GspE proteins of the type II secretion system, FlaI proteins of the archaeal flagellar system, and VirB11 proteins of the type IV secretion system [8-13]. In addition to the C-terminal nucleotide-binding P-loop domain, PilT-like ATPases have an N-terminal two-layer α/β sandwich domain, similar to the well-known ligand-binding PAS domain [14]. In the hexameric enzymes, the PAS-like domain provides the Arg finger to the AG site of the bound NTP molecule of the same subunit; an additional Arg finger can interact with γ-phosphate, see the structure in Fig. SF1_2A. In this structure, Ser^{K+1} forms a H-bond with Glu^{E+4} (highlighted in yellow), which, as shown on the panel, is connected to Glu^{WB} by a complex system of water bridges (highlighted in cyan). They resemble those in the PRC (see Fig. 10A in the main text), so that Glu^{WB} is likely to serve as a proton trap in PilT-like NTPases. The Asp^{E+1} residue on the C-cap is far from γ-phosphate. In the absence of TS-like structures, it cannot be fully excluded that Asp^{E+1} comes closer to the ATP molecule upon activation so that Asp^{E+1} interacts with W_{cat}. Alternatively, Glu^{E+4} of the WB-crest can well perform this function and transmit a proton to the buried Glu^{WB}.

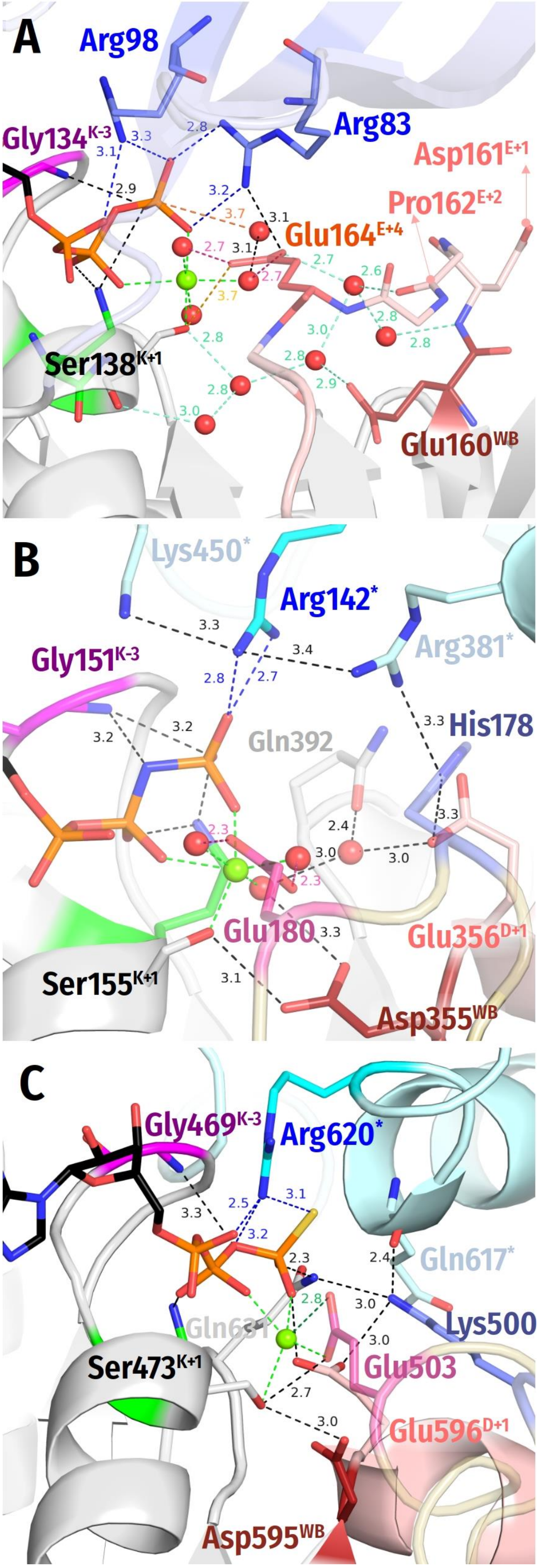

Fig. SF1_2. **Nucleotide-binding sites of P-loop NTPases of VirB/PilT and FtsK-HerA classes.**

A. Structure of the twitching motility pilus retraction ATPase PilT4 from *Geobacter metallireducens*, PDB ID 6OJX [13]). Protein surrounding the NTP-binding site is shown as a cartoon, functionally relevant residues are shown as sticks, water molecules are shown as red spheres, Mg^{2+} as a green sphere. The P-loop lysine is shown in green, the K-3 residue is shown in magenta. Glu^{WB} is shown in dark red and the following residues in pale red. The PAS domain is shown in pale blue and its Arg fingers are shown in blue. All distances are indicated in Å.

B. Crystal structure of the HerA hexameric DNA translocase from *Sulfolobus solfataricus* (PDB 4D2I[15]). Residues belonging to adjacent monomer are highlighted in cyan and marked with an asterisk. The additional Glu residue is shown in pink and the loop harboring it is shown in beige. Other colors as in panel A.

C. Cryo-EM structure of FtsK from *Pseudomonas aeruginosa* PAO1 with resolution of 3.7 Å [16].

Colors as in panel B.

FtsK-HerA pumping ATPases are related to, but not monophyletic with the PilT-like ATPases. The ***HerA ATPases*** are present in all archaea and some bacteria [17] and participate in the repair of double strand DNA breaks [15]. The structure of HerA hexamer from *Sufolobus solfataricus*, in complex with AMP-PNP, a non-hydrolysable analogue of ATP, shows two Arg and one Lys residue pointing towards the triphosphate chain. The Glu355^{WB} residue interacts with Ser155^{K+1} and W6, whereas Glu356^{D+1} points towards the anticipated position of $\mathbf{W_{cat}}$ [15], see Fig. SF1_2B. An additional class-specific Glu residue (Glu180 in the HerA hexamer from *Sufolobus solfataricus*, colored pink), similarly to Glu^{E+4} in PilT4 coordinates W3and W5, Mg^{2+} ligands #3 and #5.

The titular ***FtsK ATPases*** are motor protein that translocate double-stranded DNA during chromosome segregation. A recently reported cryo-EM structure of FtsK of *Pseudomonas aeruginosa* PAO1 with resolution of 3.7 Å indicates that an Arg residue analogous to the arginine finger of the AAA+ superfamily interacts with the NTP-binding site of the adjacent subunit Fig. SF1_2C [16]. The additional Glu residue is also present, but, contrary to HerA, it interacts with Ser^{K+1}, similarly to Glu^{D+4} in PilT-like class ATPases, cf Fig. SF1_2A. The Asp^{WB} is also close, at only 3 Å away of Ser^{K+1}. It is followed by Glu^{D+1} that points towards γ-phosphate and may stabilize W_{cat} in the TS.

Unless TS-like structures of FtsK, HerA, and VirB/PilT-like ATPases with their constricted catalytic sites are obtained, the functions and interactions of their additional Glu residues will remain obscure. Hence, the TS-like, constricted site in any of these ATPases may resemble either that of HerA and FtsK ATPases (where [Asp/Glu]^{WB} interacts with Ser^{K+1}, and the additional Glu interacts with ligands of Mg^{2+}) or that of PilT-like ATPases (where the additional Glu makes a H-bond with Ser^{K+1}). It cannot be fully excluded that two carboxylic groups reach the [Ser/Thr]$^{K+1}$ residue in the TS in the case of these NTPases.

KAP ATPases named after Kidins220/ARMS (mammalian neuronal membrane proteins) and PifA (F-plasmid protein involved in phage T7 exclusion), supposedly, use a AAA-type activation mechanism [18]. The KAP family proteins lack the Arg finger within the P-loop domain proper but carry a conserved arginine in the C-terminal helical segment, which could potentially function as an activating moiety, similar to Sensor 2 of the AAA+ ATPases [18]. Still, in the absence of structures of KAP proteins, both the activation mechanism and the $\mathbf{W_{cat}}$ positioning in enzymes of this class remain unclear.

References

[1] M. Sirajuddin, M. Farkasovsky, E. Zent, A. Wittinghofer, GTP-induced conformational changes in septins and implications for function, Proc. Natl. Acad. Sci. USA 106(39) (2009) 16592-7.
[2] H.V.D. Rosa, D.A. Leonardo, G. Brognara, J. Brandao-Neto, H. D'Muniz Pereira, A.P.U. Araujo, R.C. Garratt, Molecular Recognition at Septin Interfaces: The Switches Hold the Key, J Mol Biol 432(21) (2020) 5784-5801.
[3] D. Castro, S.M.O. da Silva, H.D. Pereira, J.N.A. Macedo, D.A. Leonardo, N.F. Valadares, P.S. Kumagai, J. Brandao-Neto, A.P.U. Araujo, R.C. Garratt, A complete compendium of crystal structures for the human SEPT3 subgroup reveals functional plasticity at a specific septin interface, IUCrJ 7(Pt 3) (2020) 462-479.
[4] D.D. Leipe, E.V. Koonin, L. Aravind, STAND, a class of P-loop NTPases including animal and plant regulators of programmed cell death: multiple, complex domain architectures, unusual phyletic patterns, and evolution by horizontal gene transfer, J. Mol. Biol. 343(1) (2004) 1-28.
[5] T.F. Reubold, S. Wohlgemuth, S. Eschenburg, Crystal structure of full-length Apaf-1: how the death signal is relayed in the mitochondrial pathway of apoptosis, Structure 19(8) (2011) 1074-83.
[6] M. Zhou, Y. Li, Q. Hu, X.C. Bai, W. Huang, C. Yan, S.H. Scheres, Y. Shi, Atomic structure of the apoptosome: mechanism of cytochrome c- and dATP-mediated activation of Apaf-1, Genes Dev 29(22) (2015) 2349-61.
[7] M.N. Lisa, V. Cvirkaite-Krupovic, E. Richet, G. Andre-Leroux, P.M. Alzari, A. Haouz, O. Danot, Double autoinhibition mechanism of signal transduction ATPases with numerous domains (STAND) with a tetratricopeptide repeat sensor, Nucleic Acids Res 47(7) (2019) 3795-3810.
[8] P.J. Planet, S.C. Kachlany, R. DeSalle, D.H. Figurski, Phylogeny of genes for secretion NTPases: identification of the widespread tadA subfamily and development of a diagnostic key for gene classification, Proc Natl Acad Sci U S A 98(5) (2001) 2503-8.
[9] K.G. Aukema, E.M. Kron, T.J. Herdendorf, K.T. Forest, Functional dissection of a conserved motif within the pilus retraction protein PilT, J Bacteriol 187(2) (2005) 611-8.
[10] S.N. Savvides, H.J. Yeo, M.R. Beck, F. Blaesing, R. Lurz, E. Lanka, R. Buhrdorf, W. Fischer, R. Haas, G. Waksman, VirB11 ATPases are dynamic hexameric assemblies: new insights into bacterial type IV secretion, EMBO J 22(9) (2003) 1969-80.

[11] K.A. Satyshur, G.A. Worzalla, L.S. Meyer, E.K. Heiniger, K.G. Aukema, A.M. Misic, K.T. Forest, Crystal structures of the pilus retraction motor PilT suggest large domain movements and subunit cooperation drive motility, Structure 15(3) (2007) 363-376.
[12] J.M. Mancl, W.P. Black, H. Robinson, Z. Yang, F.D. Schubot, Crystal Structure of a Type IV Pilus Assembly ATPase: Insights into the Molecular Mechanism of PilB from Thermus thermophilus, Structure 24(11) (2016) 1886-1897.
[13] M. McCallum, S. Benlekbir, S. Nguyen, S. Tammam, J.L. Rubinstein, L.L. Burrows, P.L. Howell, Multiple conformations facilitate PilT function in the type IV pilus, Nat Commun 10(1) (2019) 5198.
[14] C.P. Ponting, L. Aravind, PAS: a multifunctional domain family comes to light, Curr Biol 7(11) (1997) R674-7.
[15] N.J. Rzechorzek, J.K. Blackwood, S.M. Bray, J.D. Maman, L. Pellegrini, N.P. Robinson, Structure of the hexameric HerA ATPase reveals a mechanism of translocation-coupled DNA-end processing in archaea, Nat Commun 5 (2014) 5506.
[16] N.L. Jean, T.J. Rutherford, J. Lowe, FtsK in motion reveals its mechanism for double-stranded DNA translocation, Proc Natl Acad Sci U S A 117(25) (2020) 14202-14208.
[17] L.M. Iyer, K.S. Makarova, E.V. Koonin, L. Aravind, Comparative genomics of the FtsK-HerA superfamily of pumping ATPases: implications for the origins of chromosome segregation, cell division and viral capsid packaging, Nucleic Acids Res 32(17) (2004) 5260-79.
[18] L. Aravind, L.M. Iyer, D.D. Leipe, E.V. Koonin, A novel family of P-loop NTPases with an unusual phyletic distribution and transmembrane segments inserted within the NTPase domain, Genome Biol. 5(5) (2004) R30.
