# Supplementary material for: Common Mechanism of Activated Catalysis in P-loop Fold Nucleoside Triphosphatases—United in Diversity
Source: Biomolecules. 2022 Sep 22;12(10):1346. doi: 10.3390/biom12101346 (PMC9599734; doi:10.3390/biom12101346)
Supplement: Supplementary file 1 [file biomolecules-12-01346-s001.zip › Biomolecules_1832871_Supplementary_File S2.pdf]

Supplementary File S2 to the manuscript "Common mechanism of activated catalysis in P-loop fold nucleoside triphosphatases – united in diversity"

by Maria I. Kozlova, Daria N. Shalaeva, Daria V. Dibrova, and Armen Y Mulkidjanian

Transition state analogs and energetics of P-loop NTPases

The observation of the shortest H-bonds within the catalytic sites in the presence of NDP:AlF_4^- (see Fig. 8, Table S1 and S2) helps to clarify why these complexes are most potent functional TS-mimics in P-loop NTPases [1-19]. Beginning in 1987, that is, even before the first GDP:AlF_4^--containing structures were resolved in 1994 [11,12], metal fluorides were shown to promote binding of various P-loop NTPases to their activating partners. Generally, many NTPases interacted with their activators only in the presence of metal fluorides [1-3,6-10,18-21]. Crystal structures of P-loop NTPases with NDP:AlF_4^- or NDP:AlF_3/NDP:MgF_3^- complexes bound [4-8,11-13,17] show that metal-fluorides have planar (AlF_4^- and MgF_3^-) or almost planar (NDP:AlF_3) structures with W_{cat} in the apical attack position, see respective panels in Fig. 1-6 and the companion paper [22]. While the shape and electric charge of MgF_3^- is the same as that of γ-phosphate in the anticipated TS (see Fig. S1 of the main text), the shape of AlF_4^- differs significantly, it is tetragonal instead of trigonal. Nevertheless, NDP:AlF_4^- complexes are more potent functional analogs of the TS than NDP:MgF_3^- or NDP:AlF_3, as follows from their superiority in promoting binding of P-loop NTPases to their activators [1-3,6-10,18-21]. In addition, the distances between W_{cat} and the metal atom are shorter (2.0-2.1 Å) in the case of NDP:AlF_4^- complexes than NDP:MgF_3^- (approx. 2.5 Å) or NDP:AlF_3 complexes (approx. 3.0 Å) see the companion article [22] and [6,7]. The reason why NDP:AlF_4^- performs better than other substrate and TS analogs in all these cases has remained obscure, see e.g. [19].

The reason could be, however, understood by considering the energetics of P-loop NTPases. Our earlier MD simulations have shown that the linking of α- and γ-phosphates by an inserted stimulatory cationic moiety is an endergonic reaction; about 20-25 kJ/mol appear to be needed to twist the γ-phosphate by straining its bonds with Mg^{2+} and Lys^{WA} [23,24]. This activation barrier prevents haphazard NTP hydrolysis due to accidental insertion of a K^+ ion or an Arg residue in the catalytic site, as argued elsewhere [23,24]. These estimates from MD simulations corroborate earlier considerations of Warshel and colleagues that the stimulation of hydrolysis in P-loop NTPases by their cognate activators requires an input of about 20-25 kJ/mol of free energy, see e.g. [25,26].

A specific problem of P-loop NTPases is the source of a such free energy input. In most enzymes, the free energy for lowering the activation barrier – by destabilizing the substrate-bound ground state and/or stabilizing the TS – is usually provided by substrate binding [27]. In P-loop NTPases, where the substrate binding step is separated from the ultimate catalytic step in most cases, the energy of substrate binding is partly used to bring the NTP molecule into its extended, catalytically prone conformation with eclipsed β- and γ-phosphates [28-30]. Another part of the binding energy is used to pre-organize the catalytic site, with the conformational changes that accompany catalytic site closure often being coupled with useful mechanical work, e.g. in myosins [31,32]. While increasing the rate of NTP hydrolysis as compared to that in water (by five orders of magnitude in the case of Ras GTPase [33,34]), the free energy of NTP binding to the P-loop is spent without achieving physiologically relevant hydrolysis rates. For the further acceleration of hydrolysis, additional source(s) of free energy is/are needed.

The source of additional free energy is evident in the case of ring-forming oligomeric P-loop NTPases, such as many AAA+ ATPases (see Fig. 5A of the main text and [35,36]), helicases (see Fig. 5B-C of the main text and [37,38]), or rotary ATPases/synthases of the RecA/F_1 class (see Fig. 6A of the main text and [39-41]). In such complexes, the free energy for the cleavage of a bound ATP molecule in one monomer is provided by the binding of another ATP molecule to another monomer. Part of the substrate binding energy at this other site drives a molecular motion that is transferred, for example, to the Arg finger; in the case of rotational F_1-ATPases, Paul Boyer called this kind of coupling "binding change mechanism" [41].

The same reasoning can be applied to ABC transporters (Fig. 5D in the main text), where ATP-hydrolysis appears to be coupled to the binding of the translocated molecule. Also, the activation of kinases (Fig. 4C-D in the main text) appears to be driven by binding of their phosphate-accepting substrates.

In other cases, however, P-loop NTPases, with an NTP molecule bound, do not bind further small molecules, but only the cognate activating partners, which are protein/RNA/DNA molecules (Table 1). These NTPases, per exclusionem, can harness only the free energy of binding to their activating partner(s). Indeed, there is evidence that the strength of such protein-protein binding does affect the catalytic activity of some P-loop NTPases [42-45], making them easy to regulate via protein-protein interactions, as observed with small GTPases.

In all these cases, we are dealing with thermodynamic coupling where the free energy of binding is used for endergonic constriction of the catalytic site. Such a thermodynamic coupling is reversible by definition. Consequently, if we find a way to exergonically constrict the catalytic site, it will lead to the spontaneous assembly of the complex between P-loop NTPase and its

cognate activator(s). Apparently, the binding of TS analogs such as NDP:AlF_4^- or NDP:MgF_3^-, which form multiple strong bonds within the catalytic site, is an exergonic reaction that leads to a TS-like configuration of the catalytic site by "constricting it from the inside", which can happen even in the absence of an activating partner (as in the NDP:AlF_4^--containing separately crystallized dimer of GTPase domains of the MnmE protein (Fig. 1F in the companion paper [22]) or SIMIBI NTPases (Fig. 4A-B in the main text). If cognate activators are also present, the "from-the-inside constriction" drives their interaction with the NTPase domain yielding a full-fledged activated complex, as described in the literature [1-3,6-10,18-21].

If this assumption is correct, then the assembling "efficiency" of the TS analogs will depend on the amount of free energy that becomes available upon their binding. As it follows from Fig. 8 of the main text and Fig. 3 of the companion paper {Kozlova, 2022 #3498}, fluoride complexes are superior to vanadate complexes in their ability to constrict the catalytic site. This is consistent with the fact that the more electronegative fluorine atoms form stronger H-bonds than oxygen atoms. Also NDP:AlF_4^- complexes are superior to NDP:MgF_3^- complexes (Fig. 8 of the main textand Fig. 2 of the companion paper [22]) because, as documented in Table S2, they enter into more bonds. Not surprisingly, NDP:AlF_4^- complexes, which "raise" more binding energy despite their "unphysiological" geometry, are the most efficient in assembling activated complexes *in vitro*.

In sum, the extent of H-bonding of TS analogs within catalytic sites of P-loop NTPases (Fig. 8 of the main text, Tables S1, S2 and Fig. 3 of the companion paper [22]) appear to correlate with their ability to induce self-assembly of activated complexes [1-3,6-10,18-21], which implies that these H-bonds stabilize the TS upon hydrolysis of ATP or GTP.

The abundance and variety of such auxiliary interactions around the γ-phosphate and WM-crest, as described in the main text and the companion article {Kozlova, 2022 #3498}, could be related to the energetics of P-loop NTPases. Many of H-bonding interactions between oppositely charged moieties of the P-loop domain and its activator, which often invoke residues of WB-crest, may provide the free energy needed for catalysis. If so, elimination – for example through mutations – of such interactions, even if they are not involved in the chemistry of catalysis, would still slow the enzyme reaction, which is consistent with some experimental observations [18,42,45]. Therefore, a case-by-case analysis is needed to understand whether specific auxiliary residues contribute directly to catalysis or only to the exergonic interaction of the NTPase domain with its activator.

References

1. Zhang, N.; Buck, M. Formation of MgF3 (-)-dependent complexes between an AAA(+) ATPase and sigma(54.). *FEBS Open Bio* **2012**, *2*, 89-92.
2. Chaney, M.; Grande, R.; Wigneshweraraj, S.R.; Cannon, W.; Casaz, P.; Gallegos, M.T.; Schumacher, J.; Jones, S.; Elderkin, S.; Dago, A.E.; et al. Binding of transcriptional activators to sigma 54 in the presence of the transition state analog ADP-aluminum fluoride: insights into activator mechanochemical action. *Genes Dev* **2001**, *15*, 2282-2294.
3. Higashijima, T.; Ferguson, K.M.; Sternweis, P.C.; Ross, E.M.; Smigel, M.D.; Gilman, A.G. The effect of activating ligands on the intrinsic fluorescence of guanine nucleotide-binding regulatory proteins. *J Biol Chem* **1987**, *262*, 752-756.
4. Graham, D.L.; Lowe, P.N.; Grime, G.W.; Marsh, M.; Rittinger, K.; Smerdon, S.J.; Gamblin, S.J.; Eccleston, J.F. MgF(3)(-) as a transition state analog of phosphoryl transfer. *Chem Biol* **2002**, *9*, 375-381.
5. Schlichting, I.; Reinstein, J. pH influences fluoride coordination number of the AlFx phosphoryl transfer transition state analog. *Nat Struct Biol* **1999**, *6*, 721-723.
6. Jin, Y.; Molt, R.W., Jr.; Blackburn, G.M. Metal fluorides: Tools for structural and computational analysis of phosphoryl transfer enzymes. *Top. Curr. Chem. (Cham)* **2017**, *375*, 36.
7. Jin, Y.; Richards, N.G.; Waltho, J.P.; Blackburn, G.M. Metal fluorides as analogues for studies on phosphoryl transfer enzymes. *Angew. Chem. Int. Ed. Engl.* **2017**, *56*, 4110-4128.
8. Scheffzek, K.; Ahmadian, M.R.; Kabsch, W.; Wiesmuller, L.; Lautwein, A.; Schmitz, F.; Wittinghofer, A. The Ras-RasGAP complex: structural basis for GTPase activation and its loss in oncogenic Ras mutants. *Science* **1997**, *277*, 333-338.
9. Gremer, L.; Gilsbach, B.; Ahmadian, M.R.; Wittinghofer, A. Fluoride complexes of oncogenic Ras mutants to study the Ras-RasGap interaction. *Biol. Chem.* **2008**, *389*, 1163-1171.
10. Wittinghofer, A. Signaling mechanistics: aluminum fluoride for molecule of the year. *Curr. Biol.* **1997**, *7*, R682-R685.
11. Coleman, D.E.; Berghuis, A.M.; Lee, E.; Linder, M.E.; Gilman, A.G.; Sprang, S.R. Structures of active conformations of Gi alpha 1 and the mechanism of GTP hydrolysis. *Science* **1994**, *265*, 1405-1412.
12. Sondek, J.; Lambright, D.G.; Noel, J.P.; Hamm, H.E.; Sigler, P.B. GTPase mechanism of Gproteins from the 1.7-A crystal structure of transducin alpha-GDP-AIF-4. *Nature* **1994**, *372*, 276-279.
13. Menz, R.I.; Walker, J.E.; Leslie, A.G. Structure of bovine mitochondrial F_1-ATPase with nucleotide bound to all three catalytic sites: implications for the mechanism of rotary catalysis. *Cell* **2001**, *106*, 331-341.
14. Chabre, M. Aluminofluoride and beryllofluoride complexes: new phosphate analogs in enzymology. *Trends in biochemical sciences* **1990**, *15*, 6-10.
15. Antonny, B.; Bigay, J.; Chabre, M. A novel magnesium-dependent mechanism for the activation of transducin by fluoride. *FEBS Lett* **1990**, *268*, 277-280.
16. Graham, D.L.; Eccleston, J.F.; Chung, C.W.; Lowe, P.N. Magnesium fluoride-dependent binding of small G proteins to their GTPase-activating proteins. *Biochemistry* **1999**, *38*, 14981-14987.
17. Antonny, B.; Sukumar, M.; Bigay, J.; Chabre, M.; Higashijima, T. The mechanism of aluminum-independent G-protein activation by fluoride and magnesium. 31P NMR spectroscopy and fluorescence kinetic studies. *J Biol Chem* **1993**, *268*, 2393-2402.
18. Gasper, R.; Wittinghofer, F. The Ras switch in structural and historical perspective. *Biol Chem* **2019**, *401*, 143-163.
19. Baxter, N.J.; Blackburn, G.M.; Marston, J.P.; Hounslow, A.M.; Cliff, M.J.; Bermel, W.; Williams, N.H.; Hollfelder, F.; Wemmer, D.E.; Waltho, J.P. Anionic charge is prioritized over geometry in aluminum and magnesium fluoride transition state analogs of phosphoryl transfer enzymes. *J Am Chem Soc* **2008**, *130*, 3952-3958.
20. Mittal, R.; Ahmadian, M.R.; Goody, R.S.; Wittinghofer, A. Formation of a transition-state analog of the Ras GTPase reaction by Ras-GDP, tetrafluoroaluminate, and GTPase-activating proteins. *Science* **1996**, *273*, 115-117.
21. Chakrabarti, P.P.; Daumke, O.; Suveyzdis, Y.; Kotting, C.; Gerwert, K.; Wittinghofer, A. Insight into catalysis of a unique GTPase reaction by a combined biochemical and FTIR approach. *J Mol Biol* **2007**, *367*, 983-995.
22. Kozlova, M.I.; Shalaeva, D.N.; Dibrova, D.V.; Mulkidjanian, A.Y. Patterns of hydrolysis initiation in P-loop fold nucleoside triphosphatases. *BioRxiv* **2022**.

23. Shalaeva, D.N.; Cherepanov, D.A.; Galperin, M.Y.; Golovin, A.V.; Mulkidjanian, A.Y. Evolution of cation binding in the active sites of P-loop nucleoside triphosphatases in relation to the basic catalytic mechanism. *Elife* **2018**, *7*.
24. Shalaeva, D.N.; Cherepanov, D.A.; Galperin, M.Y.; Mulkidjanian, A.Y. Comparative analysis of active sites in P-loop nucleoside triphosphatases suggests an ancestral activation mechanism. *BioRxiv* **2018**, *439992 [preprint]*.
25. Glennon, T.M.; Villa, J.; Warshel, A. How does GAP catalyze the GTPase reaction of Ras?: A computer simulation study. *Biochemistry* **2000**, *39*, 9641-9651.
26. Kamerlin, S.C.; Sharma, P.K.; Prasad, R.B.; Warshel, A. Why nature really chose phosphate. *Q. Rev. Biophys.* **2013**, *46*, 1-132.
27. Jencks, W.P. *Catalysis in chemistry and enzymology*; Courier Corporation: 1987.
28. Matte, A.; Tari, L.W.; Delbaere, L.T. How do kinases transfer phosphoryl groups? *Structure* **1998**, *6*, 413-419.
29. Delbaere, L.T.; Sudom, A.M.; Prasad, L.; Leduc, Y.; Goldie, H. Structure/function studies of phosphoryl transfer by phosphoenolpyruvate carboxykinase. *Biochim. Biophys. Acta* **2004**, *1697*, 271-278.
30. Allin, C.; Gerwert, K. Ras catalyzes GTP hydrolysis by shifting negative charges from gamma- to beta-phosphate as revealed by time-resolved FTIR difference spectroscopy. *Biochemistry* **2001**, *40*, 3037-3046.
31. Geeves, M.A. Review: The ATPase mechanism of myosin and actomyosin. *Biopolymers* **2016**, *105*, 483-491.
32. Frye, J.J.; Klenchin, V.A.; Bagshaw, C.R.; Rayment, I. Insights into the importance of hydrogen bonding in the gamma-phosphate binding pocket of myosin: structural and functional studies of serine 236. *Biochemistry* **2010**, *49*, 4897-4907.
33. Shutes, A.; Der, C.J. Real-time in vitro measurement of intrinsic and Ras GAP-mediated GTP hydrolysis. *Methods Enzymol* **2006**, *407*, 9-22.
34. Kotting, C.; Gerwert, K. Time-resolved FTIR studies provide activation free energy, activation enthalpy and activation entropy for GTPase reactions. *Chem Phys* **2004**, *307*, 227-232.
35. Hattendorf, D.A.; Lindquist, S.L. Analysis of the AAA sensor-2 motif in the C-terminal ATPase domain of Hsp104 with a site-specific fluorescent probe of nucleotide binding. *Proc. Natl. Acad. Sci. USA* **2002**, *99*, 2732-2737.
36. Lupas, A.N.; Martin, J. AAA proteins. *Curr. Opin. Struct. Biol.* **2002**, *12*, 746-753.
37. Singleton, M.R.; Dillingham, M.S.; Wigley, D.B. Structure and mechanism of helicases and nucleic acid translocases. *Annu Rev Biochem* **2007**, *76*, 23-50.
38. Thomsen, N.D.; Berger, J.M. Structural frameworks for considering microbial protein- and nucleic acid-dependent motor ATPases. *Mol Microbiol* **2008**, *69*, 1071-1090.
39. Walker, J.E. The regulation of catalysis in ATP synthase. *Curr Opin Struct Biol* **1994**, *4*, 912-918.
40. Senior, A.E.; Nadanaciva, S.; Weber, J. The molecular mechanism of ATP synthesis by F_1F_0-ATP synthase. *Biochim. Biophys. Acta* **2002**, *1553*, 188-211.
41. Boyer, P.D. The ATP synthase--a splendid molecular machine. *Annu. Rev. Biochem.* **1997**, *66*, 717-749.
42. Gideon, P.; John, J.; Frech, M.; Lautwein, A.; Clark, R.; Scheffler, J.E.; Wittinghofer, A. Mutational and kinetic analyses of the GTPase-activating protein (GAP)-p21 interaction: The C-terminal domain of GAP is not sufficient for full activity. *Mol. Cell Biol.* **1992**, *12*, 2050-2056.
43. Codina, J.; Birnbaumer, L. Requirement for intramolecular domain interaction in activation of G protein alpha subunit by aluminum fluoride and GDP but not by GTP gamma S. *J. Biol. Chem.* **1994**, *269*, 29339-29342.
44. Cherfils, J.; Zeghouf, M. Regulation of small GTPases by GEFs, GAPs, and GDIs. *Physiol. Rev.* **2013**, *93*, 269-309.
45. Smith, C.A.; Rayment, I. Active site comparisons highlight structural similarities between myosin and other P-loop proteins. *Biophys J* **1996**, *70*, 1590-1602.
