# Supplementary material for: Common Mechanism of Activated Catalysis in P-loop Fold Nucleoside Triphosphatases—United in Diversity
Source: Biomolecules. 2022 Sep 22;12(10):1346. doi: 10.3390/biom12101346 (PMC9599734; doi:10.3390/biom12101346)
Supplement: Supplementary file 1 [file biomolecules-12-01346-s001.zip › Biomolecules_1832871_Supplementary_File S3.pdf]

Supplementary File S3 to the manuscript "Common mechanism of activated catalysis in P-loop fold nucleoside triphosphatases – united in diversity"

by Maria I. Kozlova, Daria N. Shalaeva, Daria V. Dibrova,

and Armen Y Mulkidjanian

Local molecular electric field in the catalytic sites of P-loop NTPases

In structures of P-loop NTPases that are shown in Fig. 1-7, the positive charged groups that stabilize the triphosphate chain, namely the Lys^{WA} residue, the backbone **HN** groups, the stimulatory moiety(ies), the auxiliary residues, and the positively charged N-terminus of the first α-helix are opposed by acidic residues that interact with W_{cat} either directly or via water bridges. Together with Asp^{WB}, these catalytic acidic residues form negatively charged clusters. Fig. SF3_1 below shows, for diverse P-loop NTPases, these positively and negatively charged clusters, which should produce a strong local electric field.

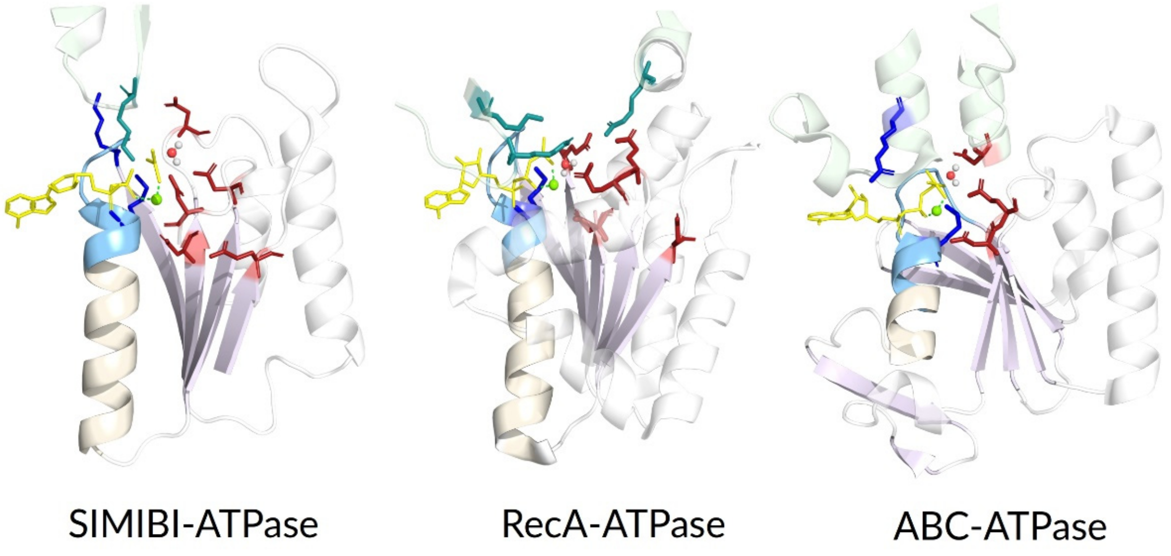

Figure S3. Uneven distribution of charged residues around nucleotide binding pockets in different classes of P-loop NTPases. Nucleotide analogs are shown in yellow, negatively charged residues are shown in red, positively charged residues are shown in blue and teal, the α_1 helix is shown in beige with its N-terminus and the P-loop shown in light blue.

A, SIMIBI class, light-independent protochlorophyllide reductase iron-sulfur ATP-binding protein chlL) (PDB ID 2YNM, [1]); **B**, RecA class, circadian clock protein kinase KaiC (PDB ID 4TL7, [2]); **C**, ABC-ATPase class, MBP-maltose transporter complex (PDB ID 3PUW,[3]).

Hence, the electrostatic potential at the catalytic sites of diverse P-loop NTPases is distributed unevenly, which has already been noted for particular enzymes of this family, see, e.g., [4]. The strength of the local electric field can be roughly estimated using the Coulomb's equation as ~ 10^8 V/m under the very modest assumptions that (i) the resulting electric charge difference at a distance of 10 Å (between the acid residues and the P-loop) corresponds to one elementary charge and (ii) the effective dielectric permittivity of the catalytic pocket is about 10 [5,6]. Such an electric field strength, albeit large, is compatible with those measured in the catalytic pockets of other enzymes [7].

Notably, in those cases where the stimulator is positively charged, its positive charge not only secures the bonding with particular oxygen atom(s) of triphosphate and increases the positive charge on P^G, but additionally polarizes the whole catalytic pocket.

Consequently, the local electric field is directed approximately from the P-loop to Asp^{WB}, which should drive a proton from W_{cat} to Asp^{WB}. Hence, the catalytic pocket is strongly polarized in P-loop NTPases, which also can contribute to catalysis, as discussed below.

References

1. Moser, J.; Lange, C.; Krausze, J.; Rebelein, J.; Schubert, W.D.; Ribbe, M.W.; Heinz, D.W.; Jahn, D. Structure of ADP-aluminium fluoride-stabilized protochlorophyllide oxidoreductase complex. *Proc Natl Acad Sci U S A* **2013**, *110*, 2094-2098.
2. Abe, J.; Hiyama, T.B.; Mukaiyama, A.; Son, S.; Mori, T.; Saito, S.; Osako, M.; Wolanin, J.; Yamashita, E.; Kondo, T.; et al. Circadian rhythms. Atomic-scale origins of slowness in the cyanobacterial circadian clock. *Science* **2015**, *349*, 312-316.
3. Oldham, M.L.; Chen, J. Snapshots of the maltose transporter during ATP hydrolysis. *Proc Natl Acad Sci U S A* **2011**, *108*, 15152-15156.
4. delToro, D.; Ortiz, D.; Ordyan, M.; Pajak, J.; Sippy, J.; Catala, A.; Oh, C.S.; Vu, A.; Arya, G.; Smith, D.E.; et al. Functional Dissection of a Viral DNA Packaging Machine's Walker B Motif. *J Mol Biol* **2019**, *431*, 4455-4474.
5. Garcia-Moreno, B.; Dwyer, J.J.; Gittis, A.G.; Lattman, E.E.; Spencer, D.S.; Stites, W.E. Experimental measurement of the effective dielectric in the hydrophobic core of a protein. *Biophys Chem* **1997**, *64*, 211-224.
6. Kukic, P.; Farrell, D.; McIntosh, L.P.; Garcia-Moreno, E.B.; Jensen, K.S.; Toleikis, Z.; Teilum, K.; Nielsen, J.E. Protein dielectric constants determined from NMR chemical shift perturbations. *J Am Chem Soc* **2013**, *135*, 16968-16976.
7. Fried, S.D.; Bagchi, S.; Boxer, S.G. Extreme electric fields power catalysis in the active site of ketosteroid isomerase. *Science* **2014**, *346*, 1510-1514.
