# Supplementary material for: Common Mechanism of Activated Catalysis in P-loop Fold Nucleoside Triphosphatases—United in Diversity
Source: Biomolecules. 2022 Sep 22;12(10):1346. doi: 10.3390/biom12101346 (PMC9599734; doi:10.3390/biom12101346)
Supplement: Supplementary file 1 [file biomolecules-12-01346-s001.zip › biomolecules_1832871_Supplementary_Table S3_ATP_synthase.pdf]

Supplementary Table S3 to the manuscript "Common mechanism of activated catalysis in P-loop fold nucleoside triphosphatases – united in diversity"

by Maria I. Kozlova, Daria N. Shalaeva, Daria V. Dibrova,
and Armen Y Mulkidjanian

The pKa values of Asp256 WB of the bovine ATP synthase as calculated for different states of the catalytic pocket by application of the PROPKA v. 2.0 software [1]

PDB ID, res.	ADP:AlF_4^-	ADP:AlF_3	ADP:BeF_3	ANP	ATP	ADP+P_i	ADP	P_i	Empty	Ref
1E1R, 2.5 Å								**1.82**		**[2]**
1E1R, 2.5 Å				**9.64**						**[2]**
1e1R, 2.5 Å		**9.46**								**[2]**
1E1Q, 2.6 Å							**9.03**			**[2]**
1E1Q, 2.6 Å								**2,39**		**[2]**
1E1Q, 2.6 Å				**8.92**						**[2]**
1H8E, 2.0 Å	**8.98**									**[3]**
1H8E, 2.0 Å						**5.41**				**[3]**
1H8E, 2.0 Å	**8.83**									**[3]**
1W0J, 2.2 Å			**9.43**							**[4]**
1W0J, 2.2 Å								**2.17**		**[4]**
1W0J, 2.2 Å			**9.11**							**[4]**
1H8H, 2.9 Å							**9.10**			**[5]**
1H8H, 2.9 Å								**2.33**		**[5]**
1H8H, 2.9 Å					**8.76**					**[5]**
2CK3, 1.9 Å							**9.25**			**[6]**
2CK3, 1.9 Å				**9.20**						**[6]**
2CK3, 1.9 Å								**2.93**		**[6]**
2JDI, 1.9 Å				**9.39**						**[7]**
2JDI, 1.9 Å									**2.97**	**[7]**
2JDI, 1.9 Å				**9.32**						**[7]**
2JIZ, 2.3 Å							**9.51**			**[8]**
2JIZ, 2.3 Å								**2.07**		**[8]**
2JIZ, 2.3 Å				**9.26**						**[8]**

References

1. Bas, D.C.; Rogers, D.M.; Jensen, J.H. Very fast prediction and rationalization of pKa values for protein-ligand complexes. *Proteins* **2008**, *73*, 765-783.
2. Braig, K.; Menz, R.I.; Montgomery, M.G.; Leslie, A.G.; Walker, J.E. Structure of bovine mitochondrial F(1)-ATPase inhibited by Mg(2+) ADP and aluminium fluoride. *Structure* **2000**, *8*, 567-573.
3. Menz, R.I.; Walker, J.E.; Leslie, A.G. Structure of bovine mitochondrial F_1-ATPase with nucleotide bound to all three catalytic sites: implications for the mechanism of rotary catalysis. *Cell* **2001**, *106*, 331-341.
4. Kagawa, R.; Montgomery, M.G.; Braig, K.; Leslie, A.G.; Walker, J.E. The structure of bovine F1-ATPase inhibited by ADP and beryllium fluoride. *EMBO J* **2004**, *23*, 2734-2744.
5. Menz, R.I.; Leslie, A.G.; Walker, J.E. The structure and nucleotide occupancy of bovine mitochondrial F(1)-ATPase are not influenced by crystallisation at high concentrations of nucleotide. *FEBS Lett* **2001**, *494*, 11-14.
6. Bowler, M.W.; Montgomery, M.G.; Leslie, A.G.; Walker, J.E. How azide inhibits ATP hydrolysis by the F-ATPases. *Proc Natl Acad Sci U S A* **2006**, *103*, 8646-8649.
7. Bowler, M.W.; Montgomery, M.G.; Leslie, A.G.; Walker, J.E. Ground state structure of F1-ATPase from bovine heart mitochondria at 1.9 A resolution. *J Biol Chem* **2007**, *282*, 14238-14242.
8. Gledhill, J.R.; Montgomery, M.G.; Leslie, A.G.; Walker, J.E. Mechanism of inhibition of bovine F1-ATPase by resveratrol and related polyphenols. *Proc Natl Acad Sci U S A* **2007**, *104*, 13632-13637.
